# Supplementary figures and images for: Dehydration alters behavioral thermoregulation and the geography of climatic vulnerability in two Amazonian lizards
Source: PLoS One. 2023 Nov 1;18(11):e0286502. doi: 10.1371/journal.pone.0286502 (PMC10619801; doi:10.1371/journal.pone.0286502)

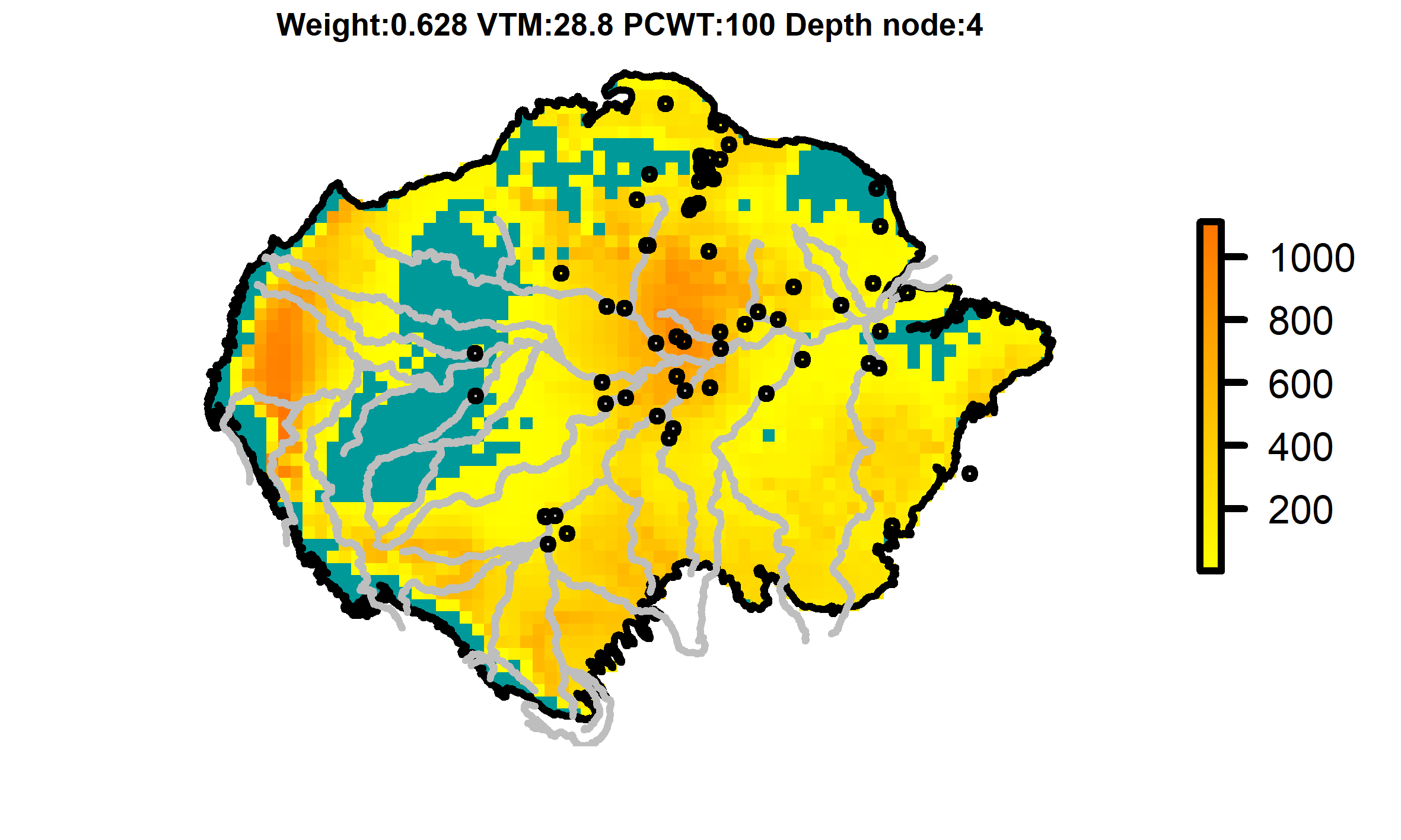

Supplement: S1 Data — (ZIP) [file pone.0286502.s002.zip › maps/map 46.png]

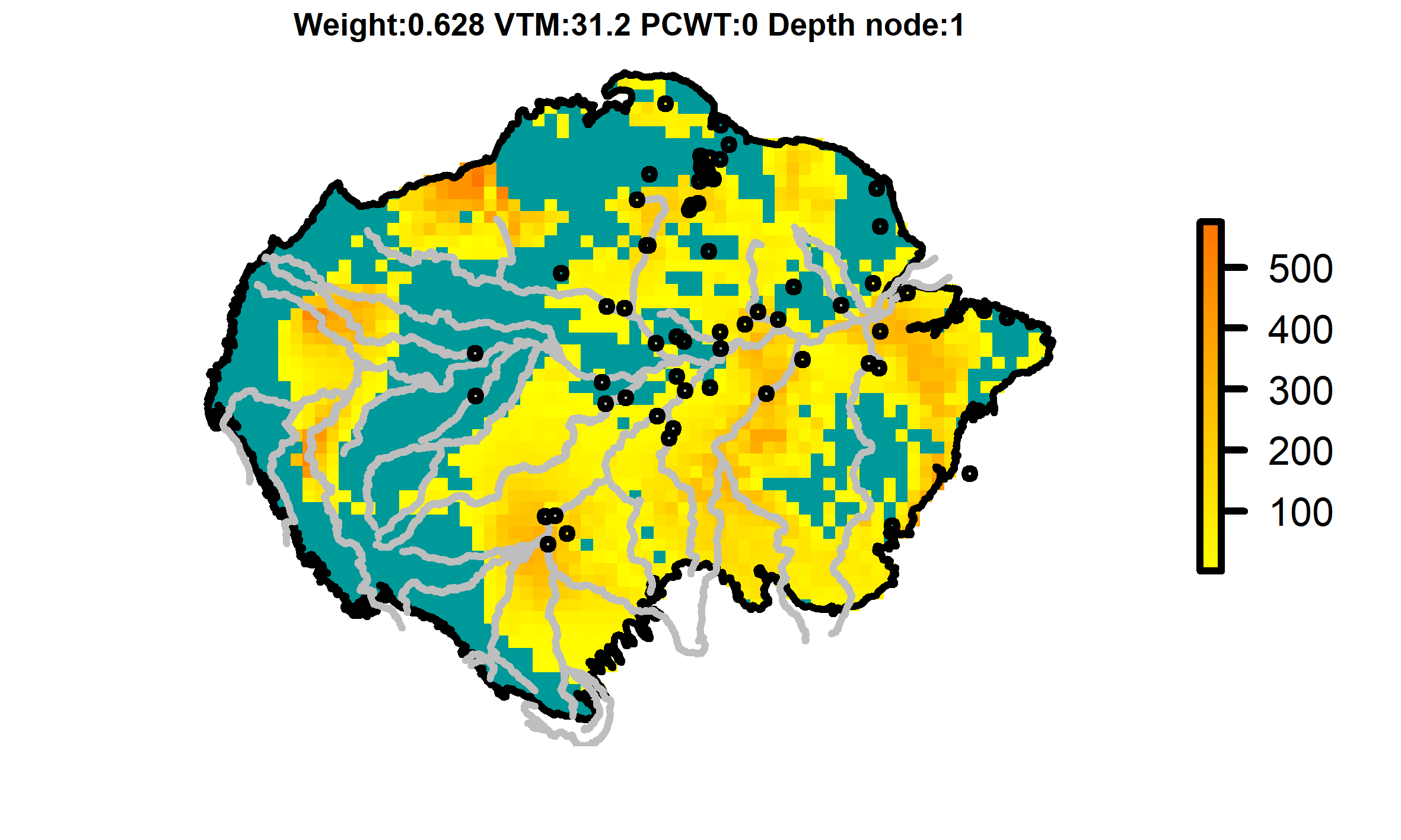

Supplement: S1 Data — (ZIP) [file pone.0286502.s002.zip › maps/map 52.png]

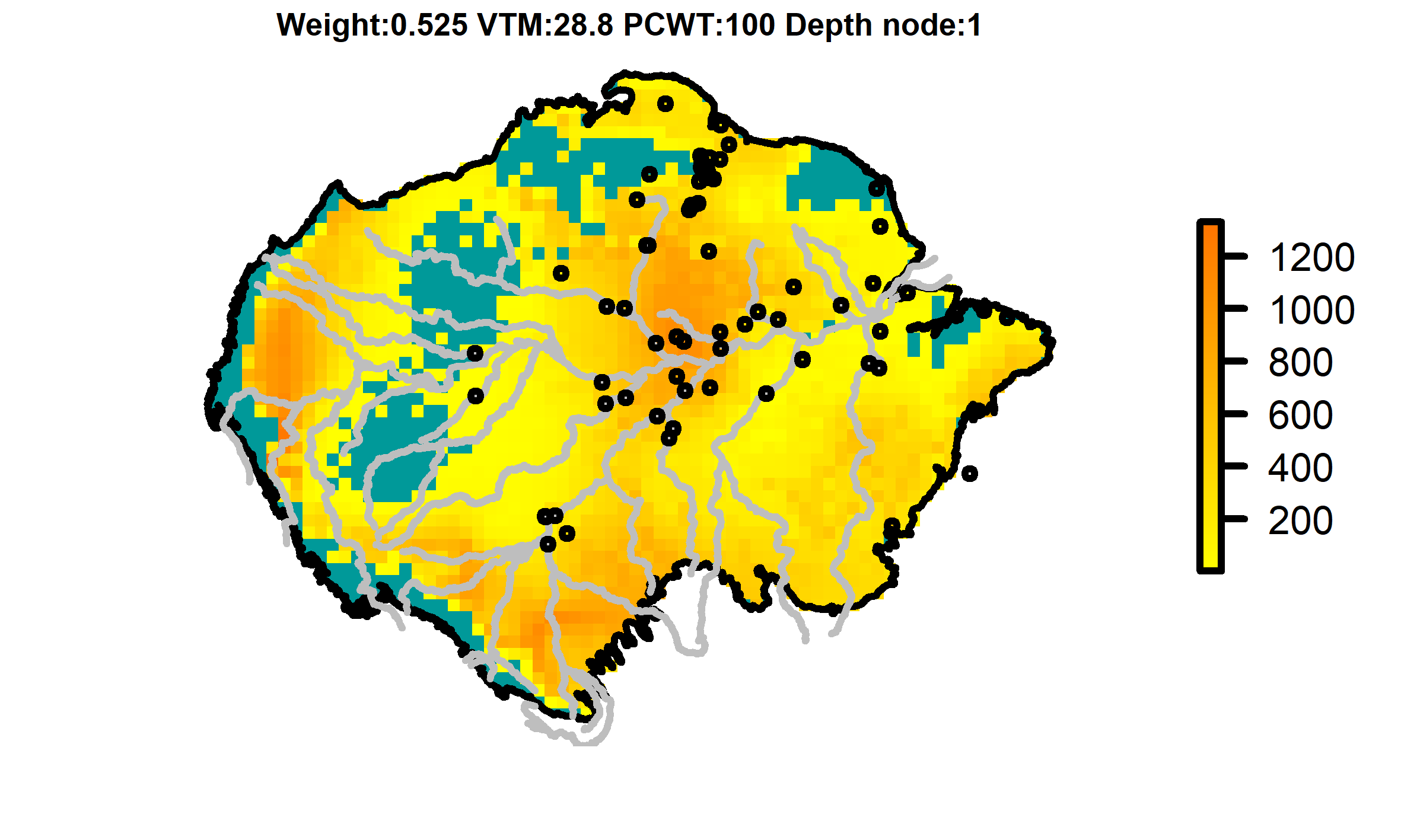

Supplement: S1 Data — (ZIP) [file pone.0286502.s002.zip › maps/map 53.png]

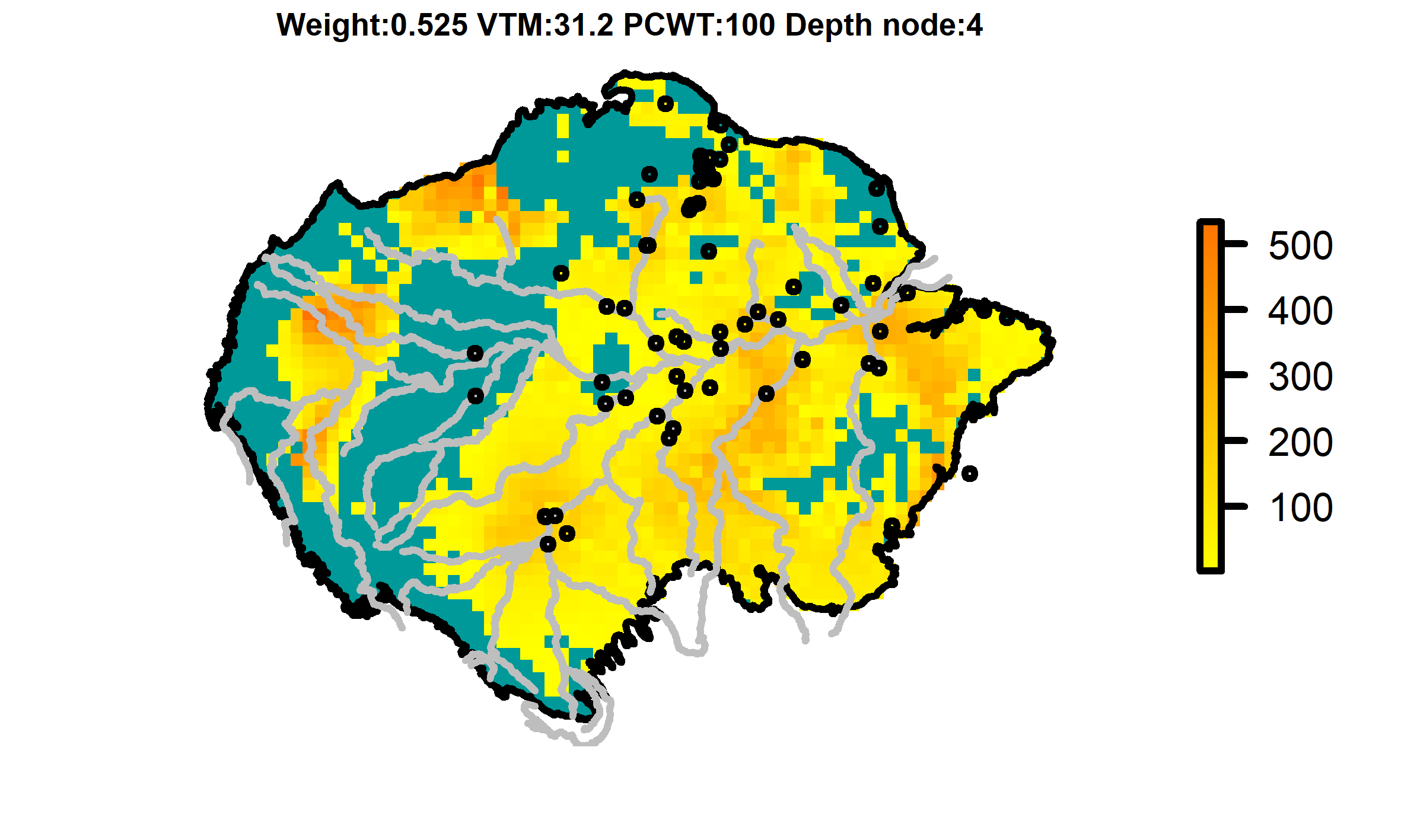

Supplement: S1 Data — (ZIP) [file pone.0286502.s002.zip › maps/map 47.png]

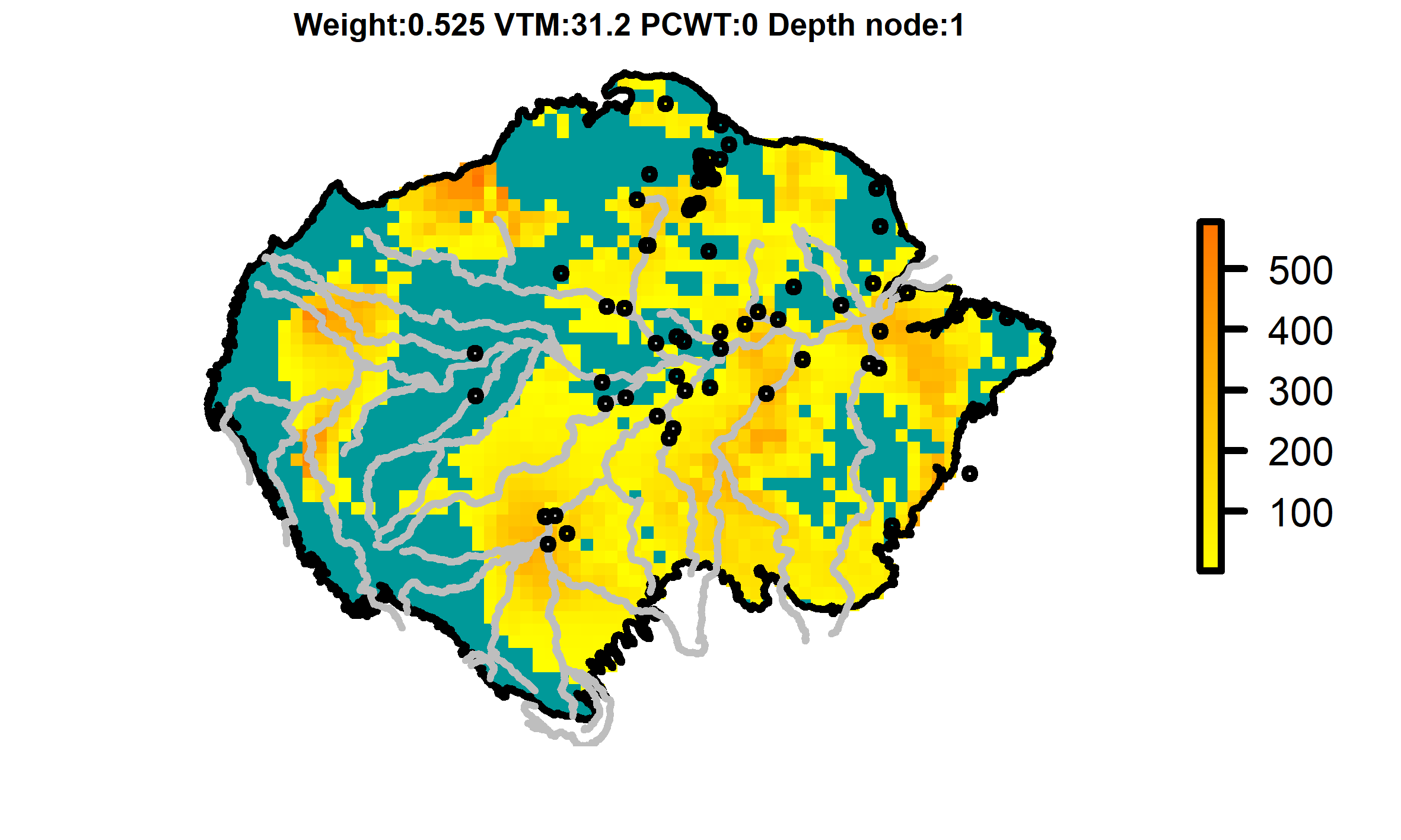

Supplement: S1 Data — (ZIP) [file pone.0286502.s002.zip › maps/map 51.png]

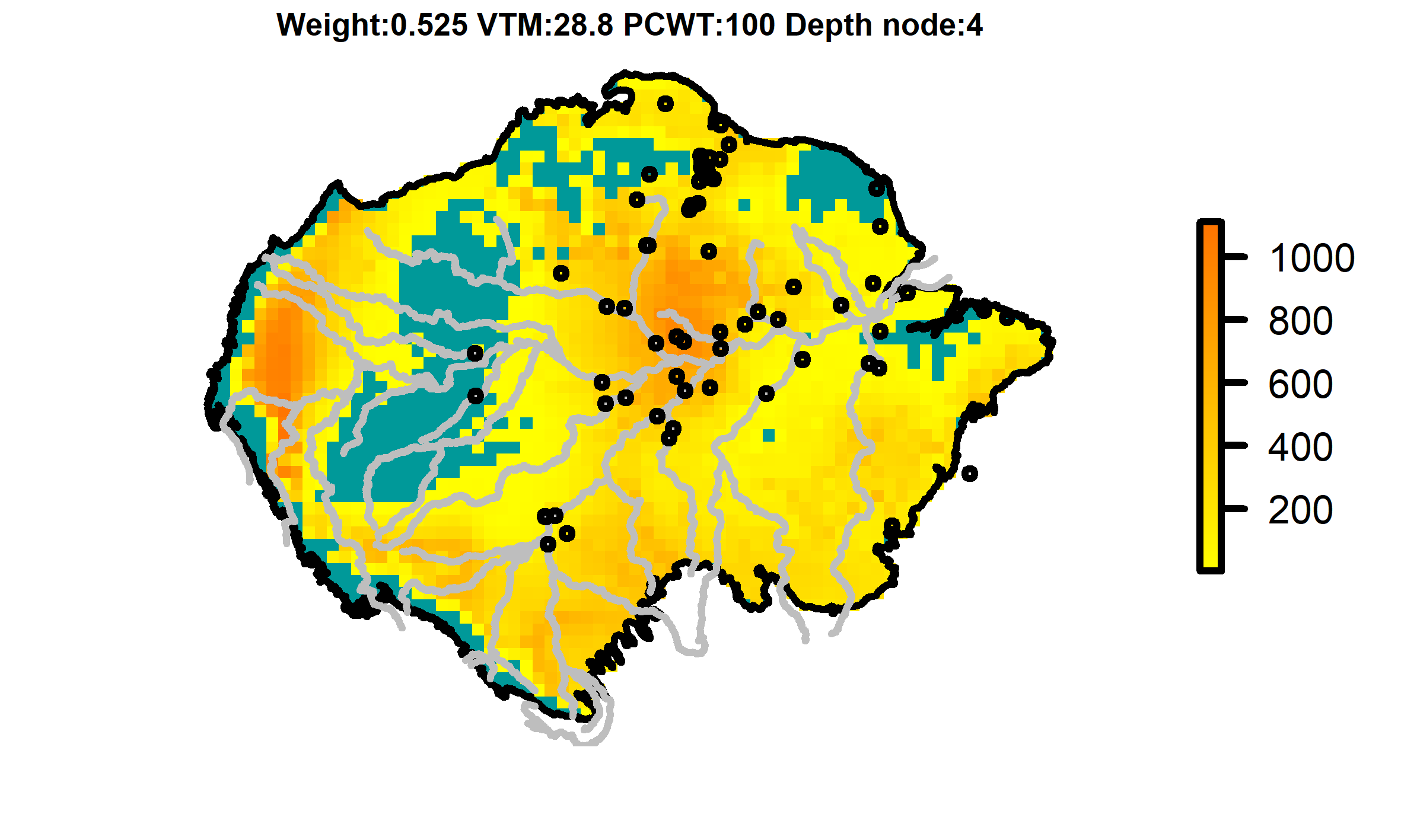

Supplement: S1 Data — (ZIP) [file pone.0286502.s002.zip › maps/map 45.png]

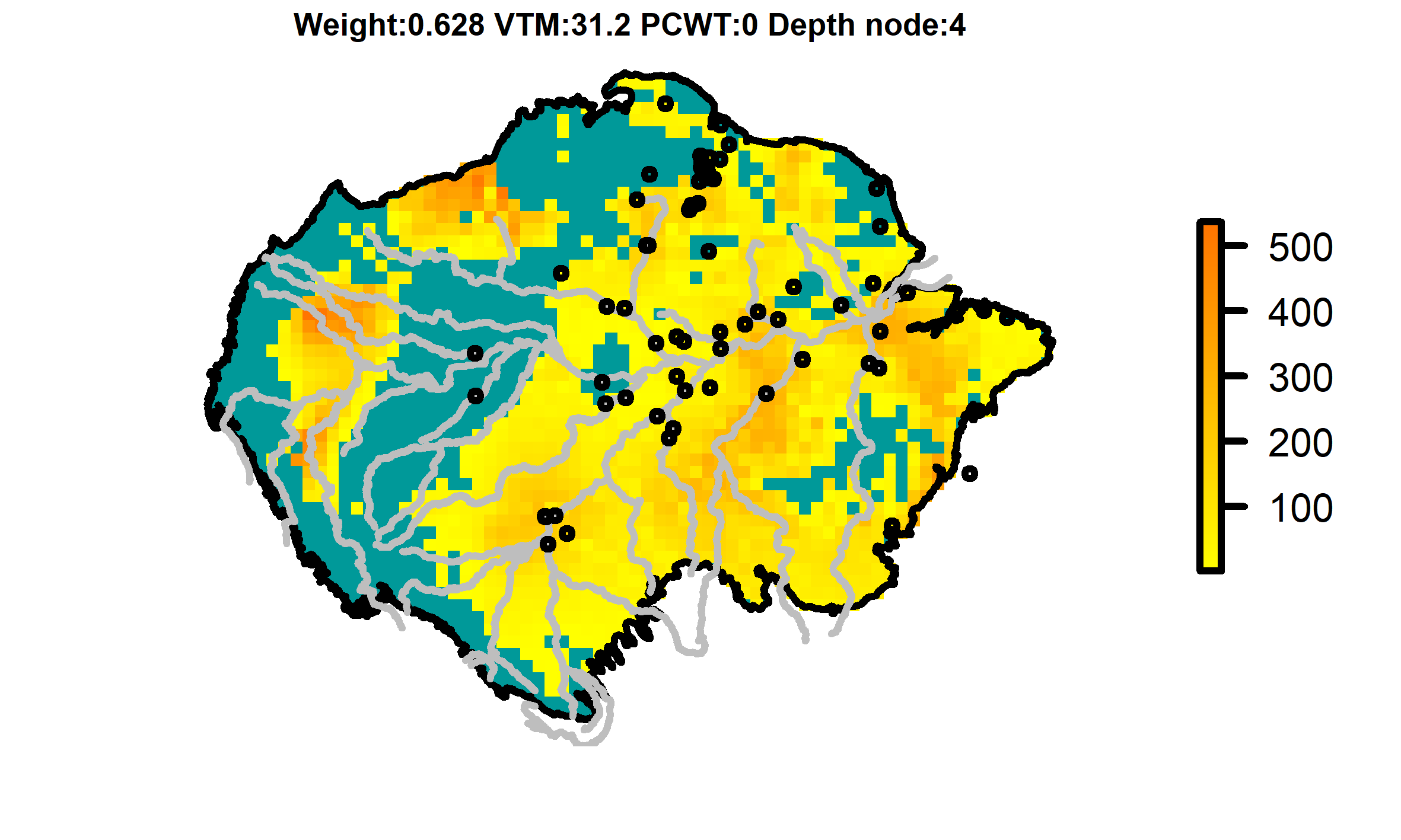

Supplement: S1 Data — (ZIP) [file pone.0286502.s002.zip › maps/map 44.png]

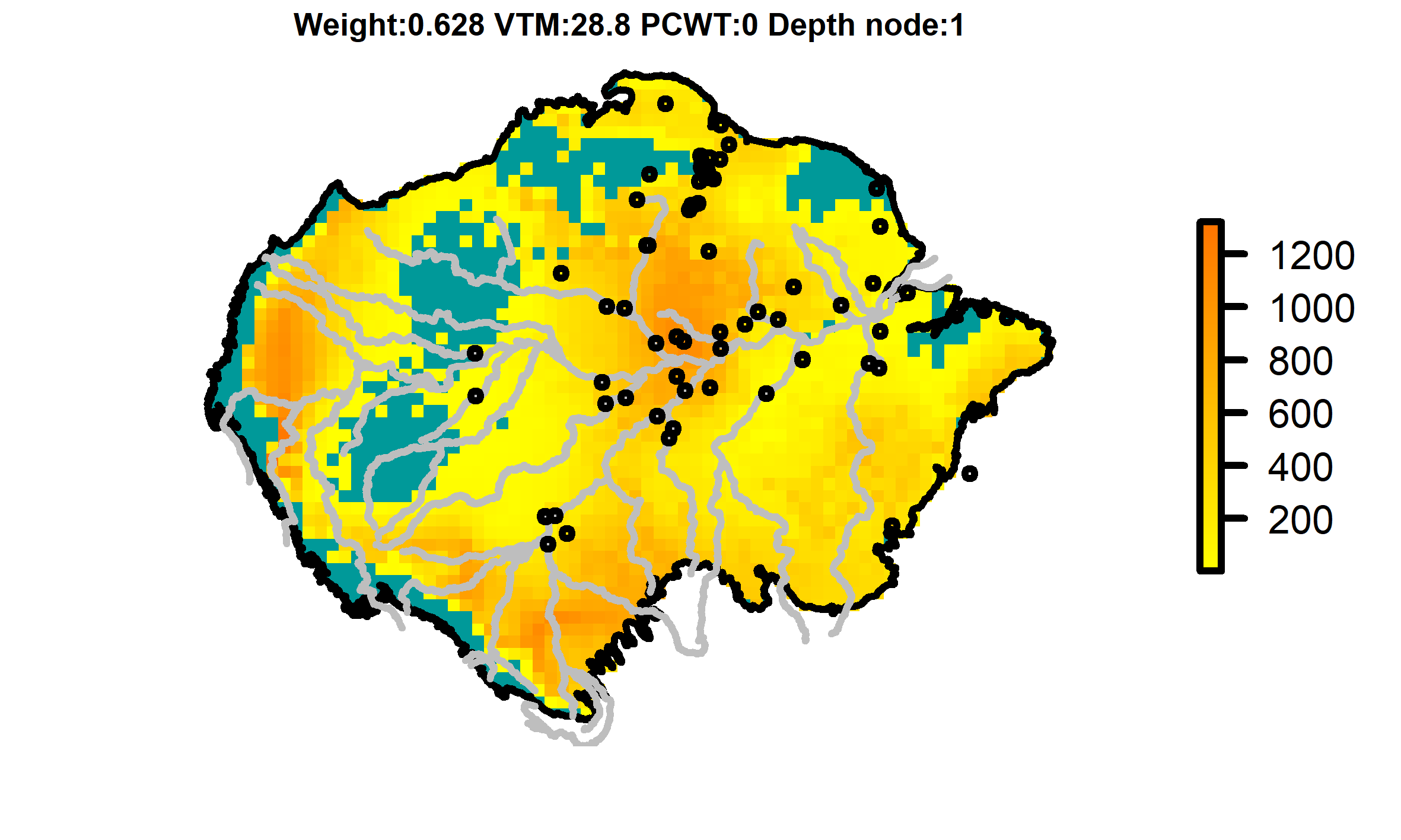

Supplement: S1 Data — (ZIP) [file pone.0286502.s002.zip › maps/map 50.png]

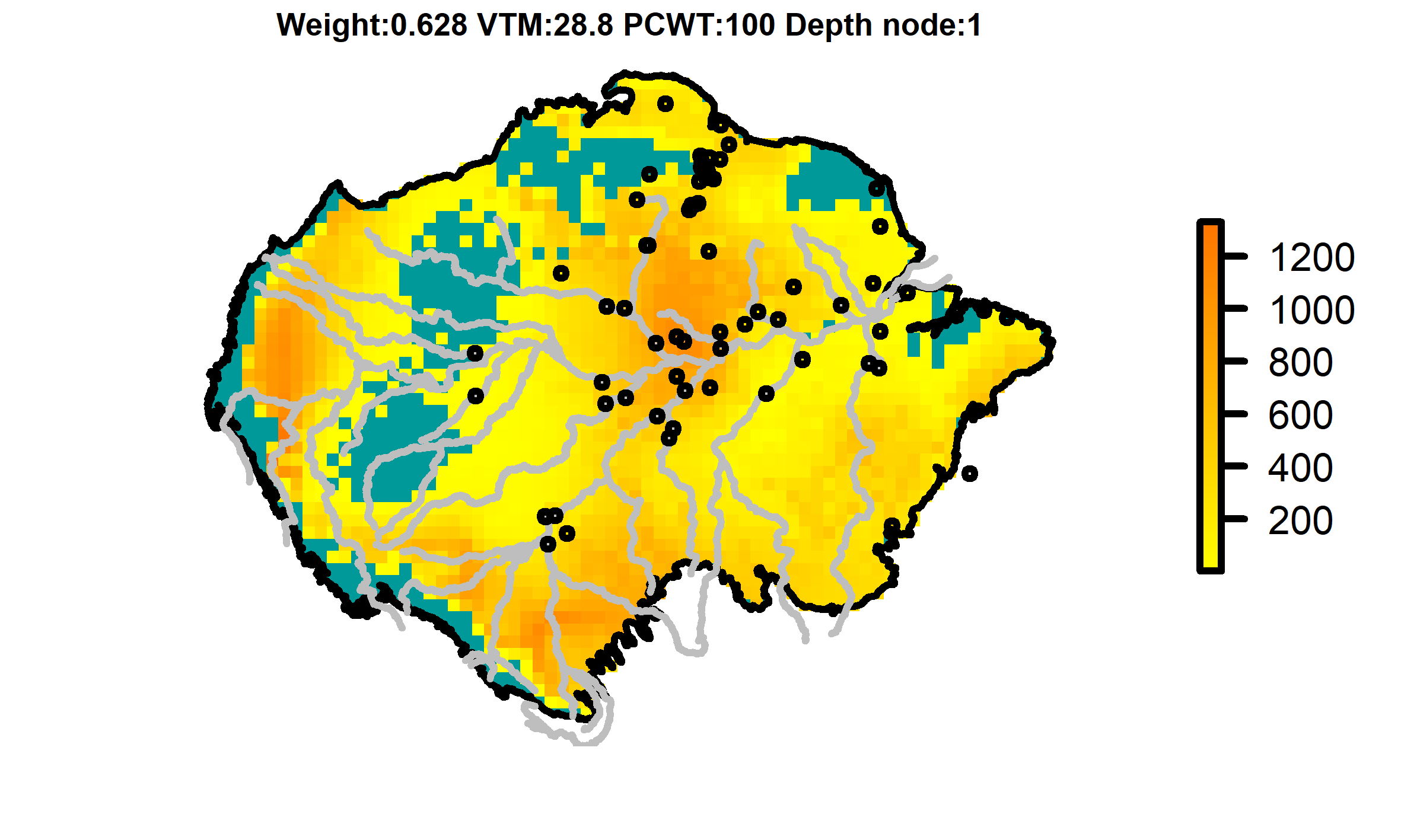

Supplement: S1 Data — (ZIP) [file pone.0286502.s002.zip › maps/map 54.png]

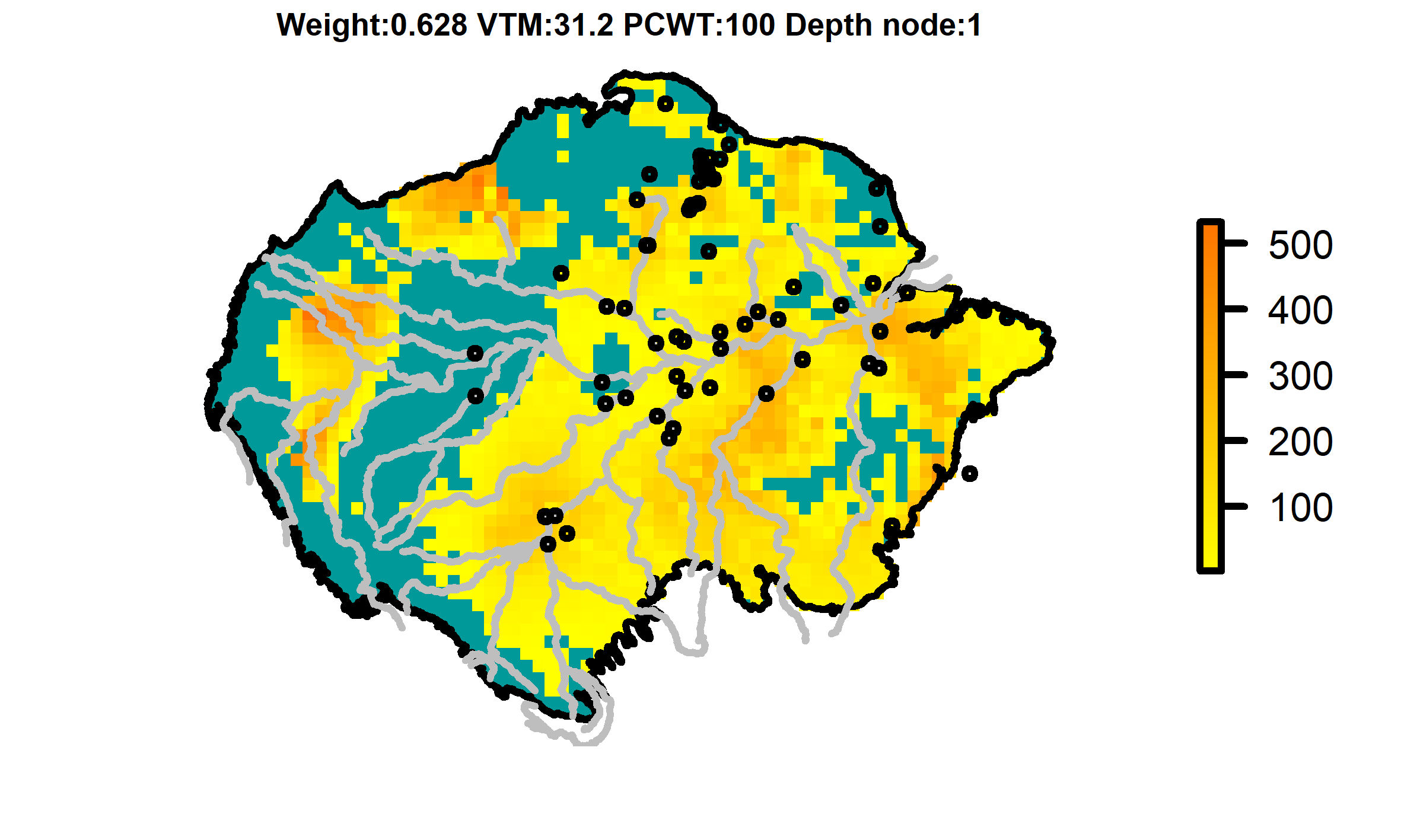

Supplement: S1 Data — (ZIP) [file pone.0286502.s002.zip › maps/map 40.png]

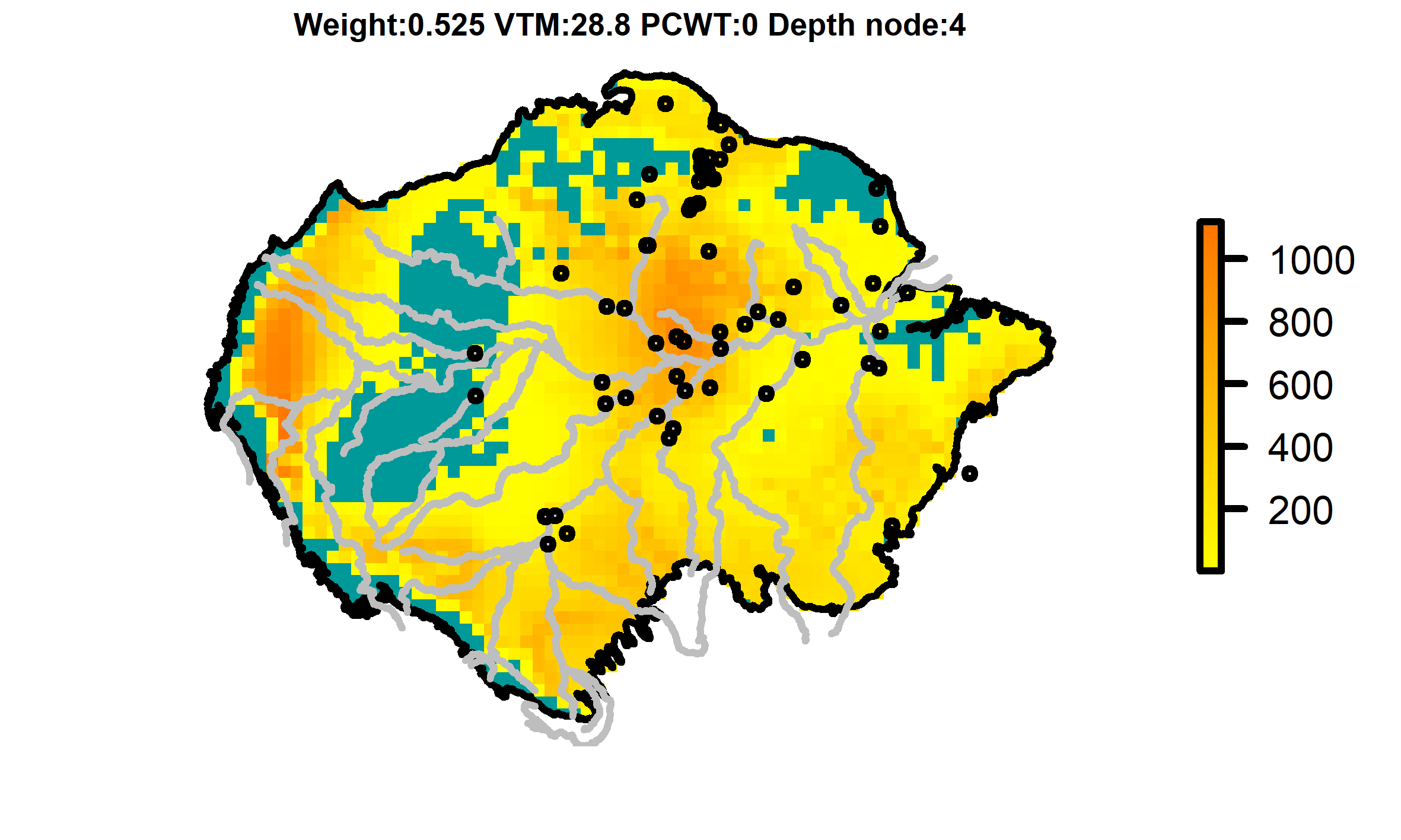

Supplement: S1 Data — (ZIP) [file pone.0286502.s002.zip › maps/map 41.png]

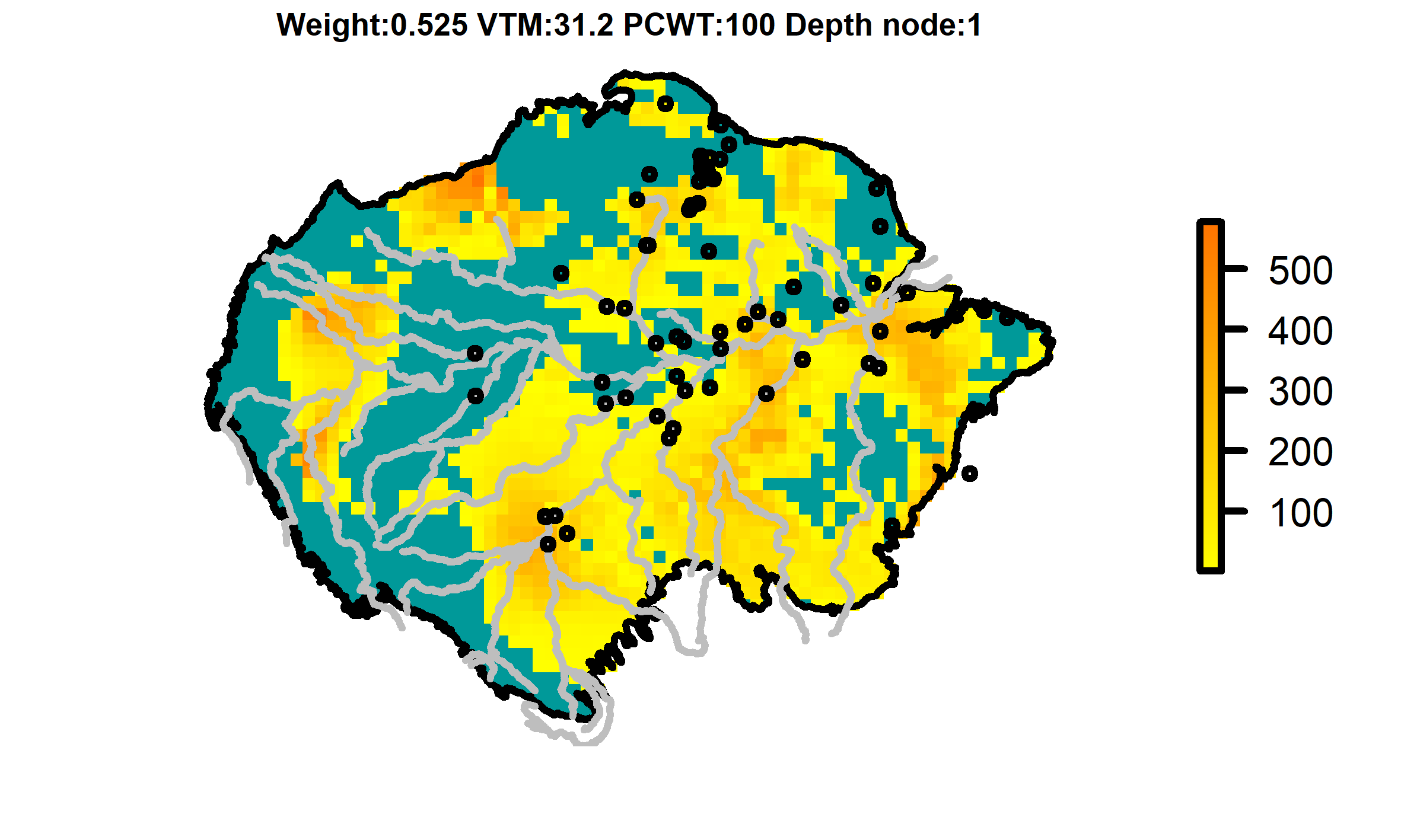

Supplement: S1 Data — (ZIP) [file pone.0286502.s002.zip › maps/map 55.png]

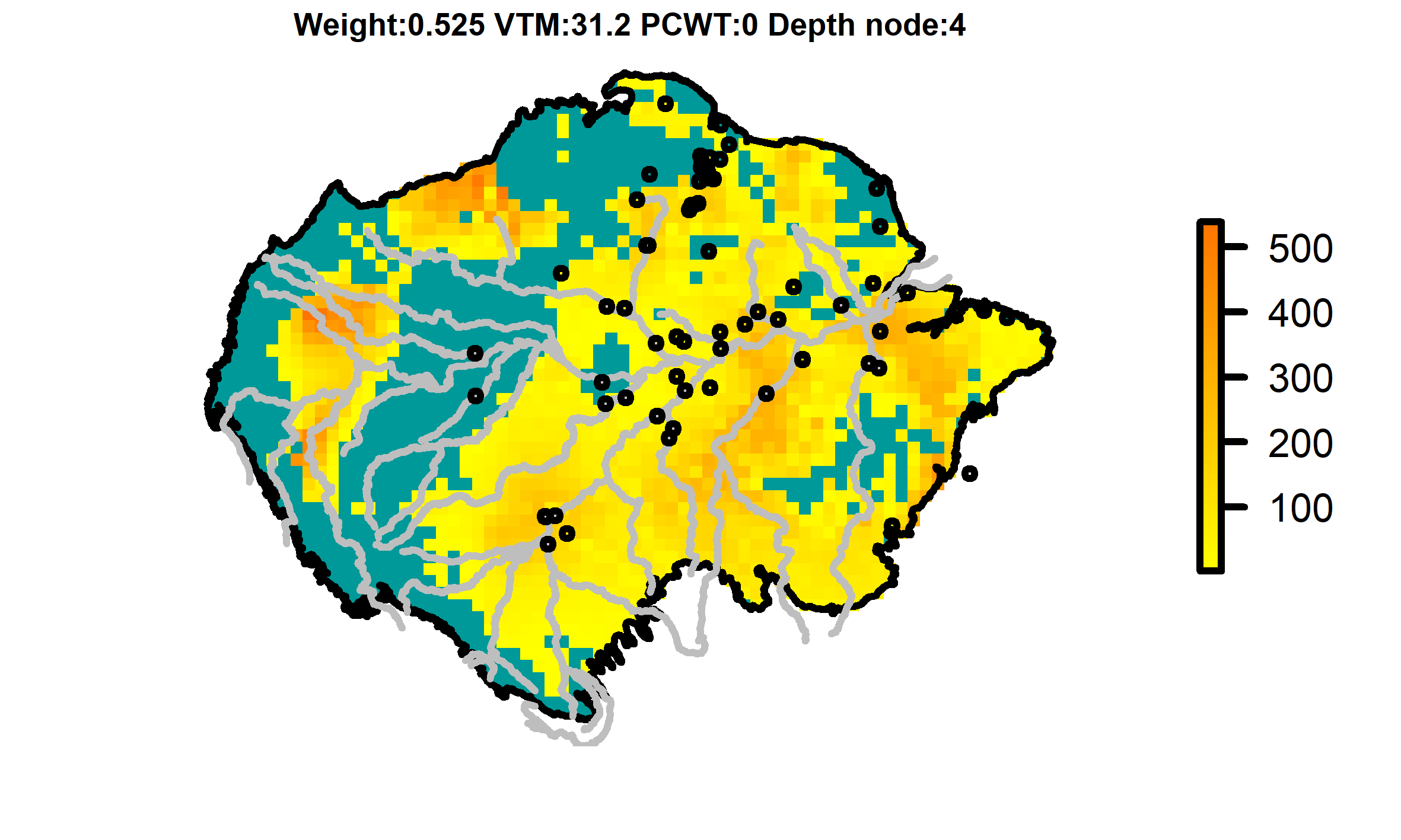

Supplement: S1 Data — (ZIP) [file pone.0286502.s002.zip › maps/map 43.png]

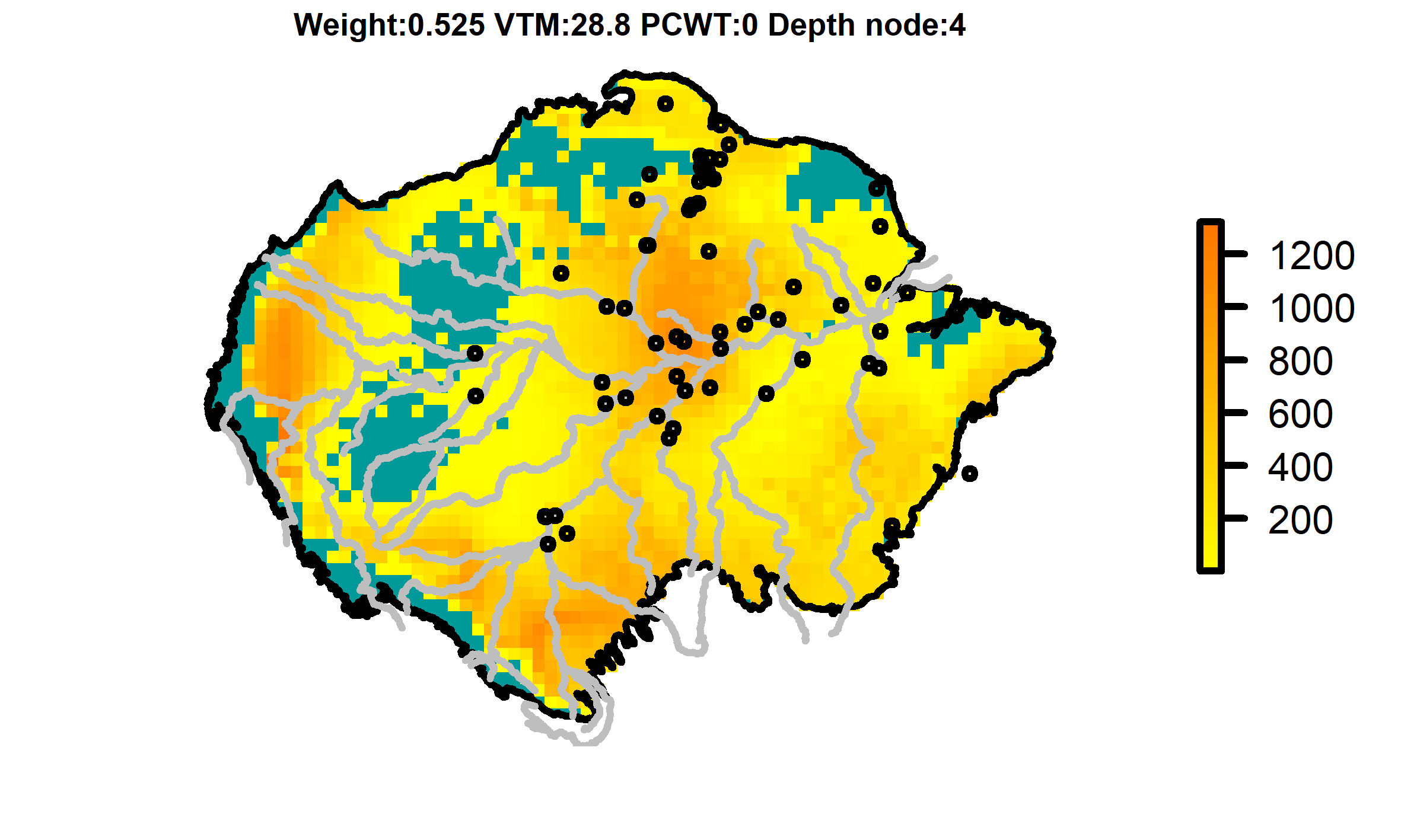

Supplement: S1 Data — (ZIP) [file pone.0286502.s002.zip › maps/map 57.png]

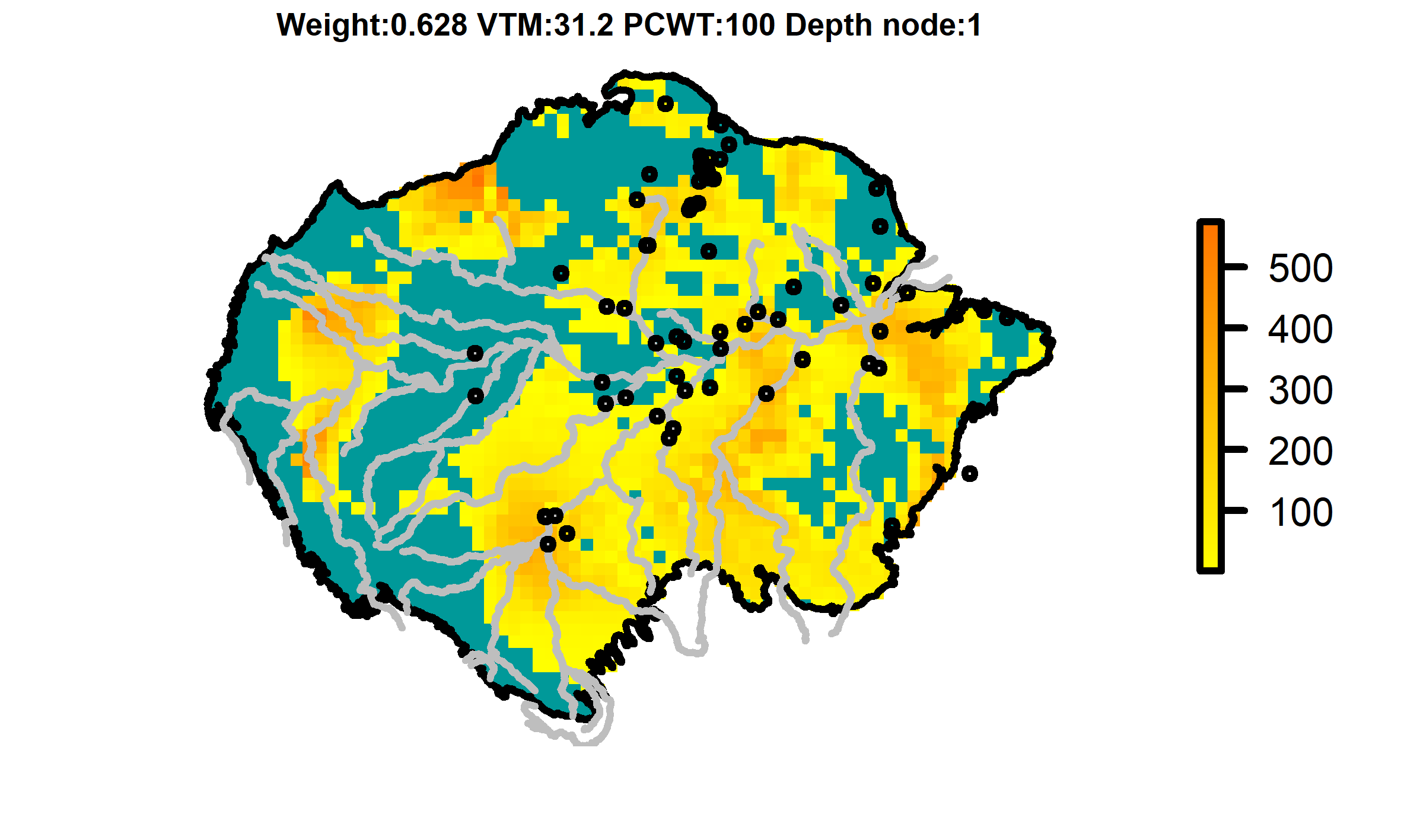

Supplement: S1 Data — (ZIP) [file pone.0286502.s002.zip › maps/map 56.png]

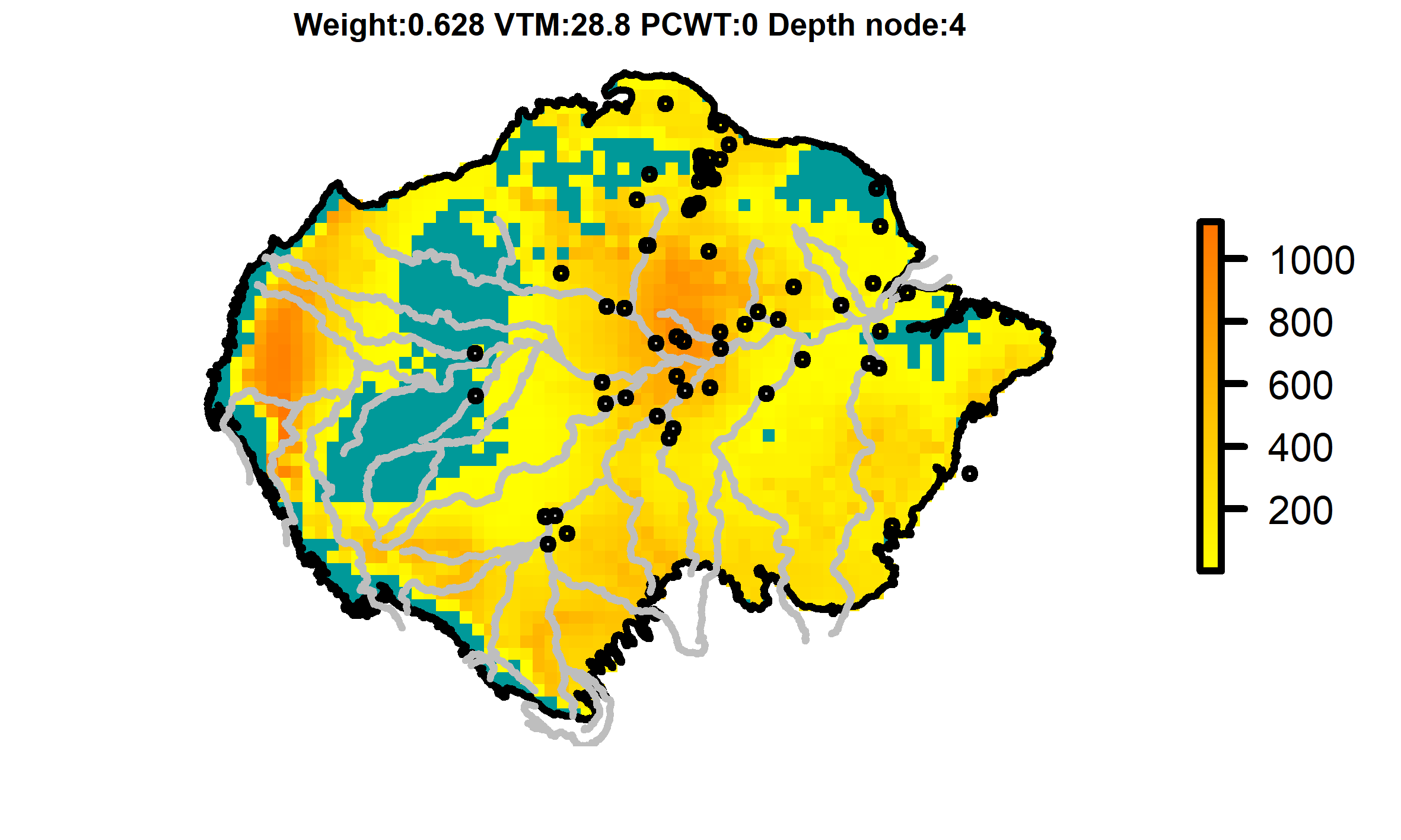

Supplement: S1 Data — (ZIP) [file pone.0286502.s002.zip › maps/map 42.png]

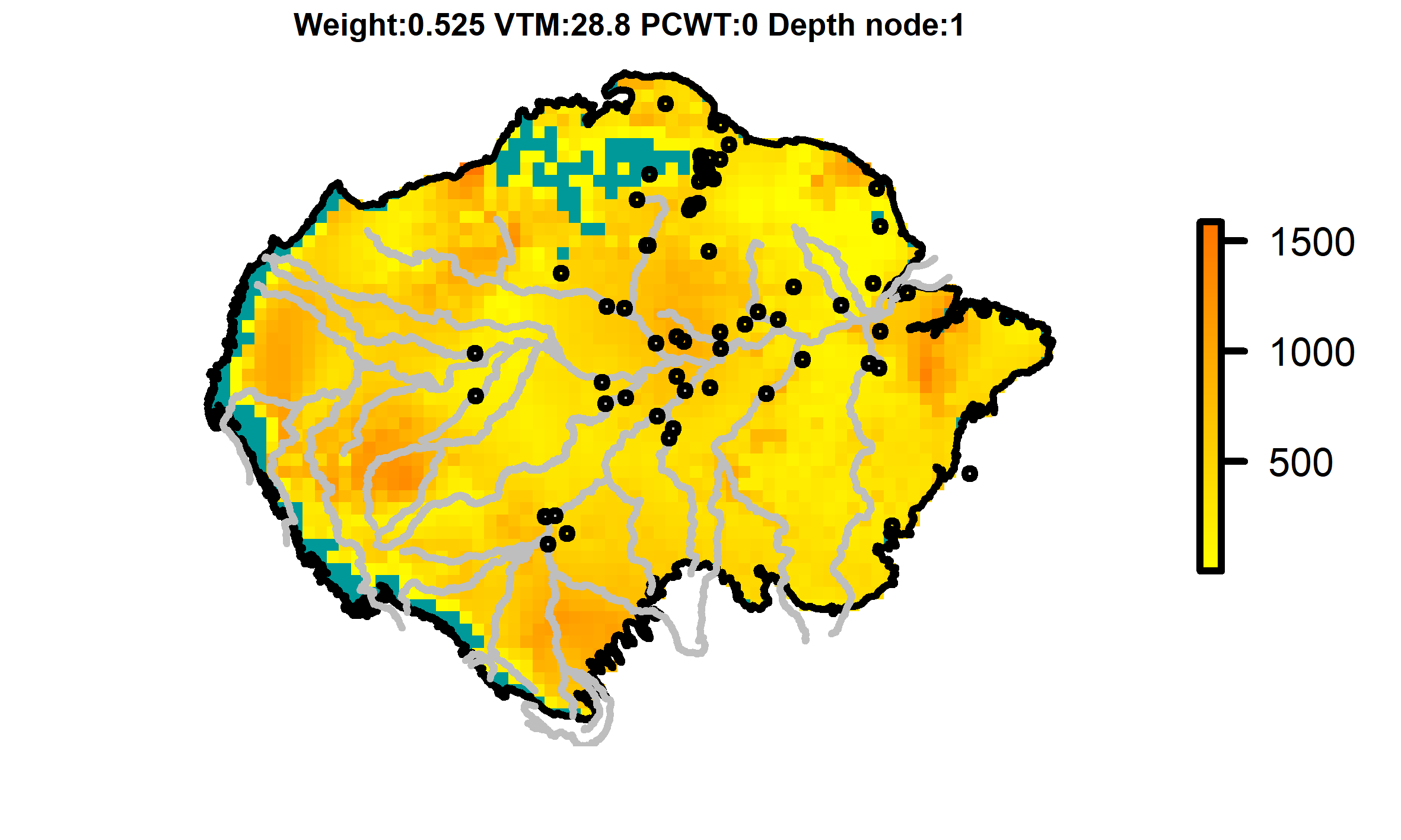

Supplement: S1 Data — (ZIP) [file pone.0286502.s002.zip › maps/map 1.png]

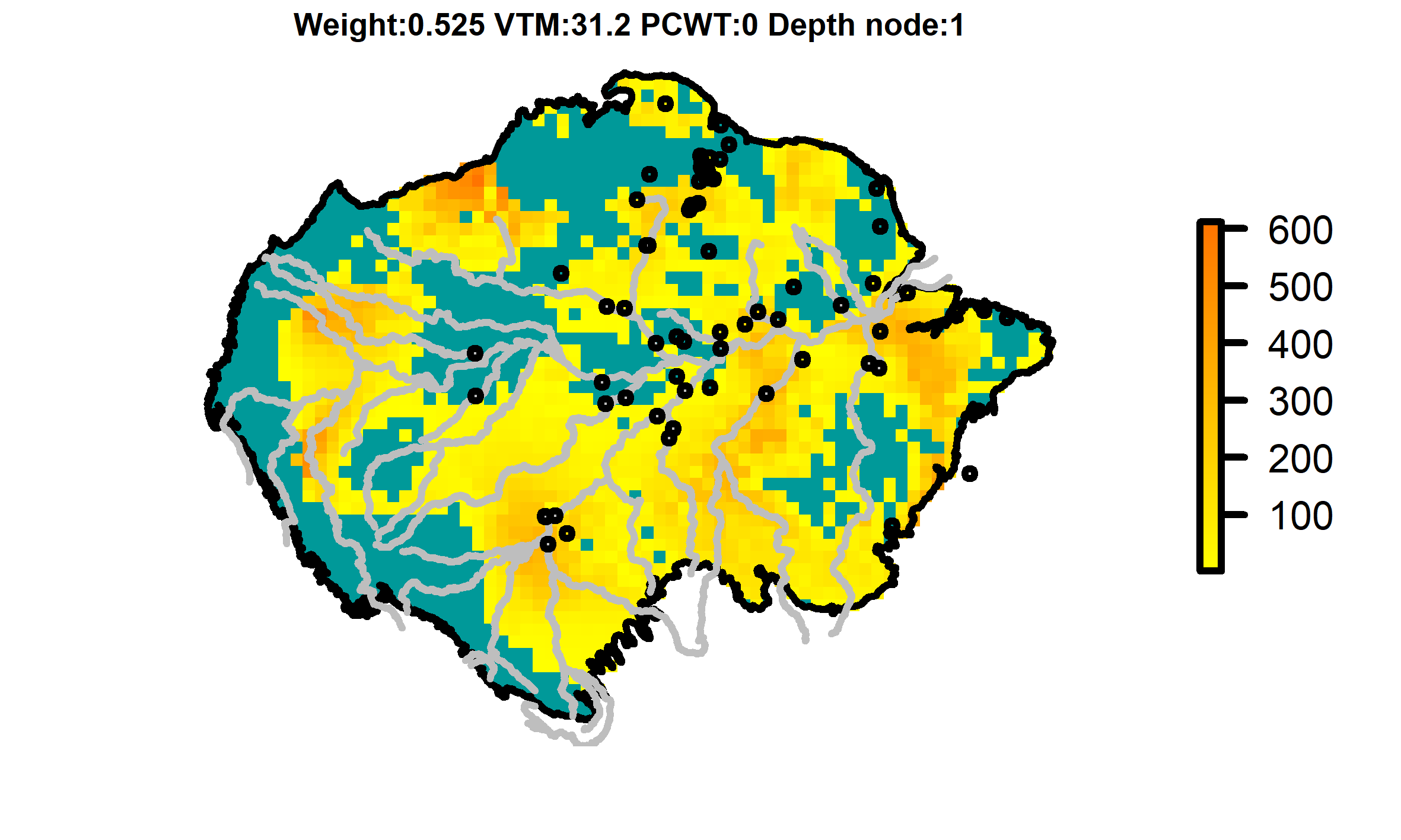

Supplement: S1 Data — (ZIP) [file pone.0286502.s002.zip › maps/map 19.png]

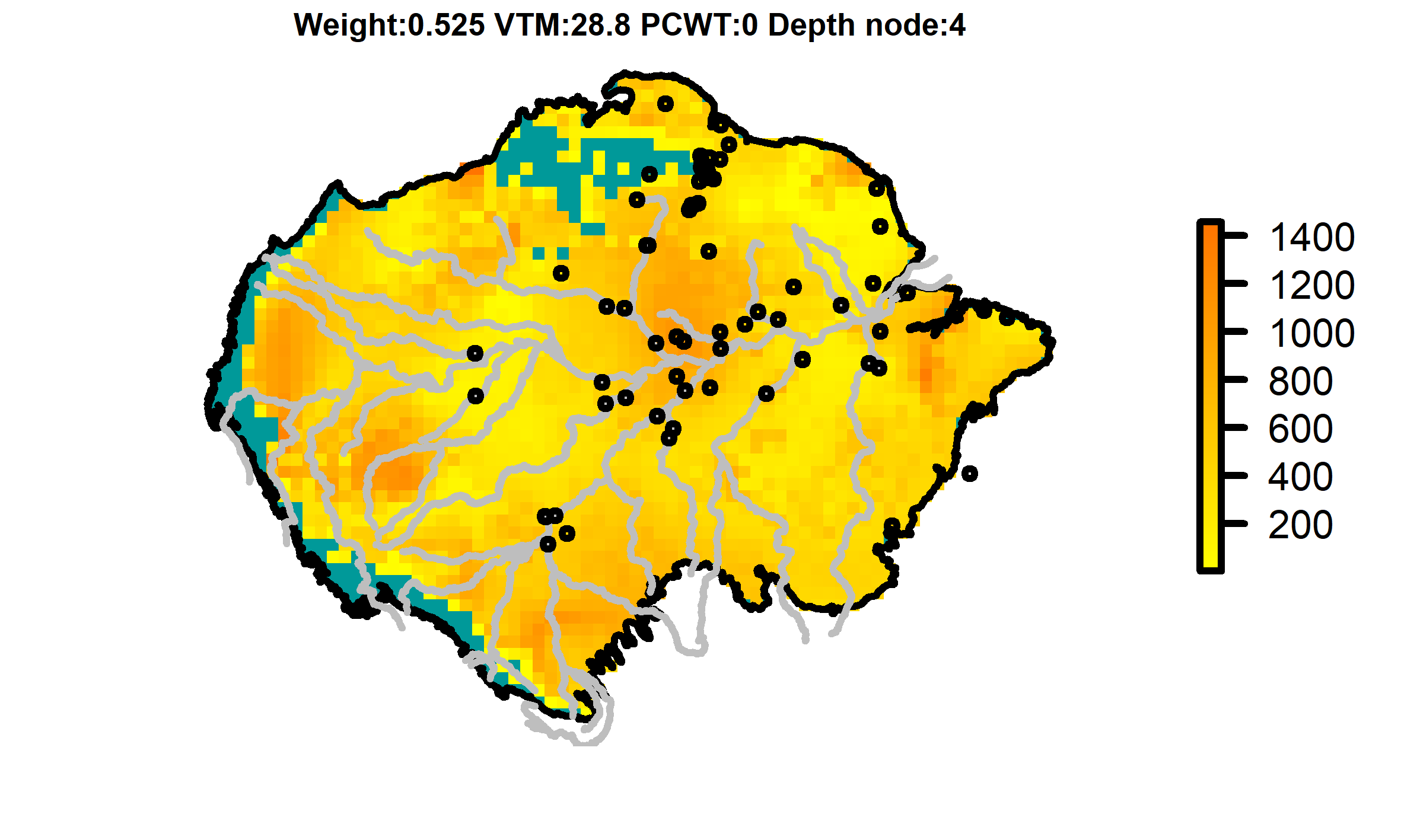

Supplement: S1 Data — (ZIP) [file pone.0286502.s002.zip › maps/map 25.png]

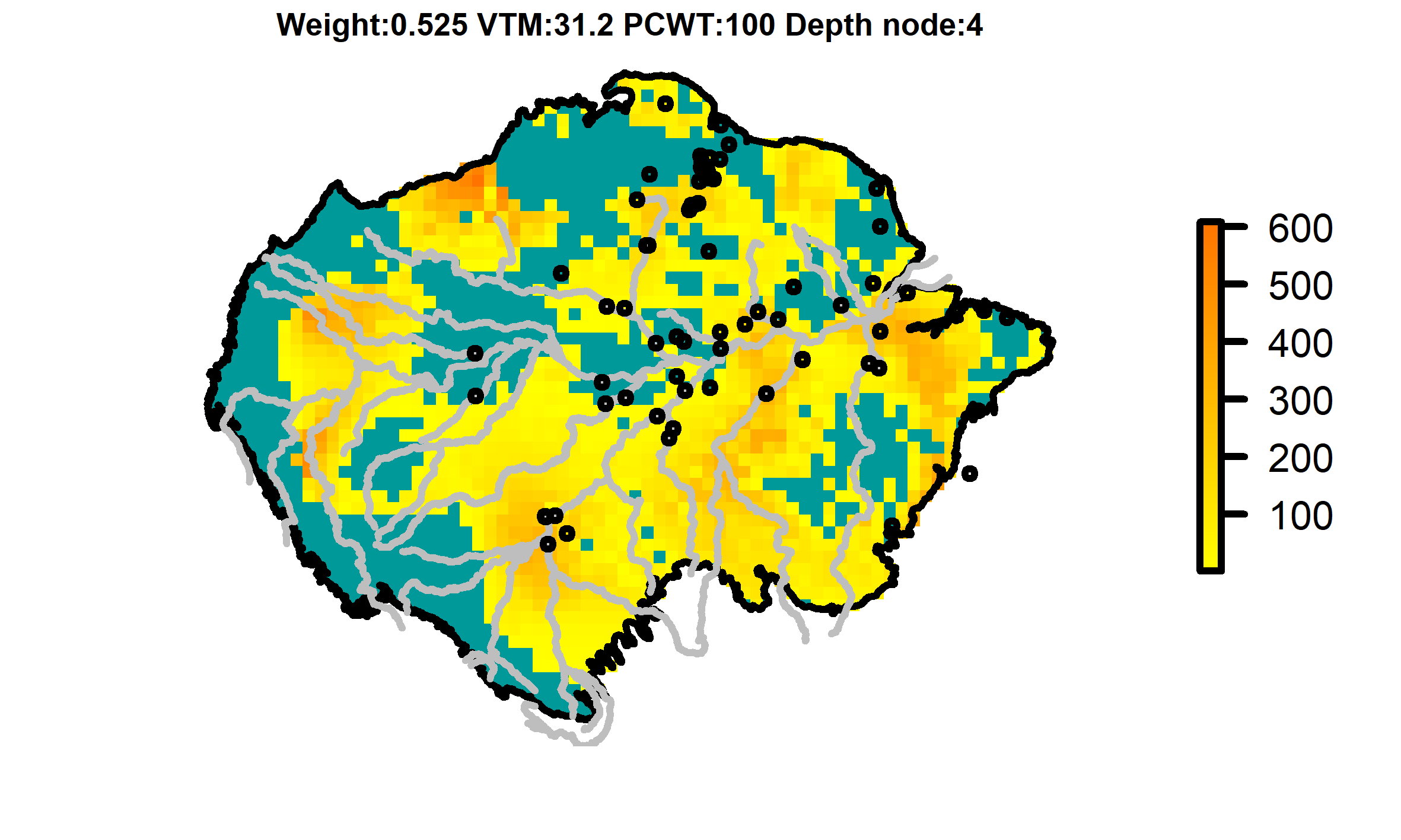

Supplement: S1 Data — (ZIP) [file pone.0286502.s002.zip › maps/map 31.png]

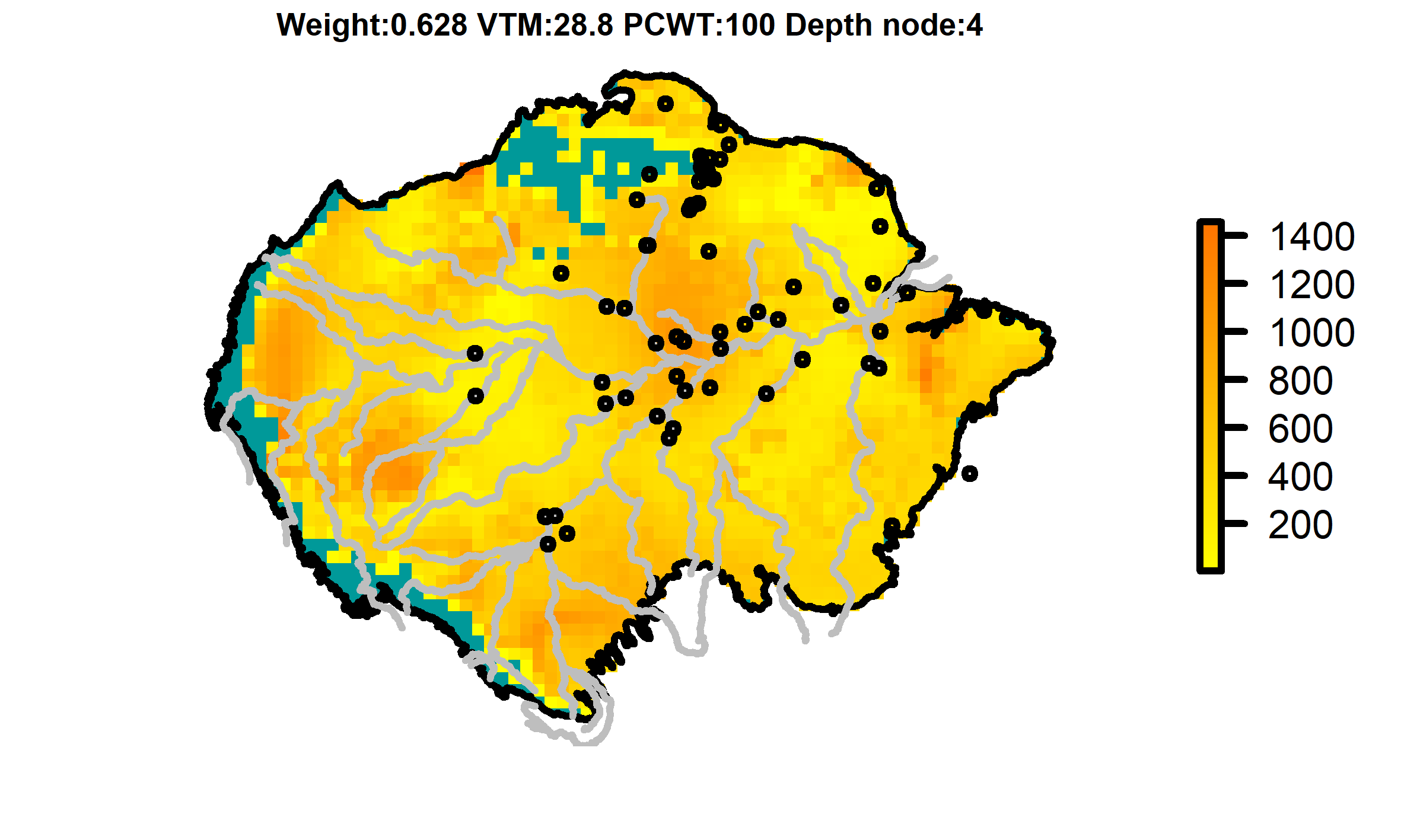

Supplement: S1 Data — (ZIP) [file pone.0286502.s002.zip › maps/map 30.png]

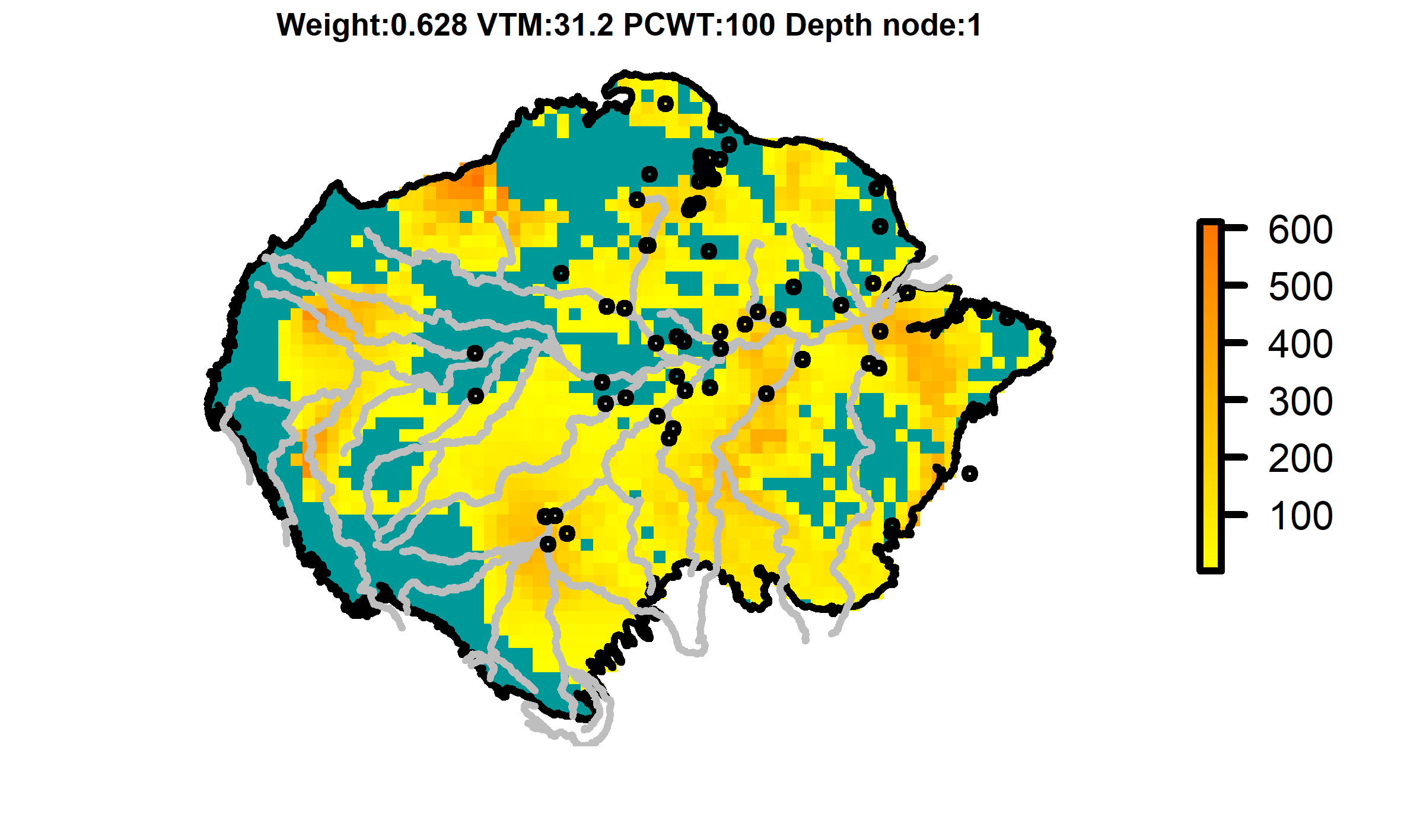

Supplement: S1 Data — (ZIP) [file pone.0286502.s002.zip › maps/map 24.png]

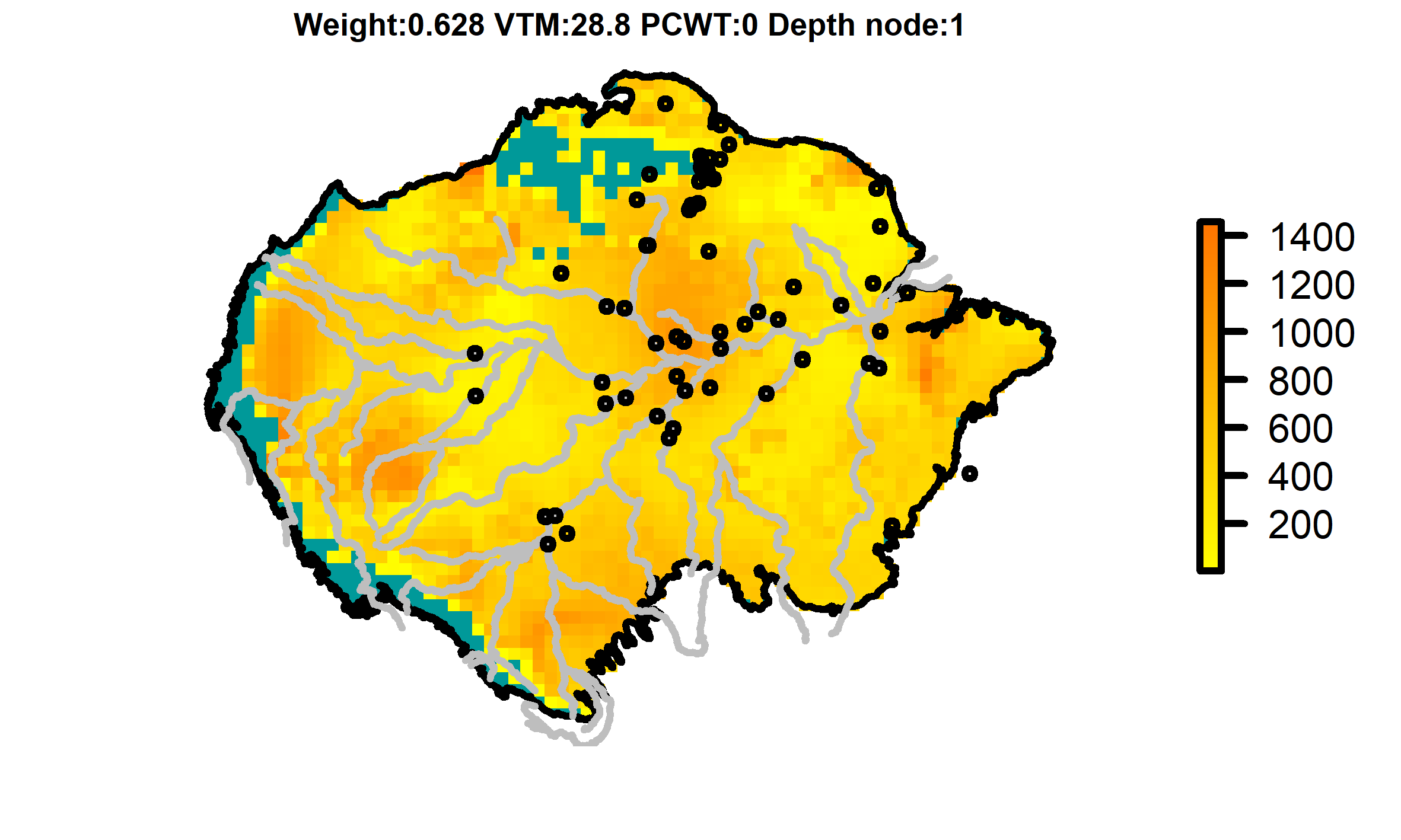

Supplement: S1 Data — (ZIP) [file pone.0286502.s002.zip › maps/map 18.png]

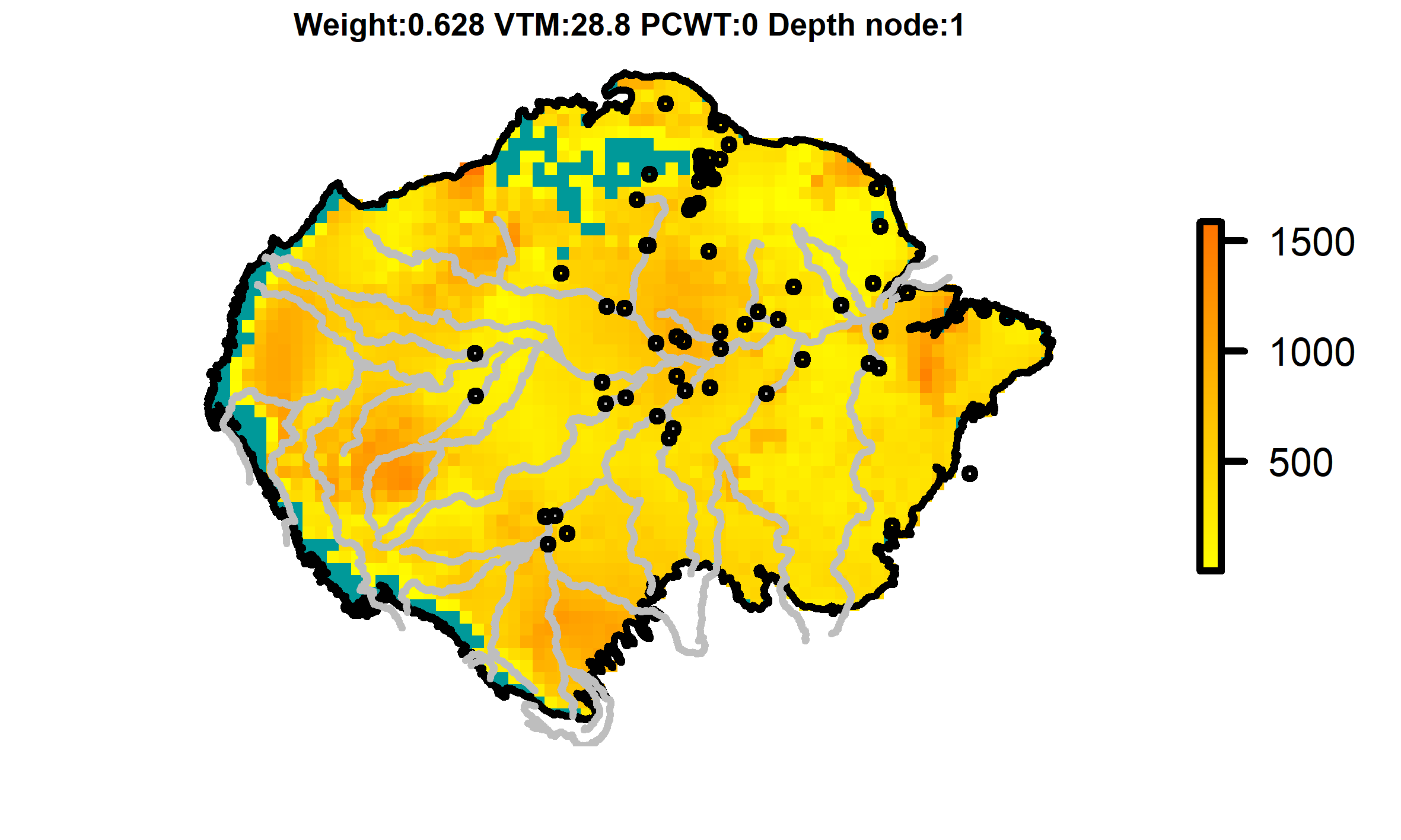

Supplement: S1 Data — (ZIP) [file pone.0286502.s002.zip › maps/map 2.png]

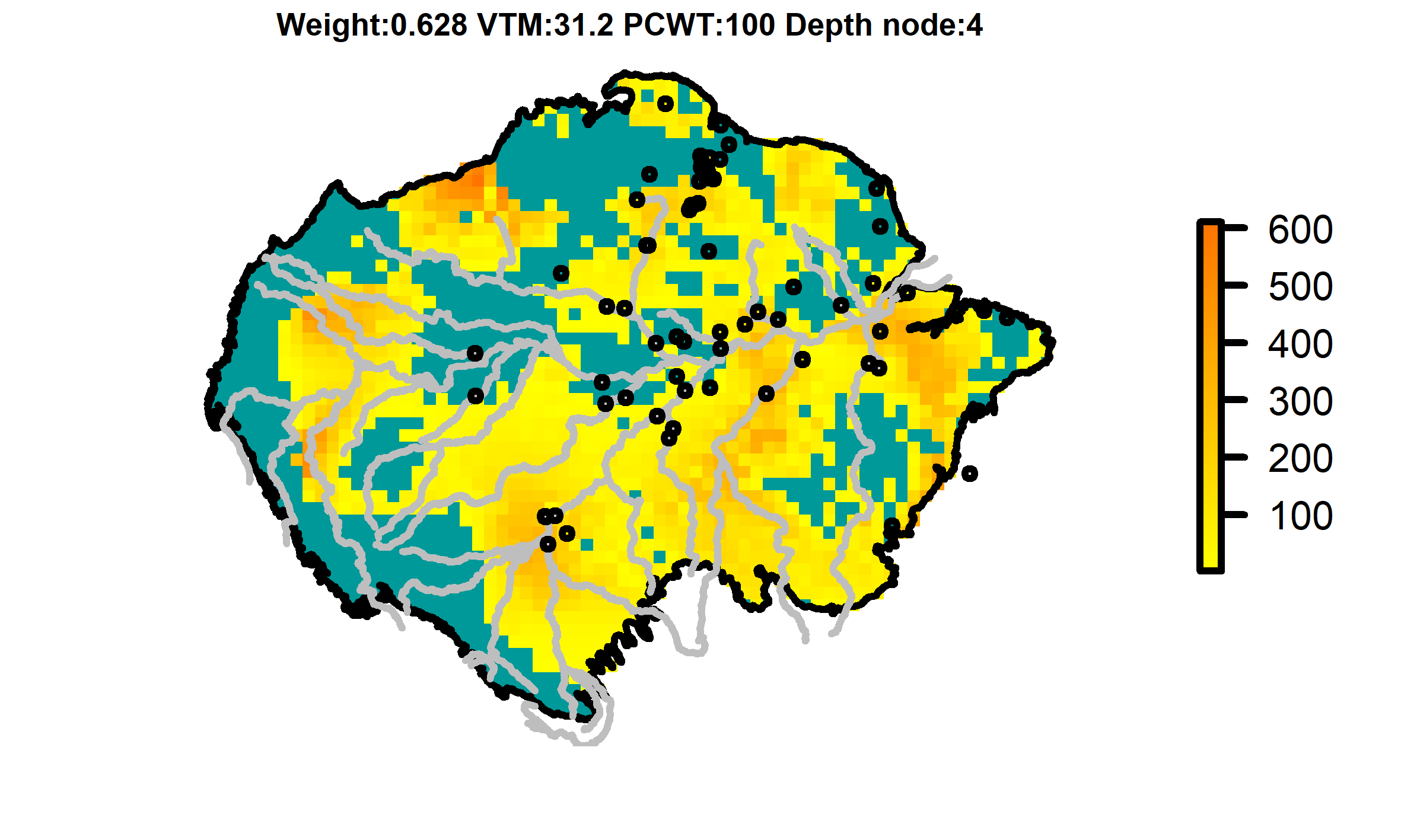

Supplement: S1 Data — (ZIP) [file pone.0286502.s002.zip › maps/map 32.png]

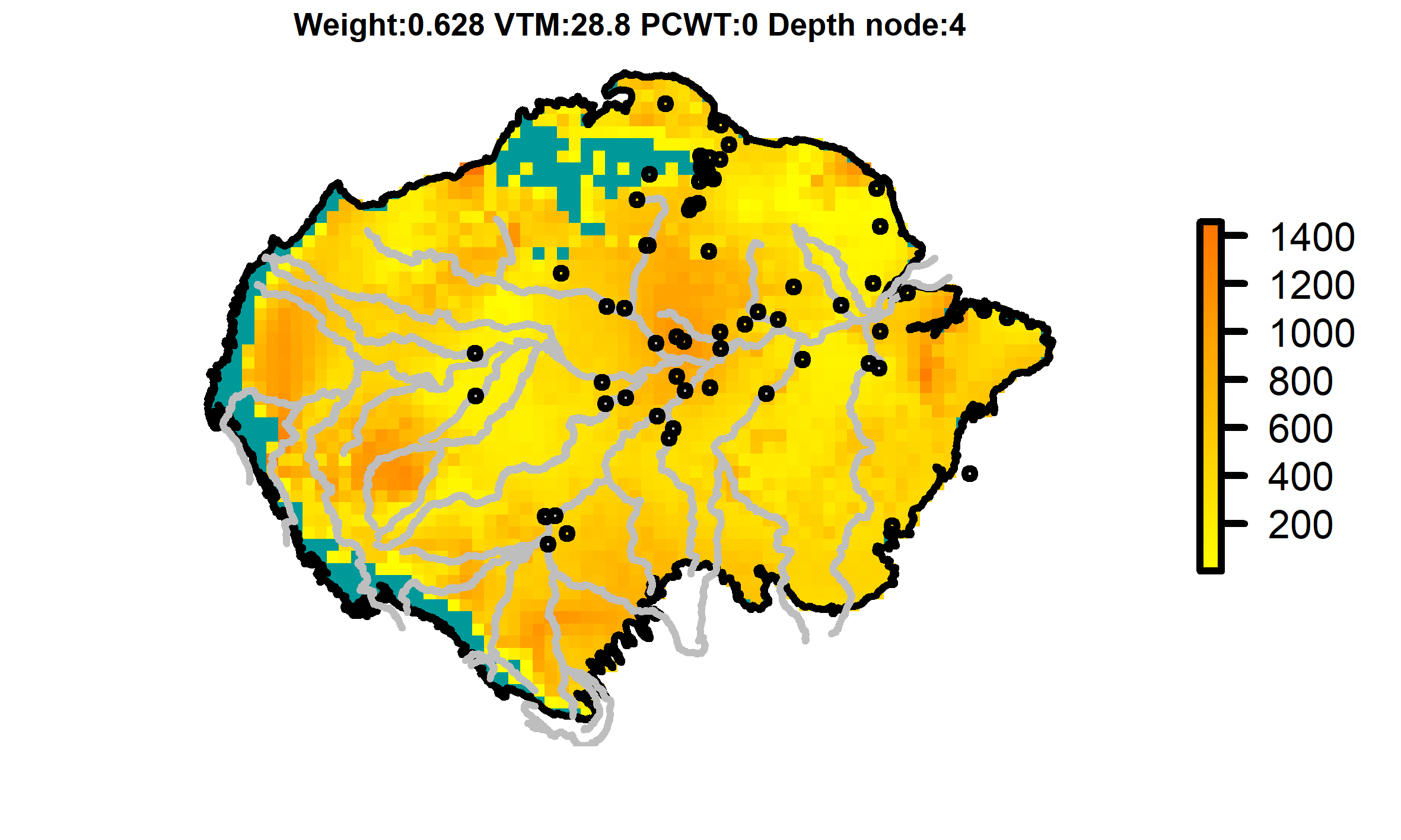

Supplement: S1 Data — (ZIP) [file pone.0286502.s002.zip › maps/map 26.png]

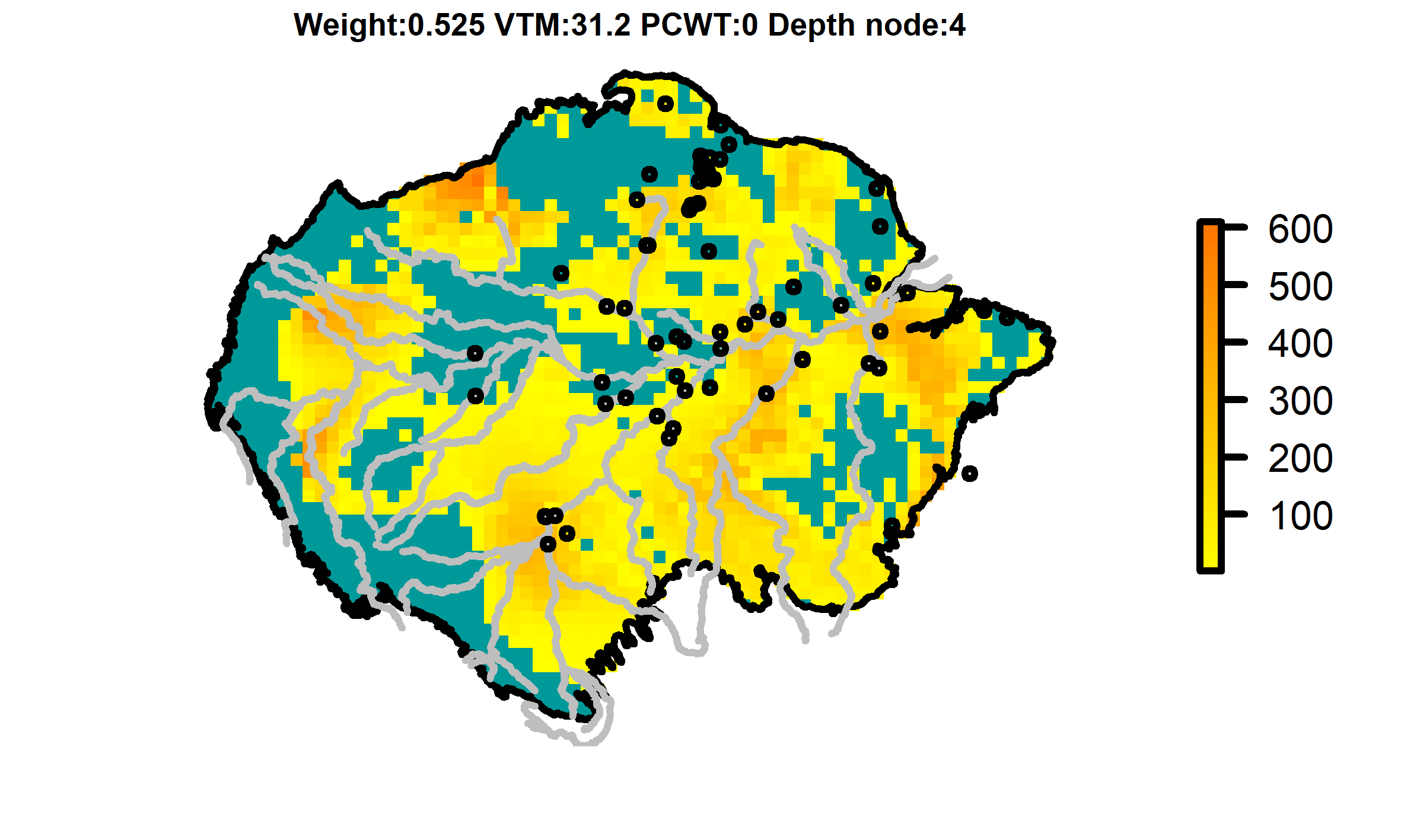

Supplement: S1 Data — (ZIP) [file pone.0286502.s002.zip › maps/map 27.png]

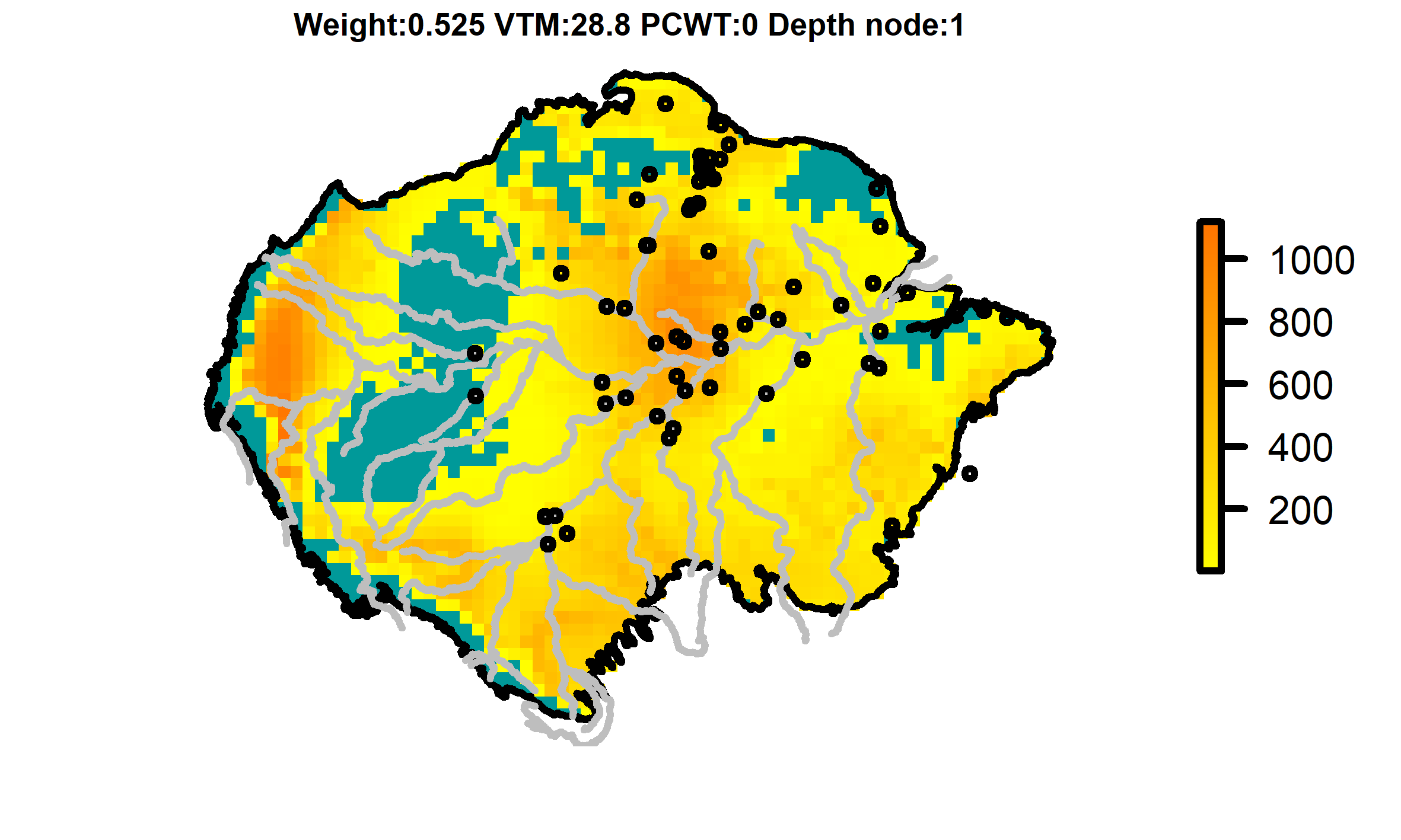

Supplement: S1 Data — (ZIP) [file pone.0286502.s002.zip › maps/map 33.png]

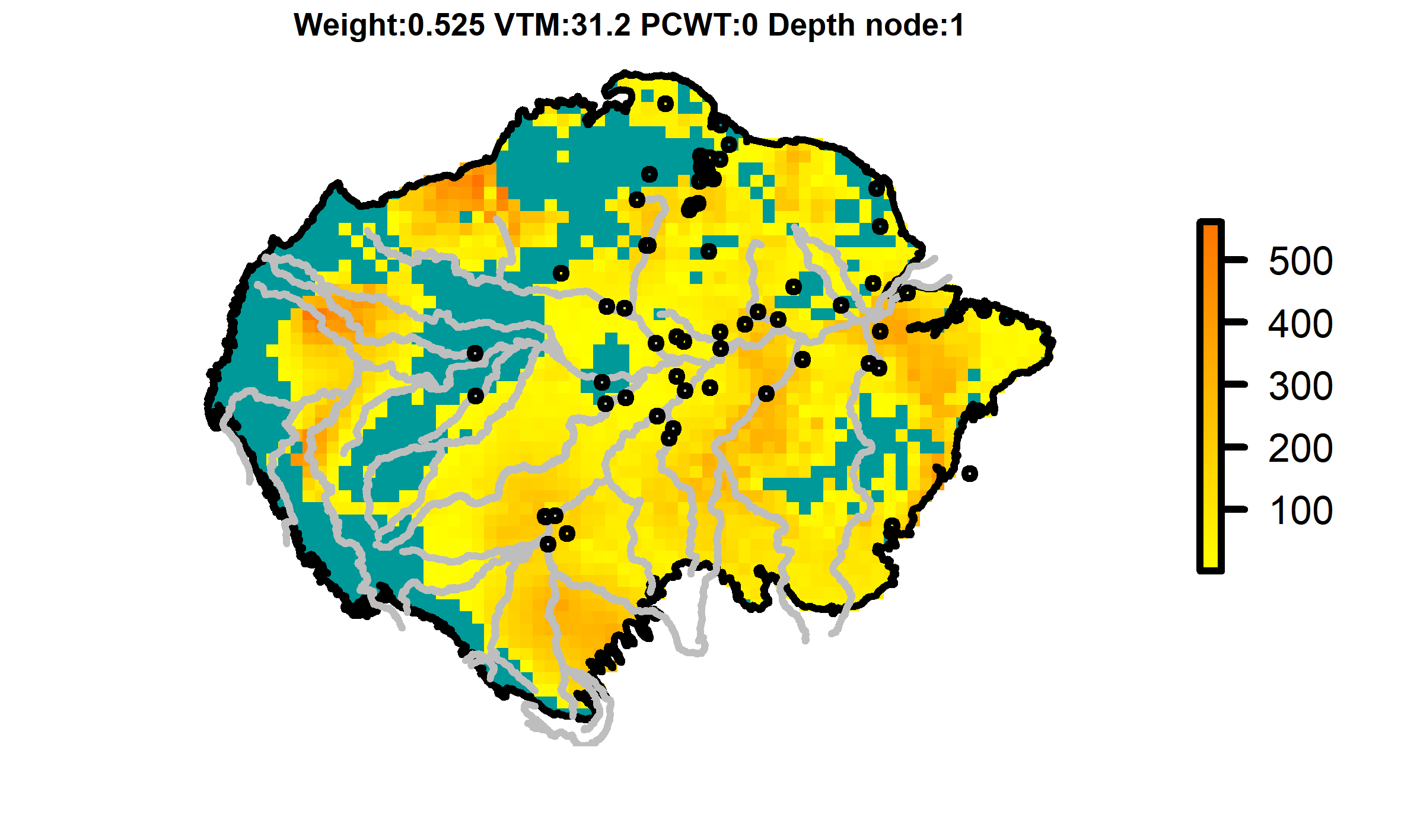

Supplement: S1 Data — (ZIP) [file pone.0286502.s002.zip › maps/map 3.png]

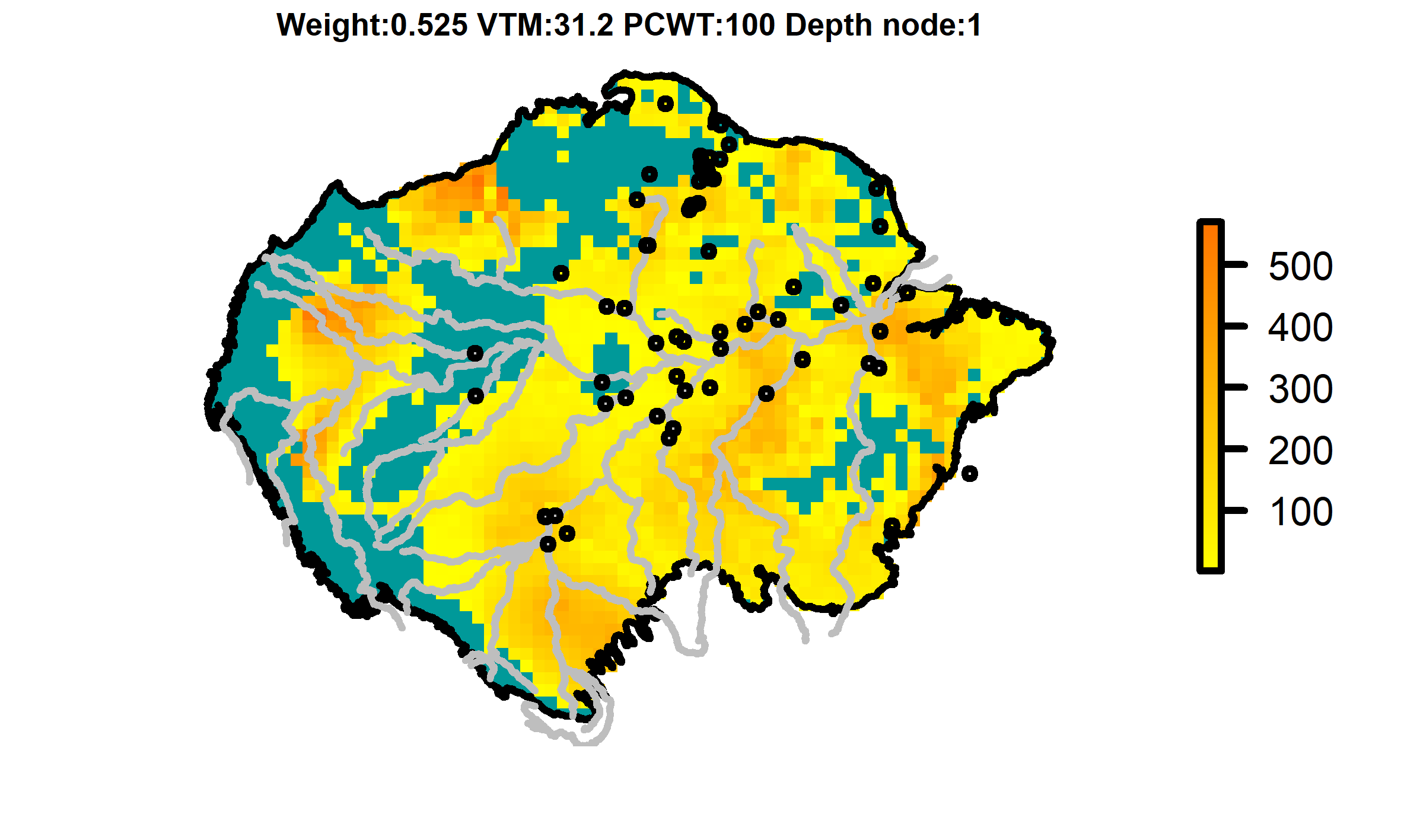

Supplement: S1 Data — (ZIP) [file pone.0286502.s002.zip › maps/map 7.png]

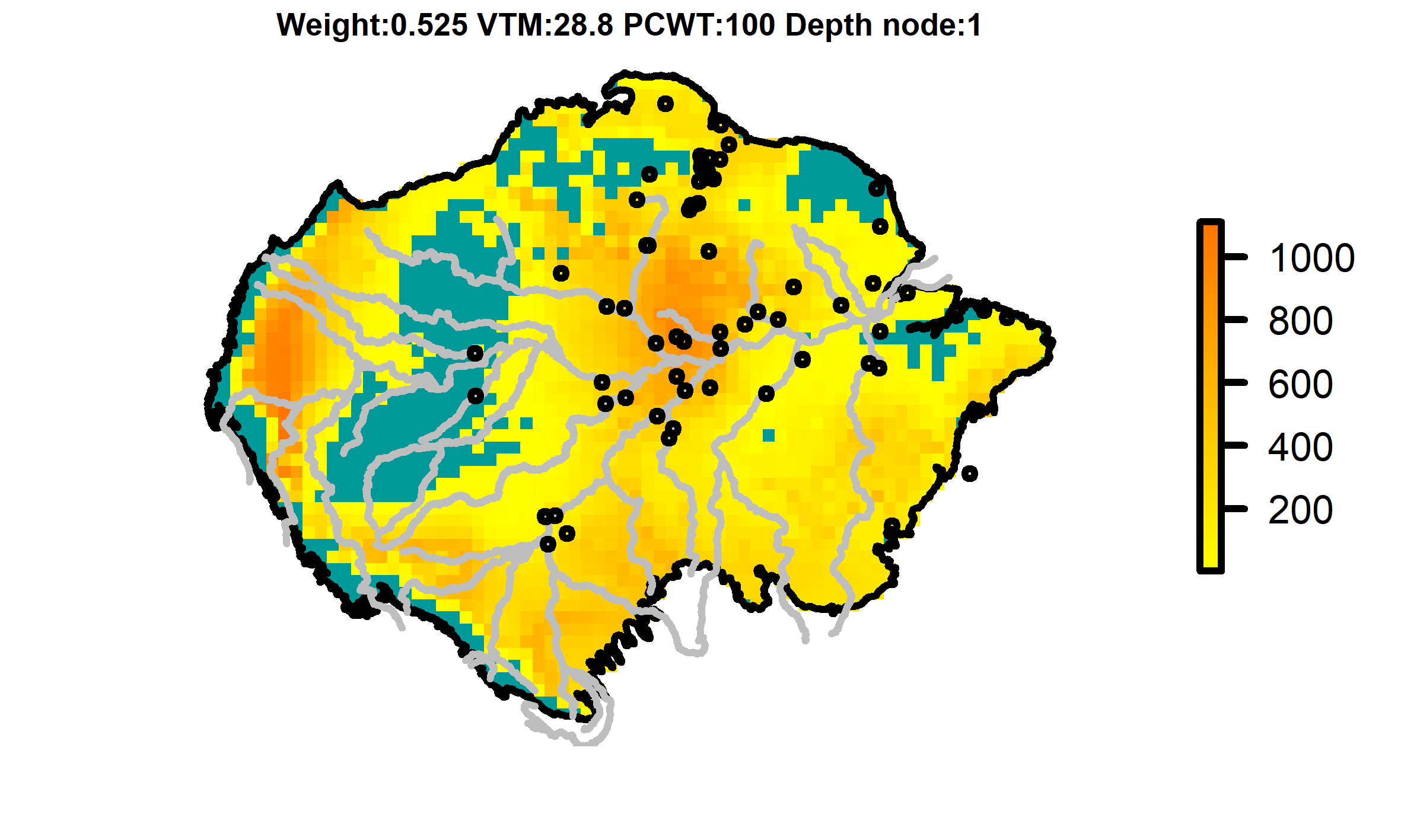

Supplement: S1 Data — (ZIP) [file pone.0286502.s002.zip › maps/map 37.png]

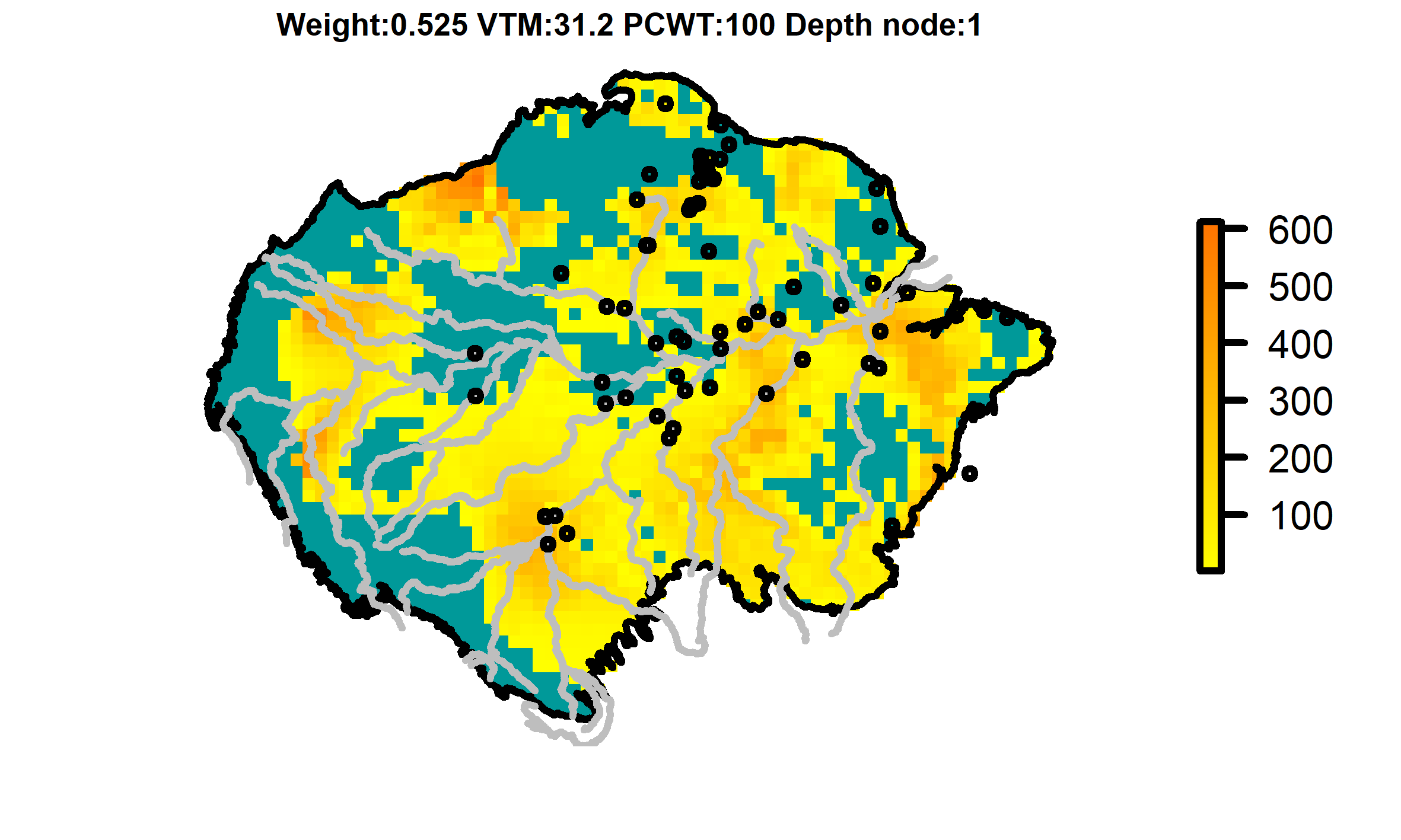

Supplement: S1 Data — (ZIP) [file pone.0286502.s002.zip › maps/map 23.png]

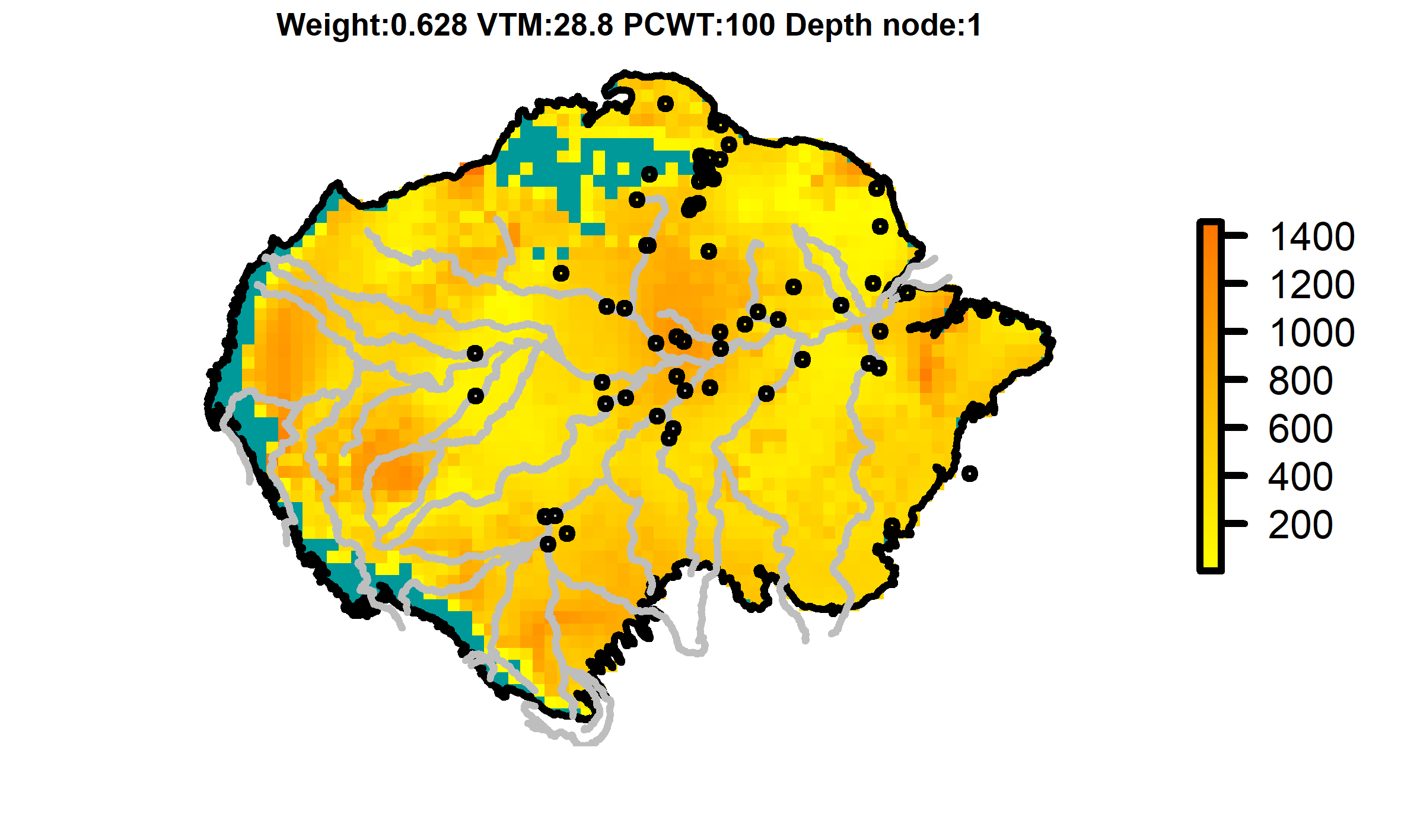

Supplement: S1 Data — (ZIP) [file pone.0286502.s002.zip › maps/map 22.png]

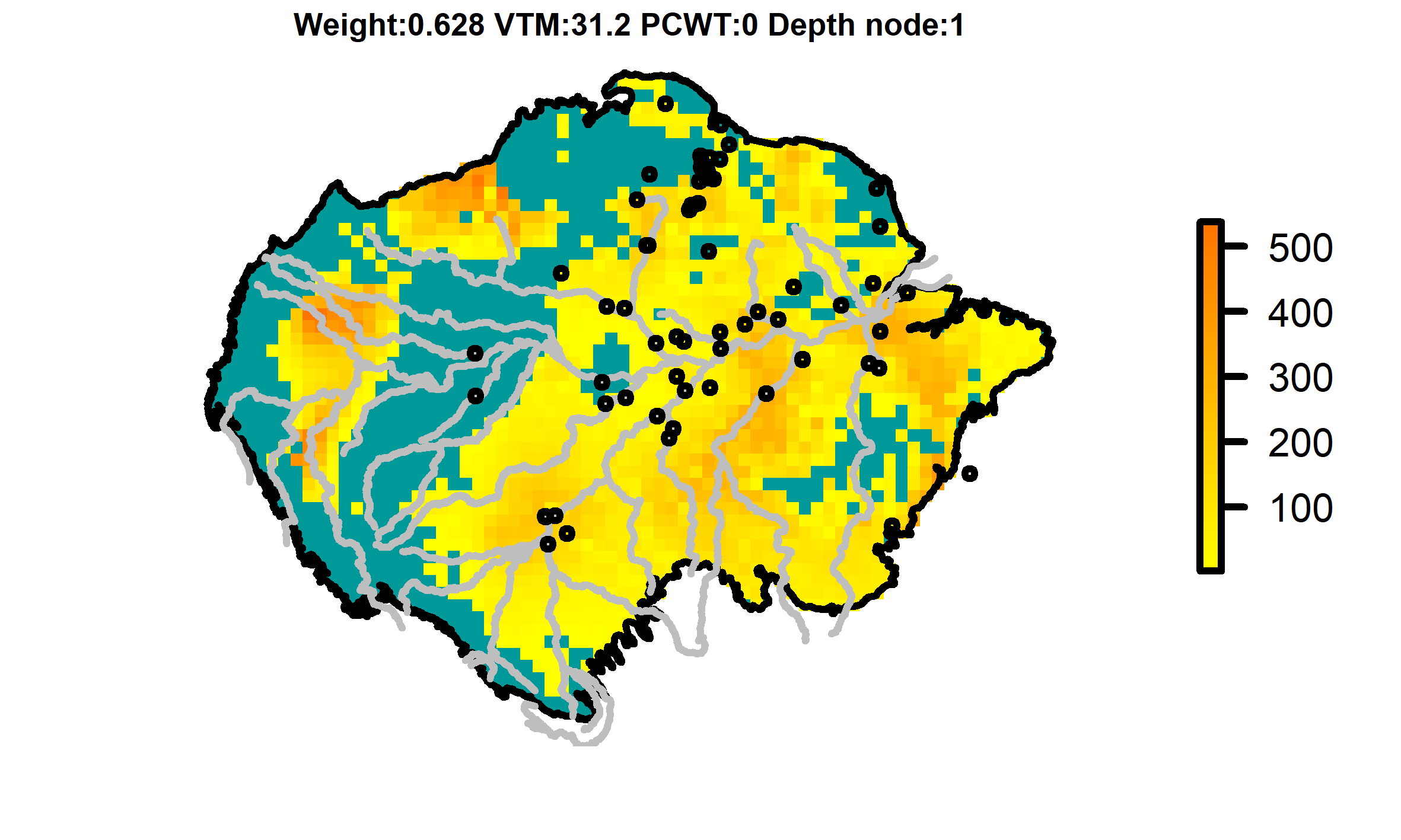

Supplement: S1 Data — (ZIP) [file pone.0286502.s002.zip › maps/map 36.png]

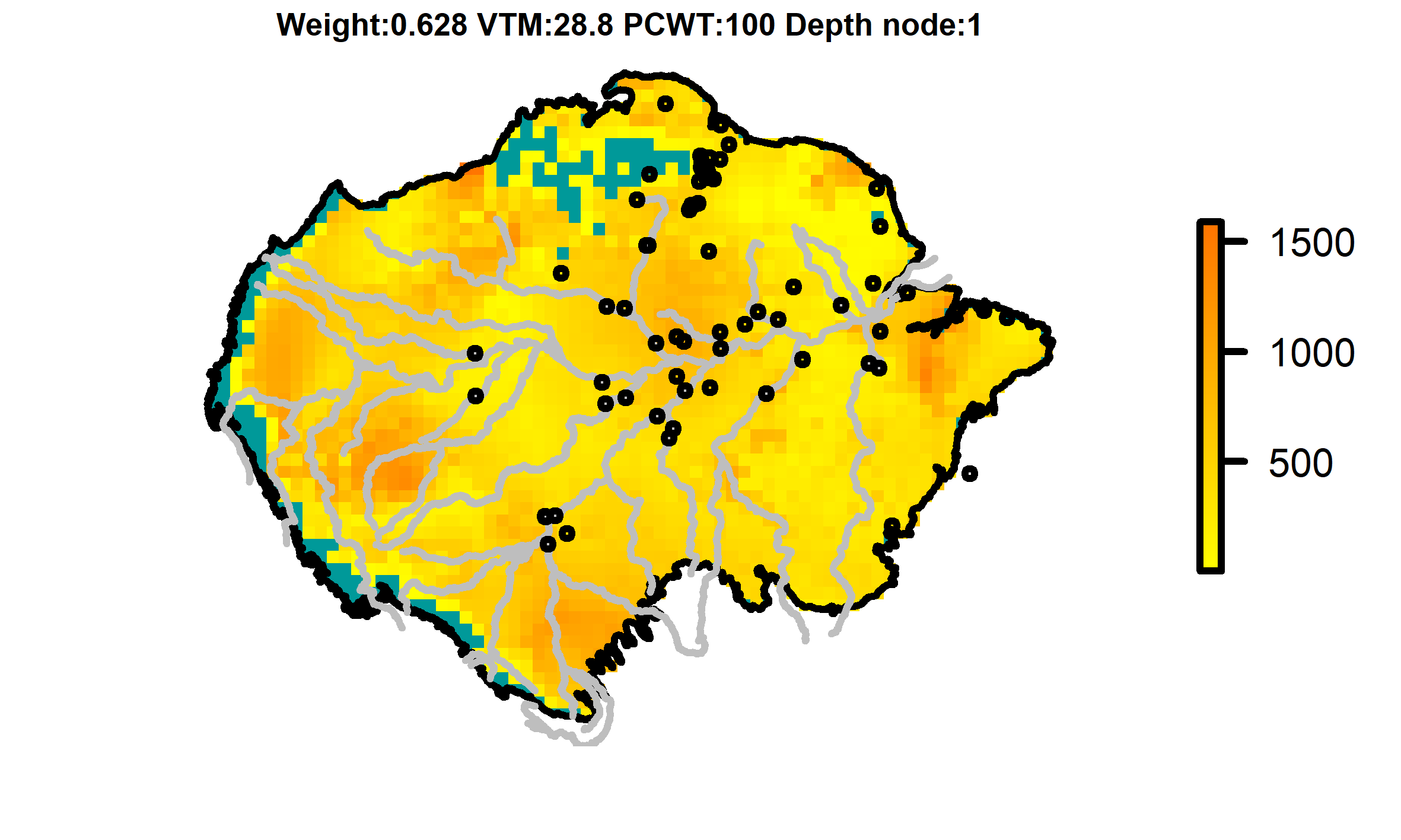

Supplement: S1 Data — (ZIP) [file pone.0286502.s002.zip › maps/map 6.png]

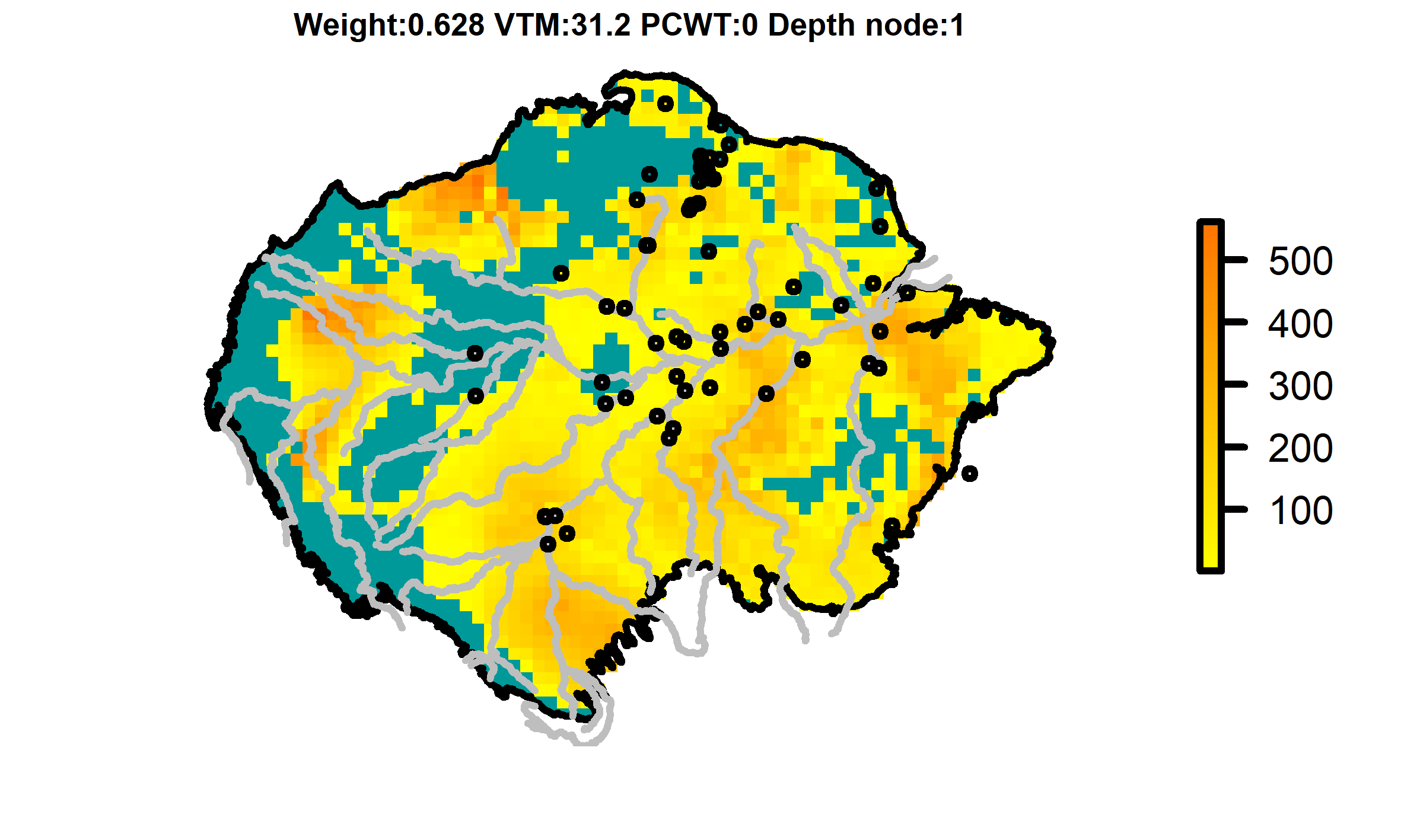

Supplement: S1 Data — (ZIP) [file pone.0286502.s002.zip › maps/map 4.png]

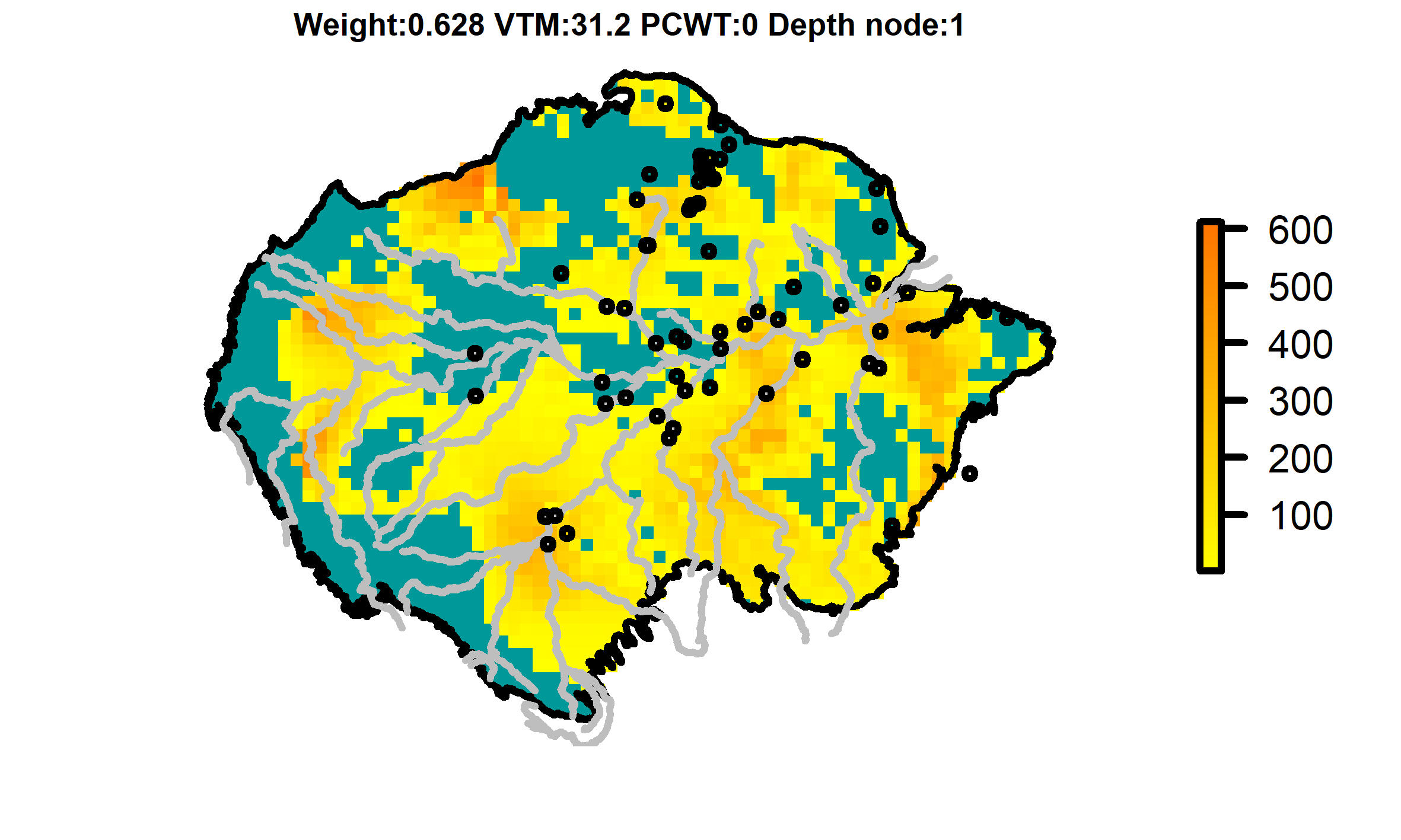

Supplement: S1 Data — (ZIP) [file pone.0286502.s002.zip › maps/map 20.png]

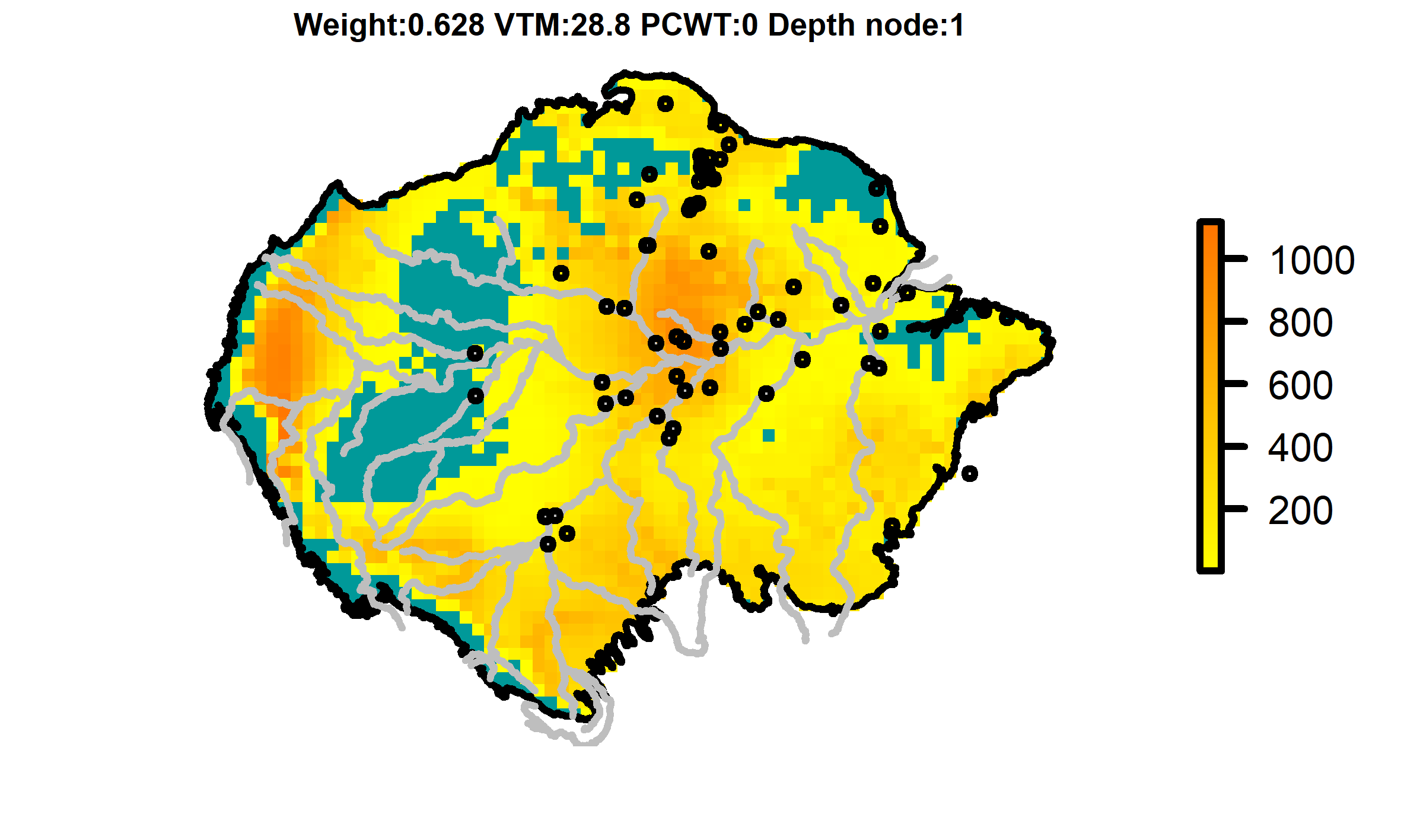

Supplement: S1 Data — (ZIP) [file pone.0286502.s002.zip › maps/map 34.png]

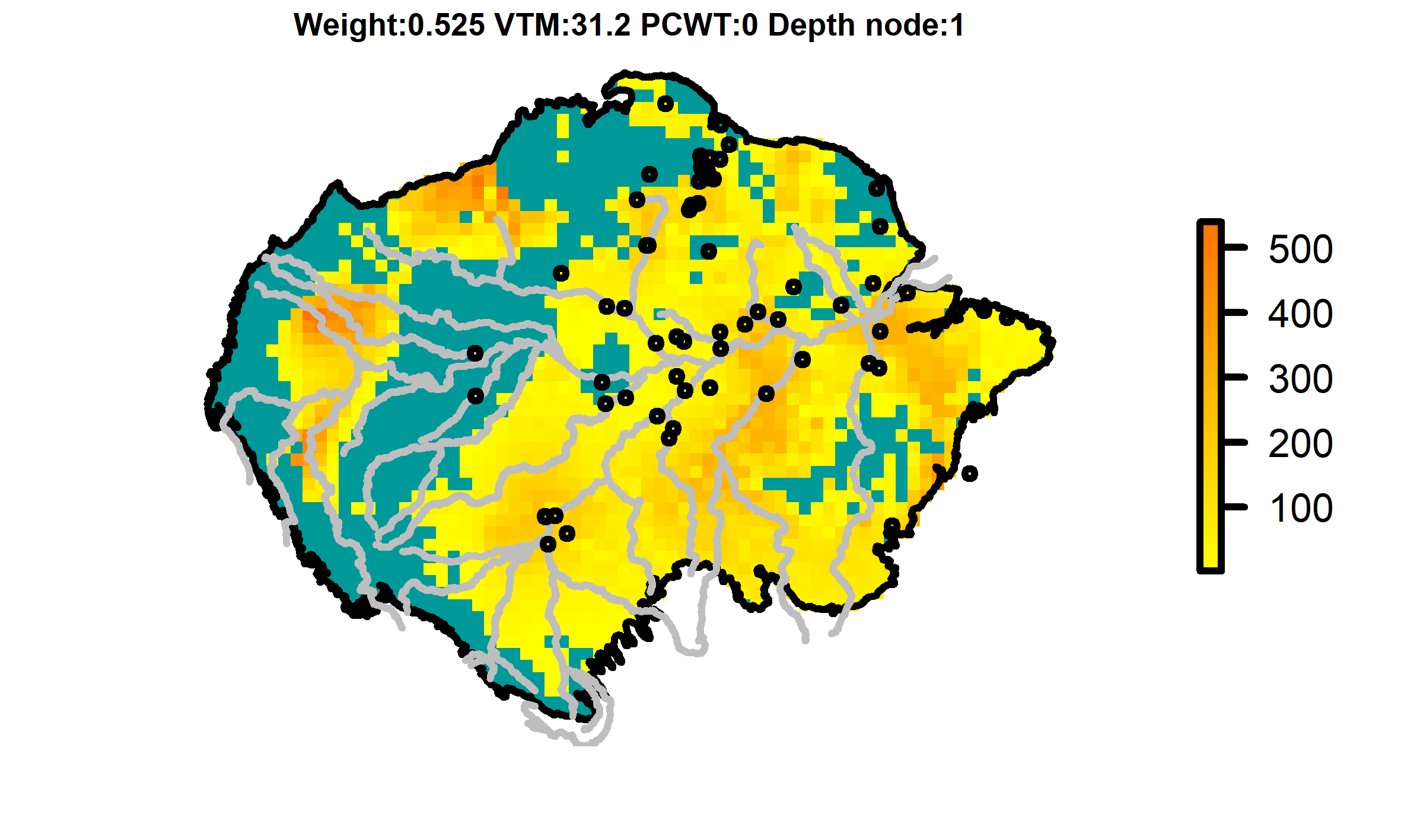

Supplement: S1 Data — (ZIP) [file pone.0286502.s002.zip › maps/map 35.png]

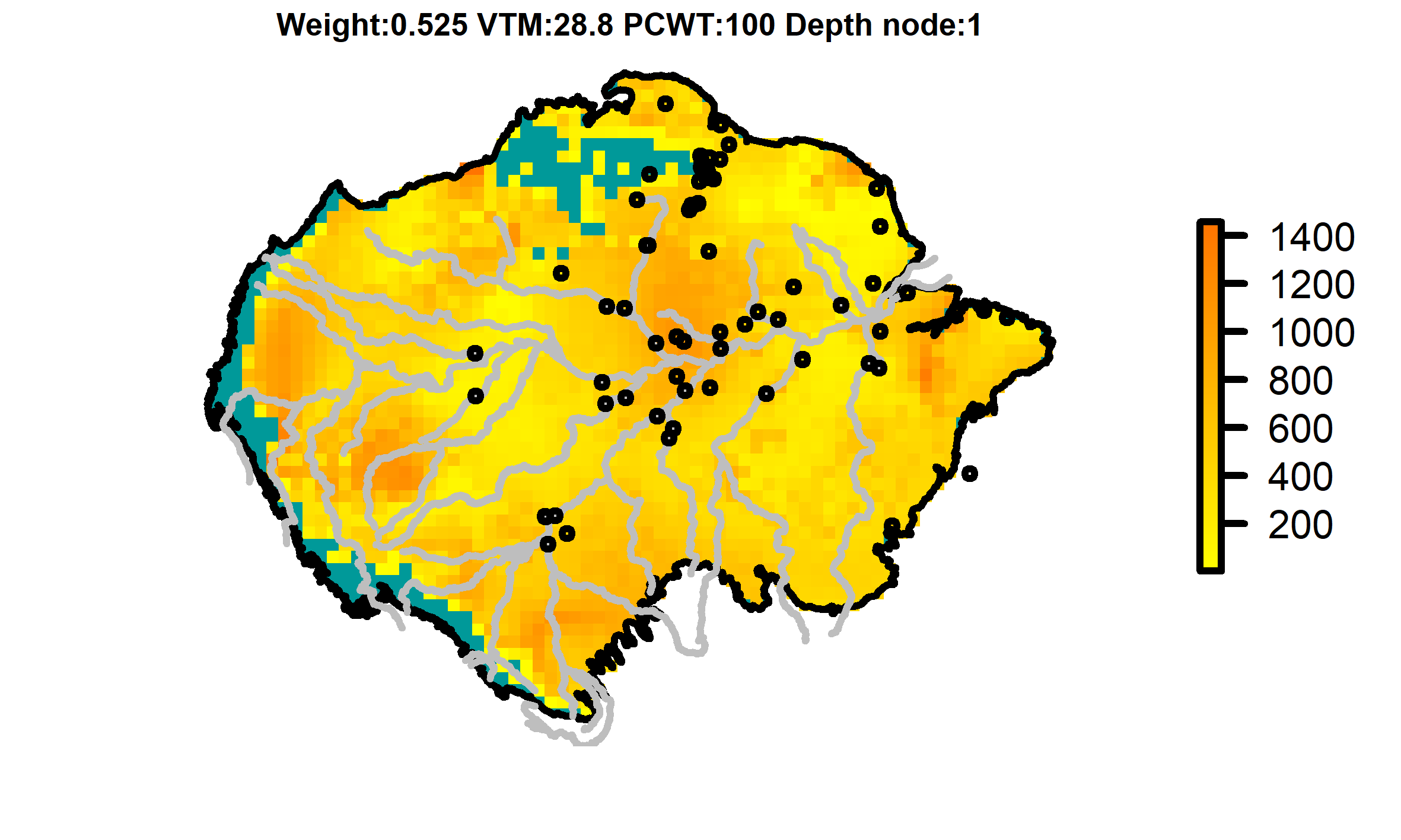

Supplement: S1 Data — (ZIP) [file pone.0286502.s002.zip › maps/map 21.png]

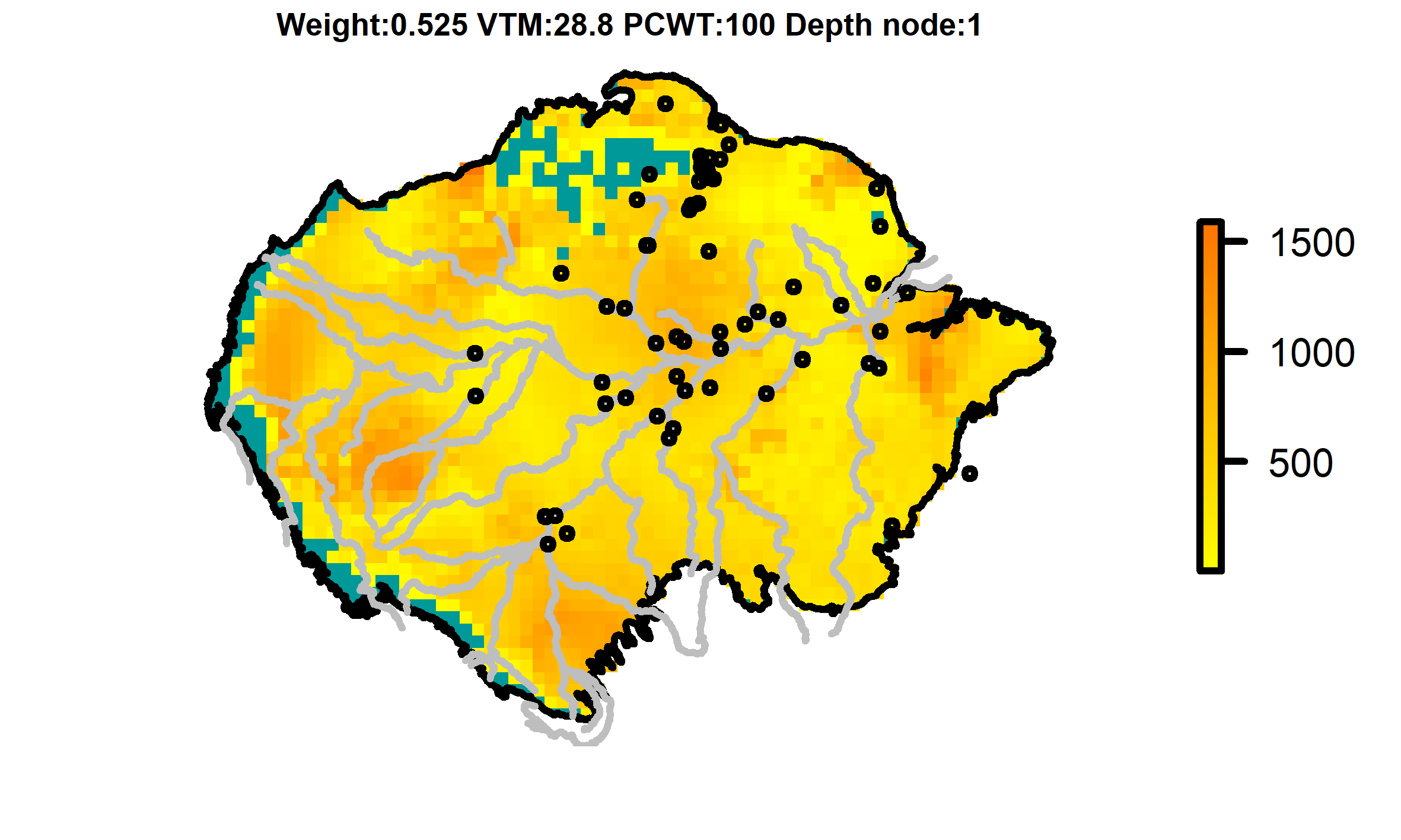

Supplement: S1 Data — (ZIP) [file pone.0286502.s002.zip › maps/map 5.png]

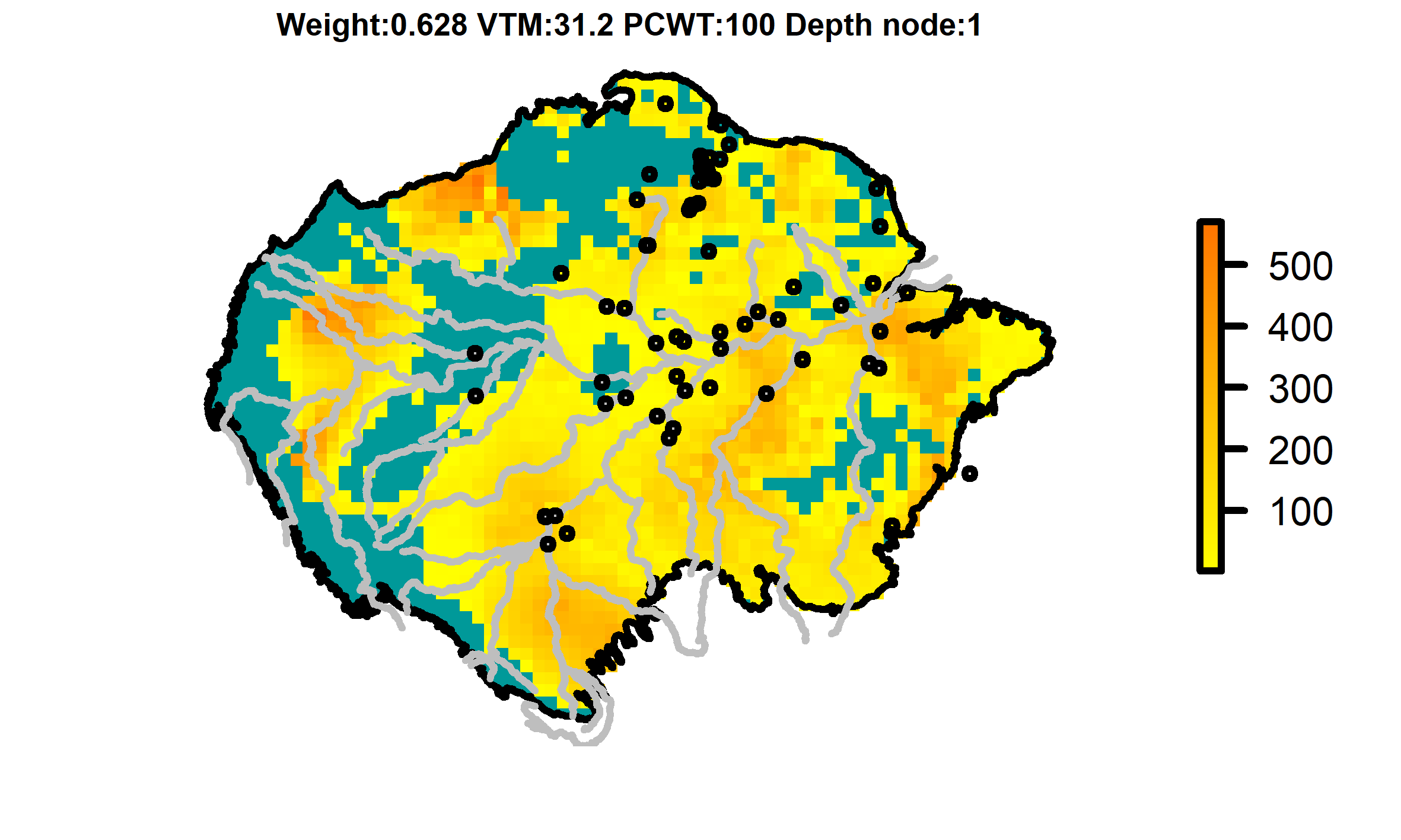

Supplement: S1 Data — (ZIP) [file pone.0286502.s002.zip › maps/map 8.png]

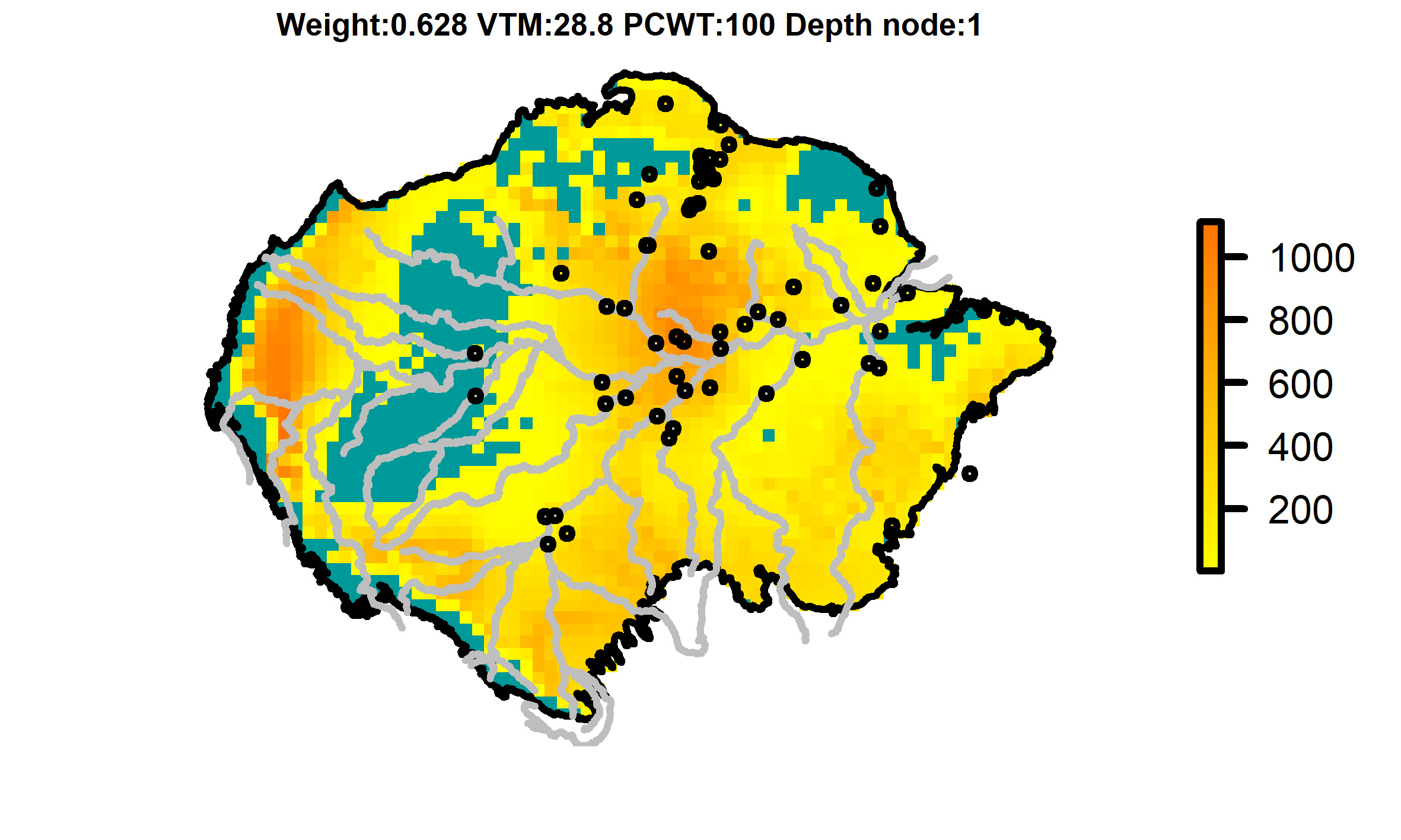

Supplement: S1 Data — (ZIP) [file pone.0286502.s002.zip › maps/map 38.png]

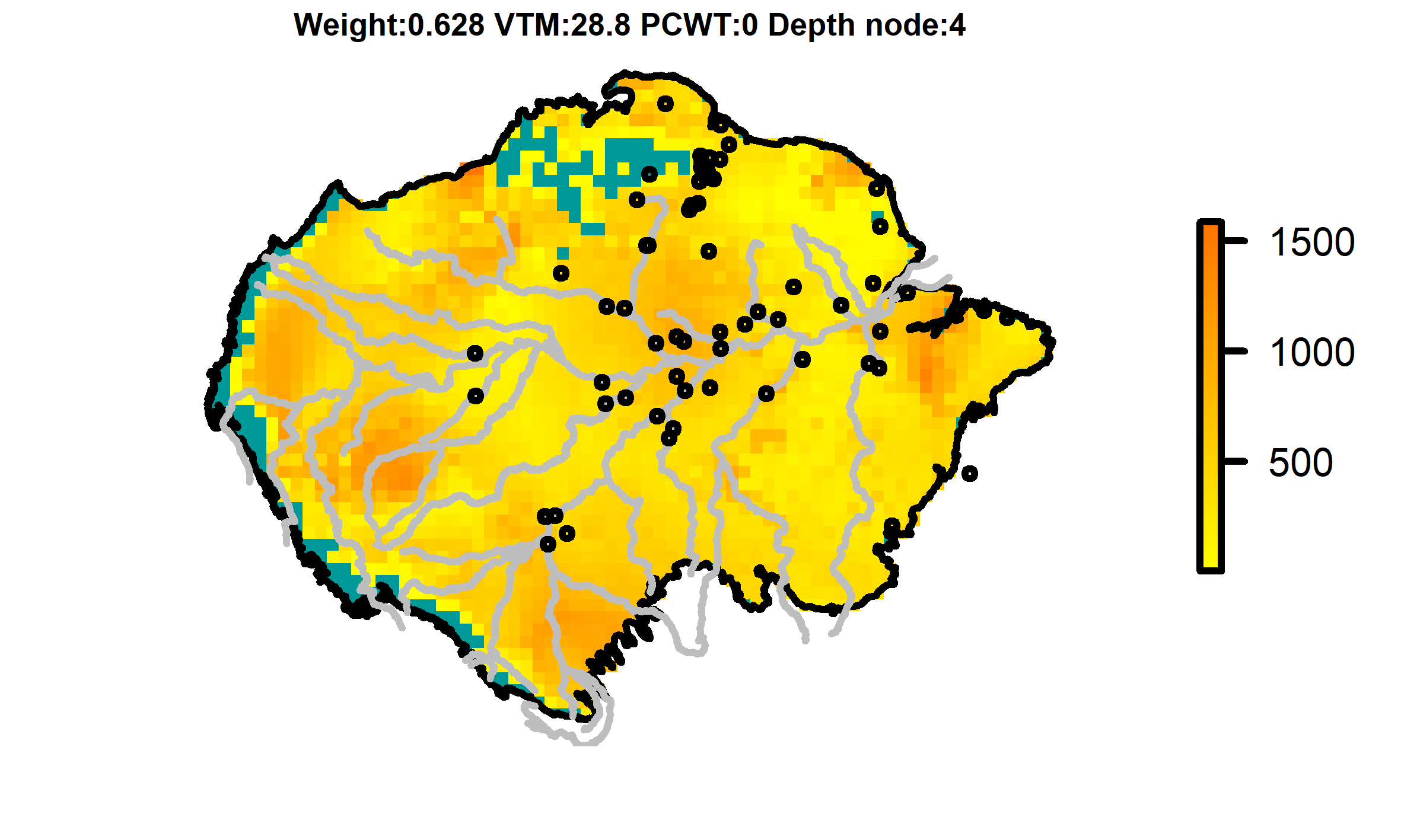

Supplement: S1 Data — (ZIP) [file pone.0286502.s002.zip › maps/map 10.png]

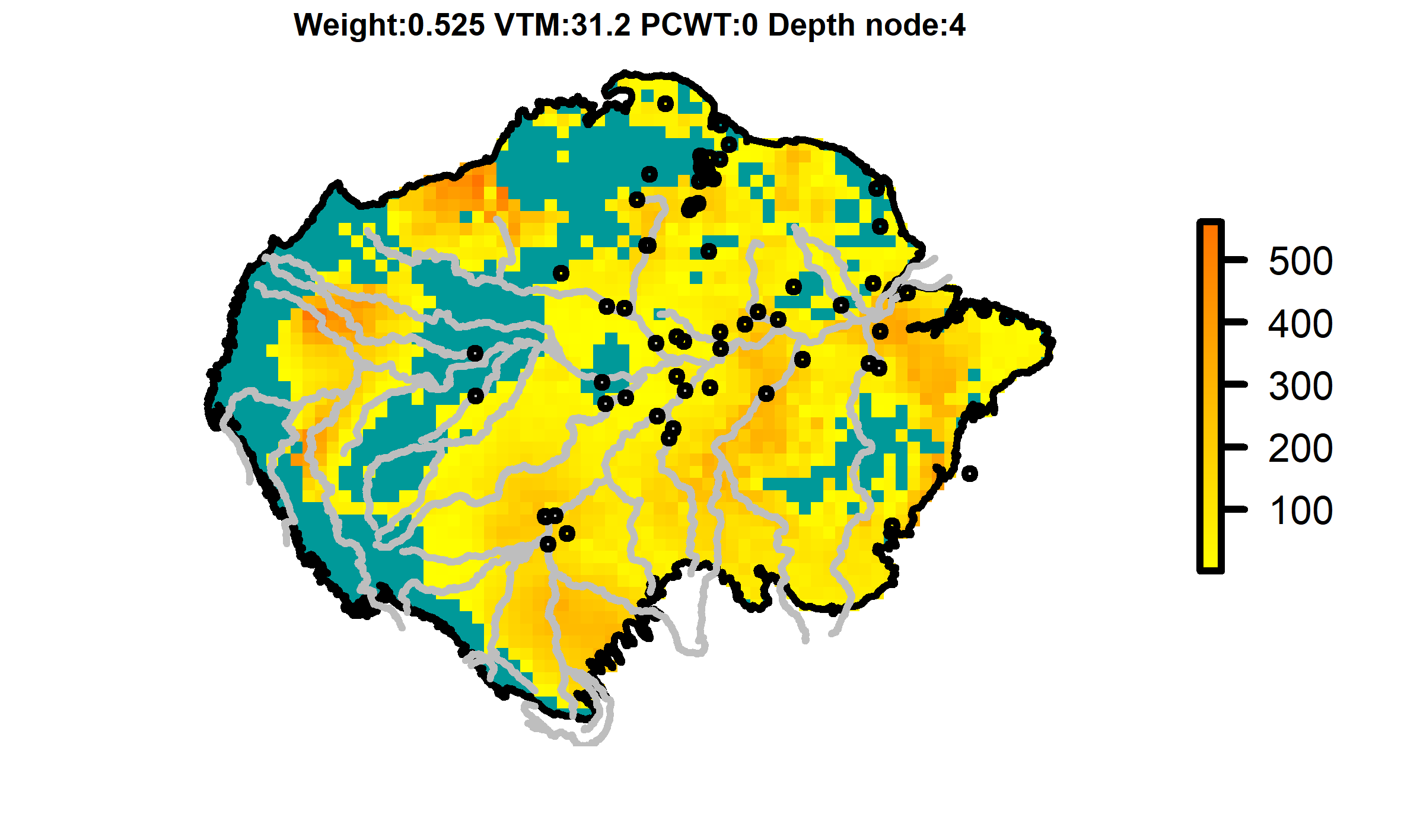

Supplement: S1 Data — (ZIP) [file pone.0286502.s002.zip › maps/map 11.png]

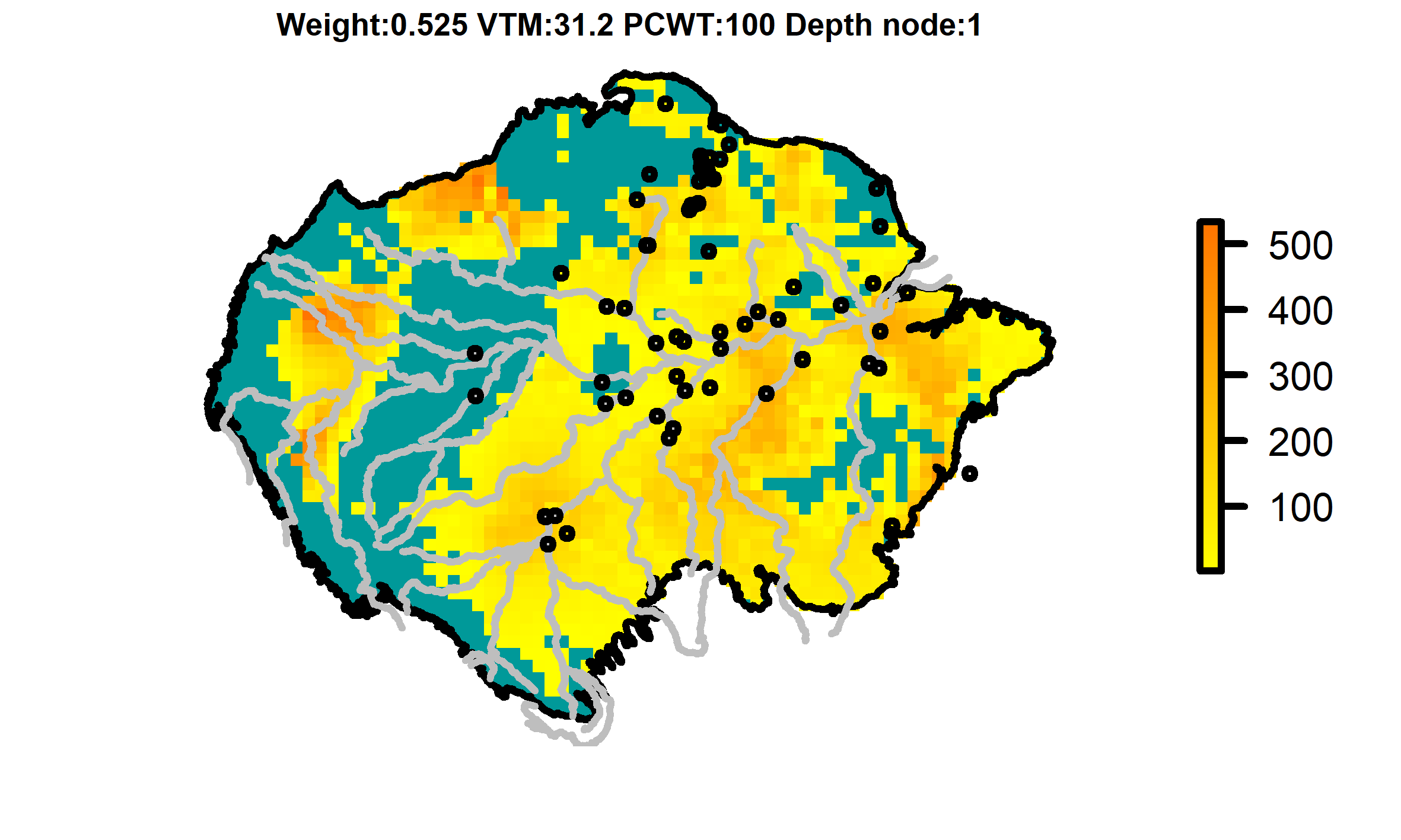

Supplement: S1 Data — (ZIP) [file pone.0286502.s002.zip › maps/map 39.png]

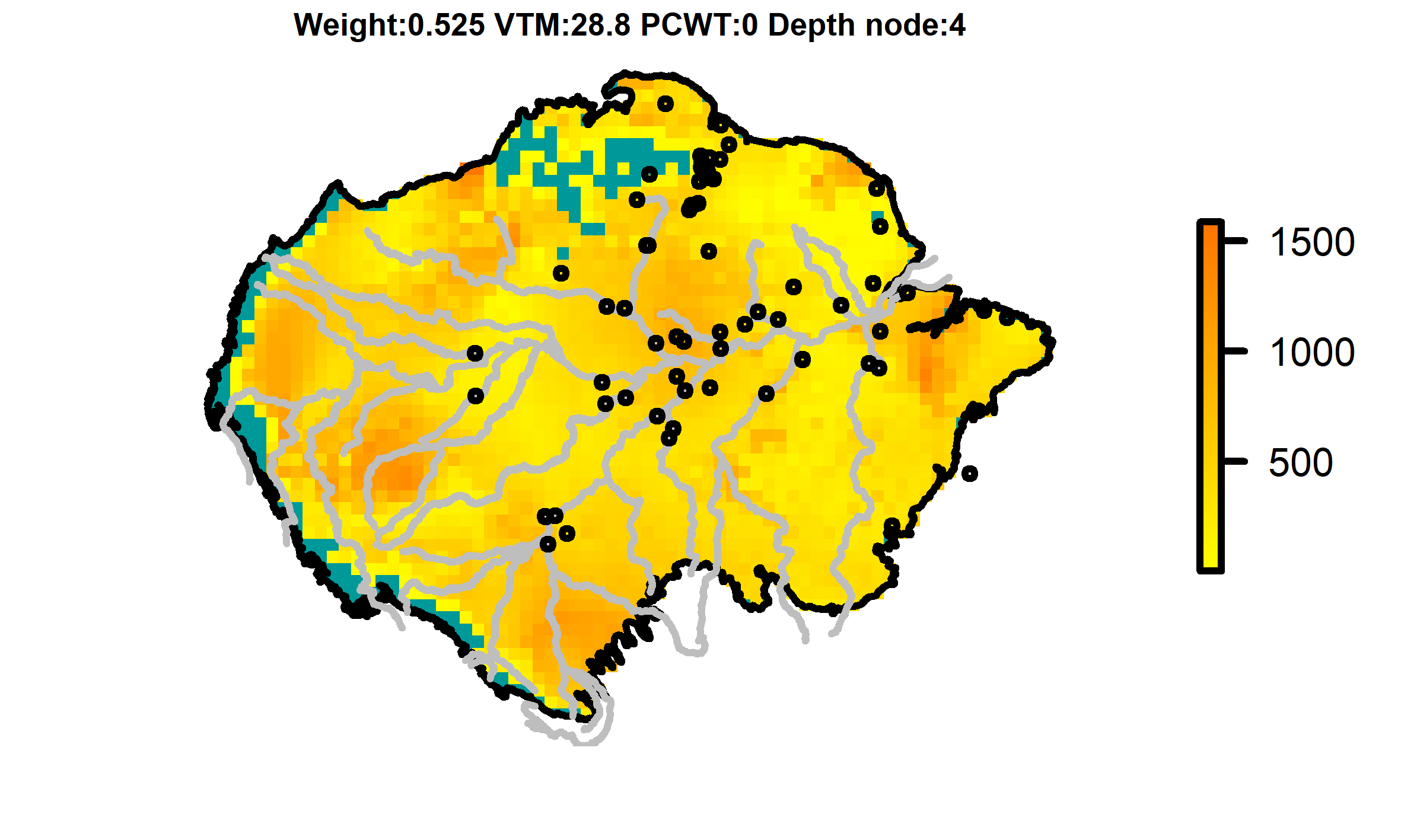

Supplement: S1 Data — (ZIP) [file pone.0286502.s002.zip › maps/map 9.png]

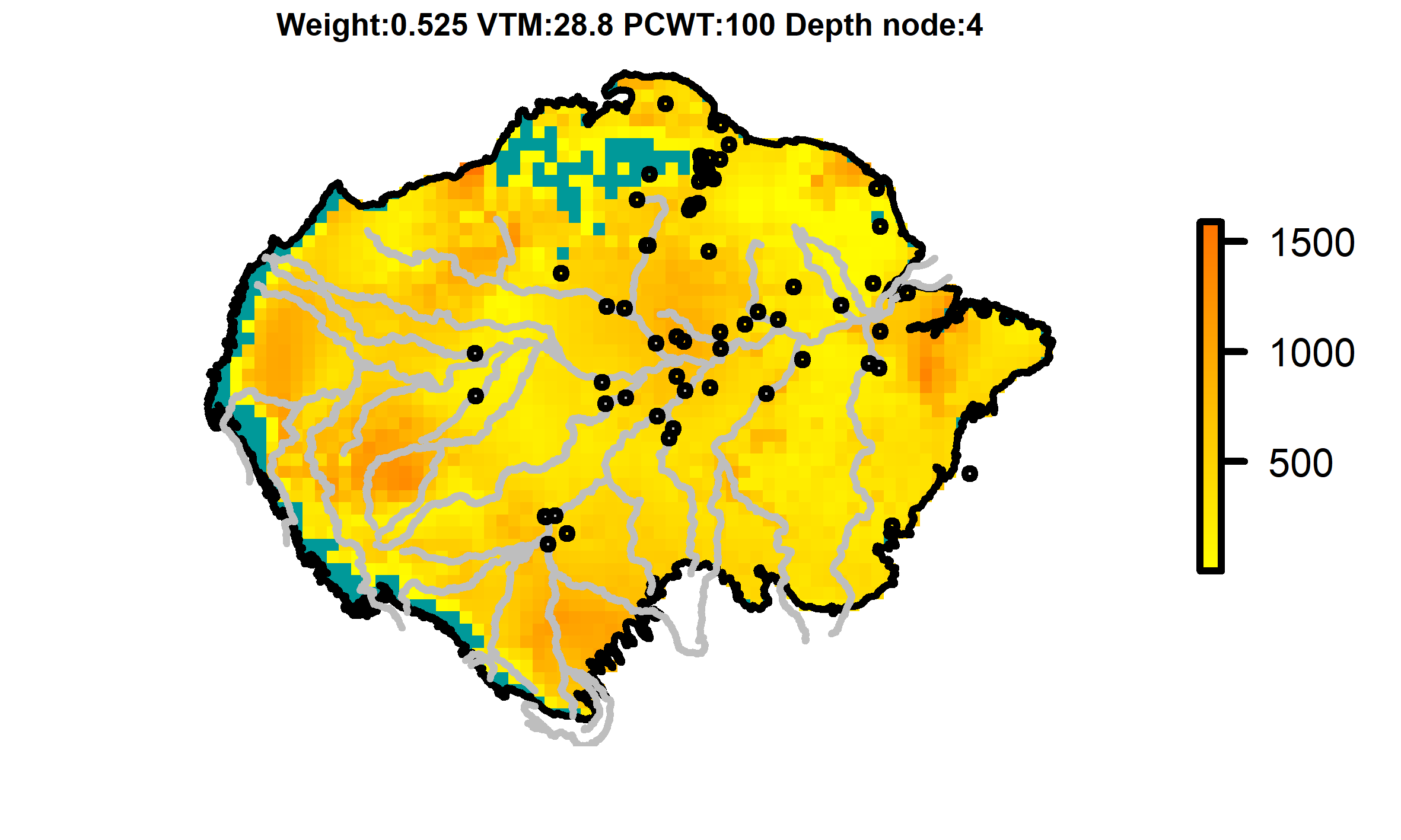

Supplement: S1 Data — (ZIP) [file pone.0286502.s002.zip › maps/map 13.png]

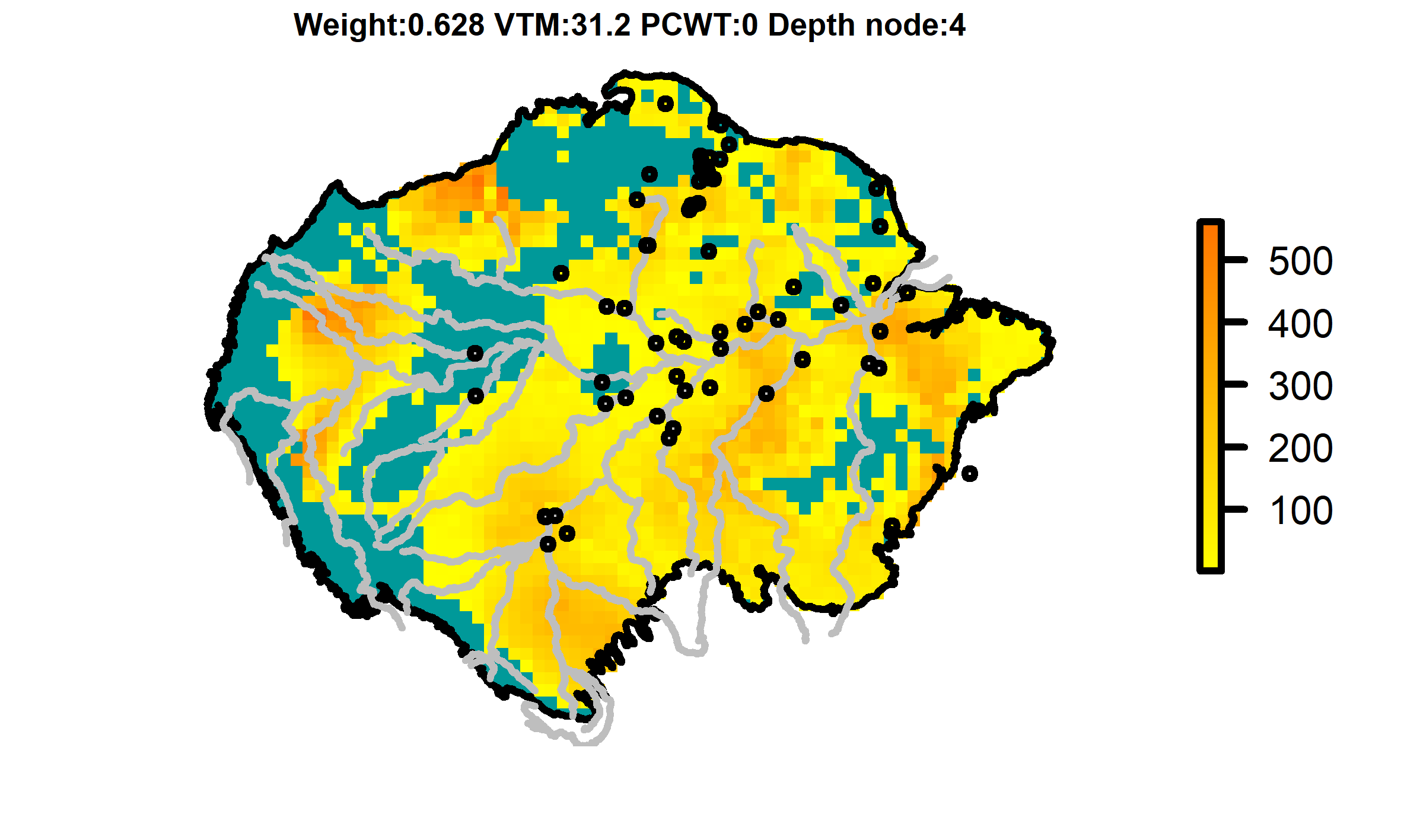

Supplement: S1 Data — (ZIP) [file pone.0286502.s002.zip › maps/map 12.png]

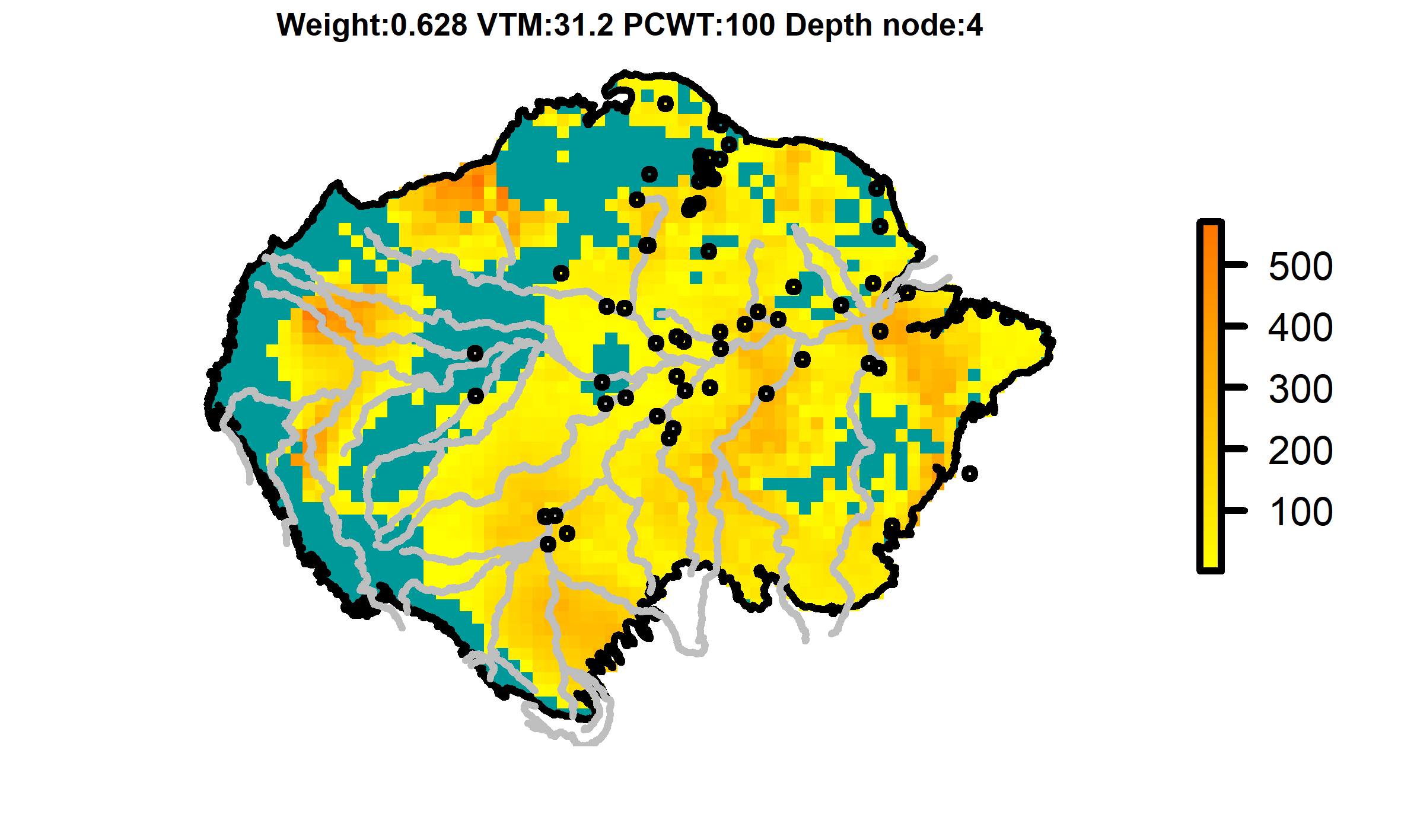

Supplement: S1 Data — (ZIP) [file pone.0286502.s002.zip › maps/map 16.png]

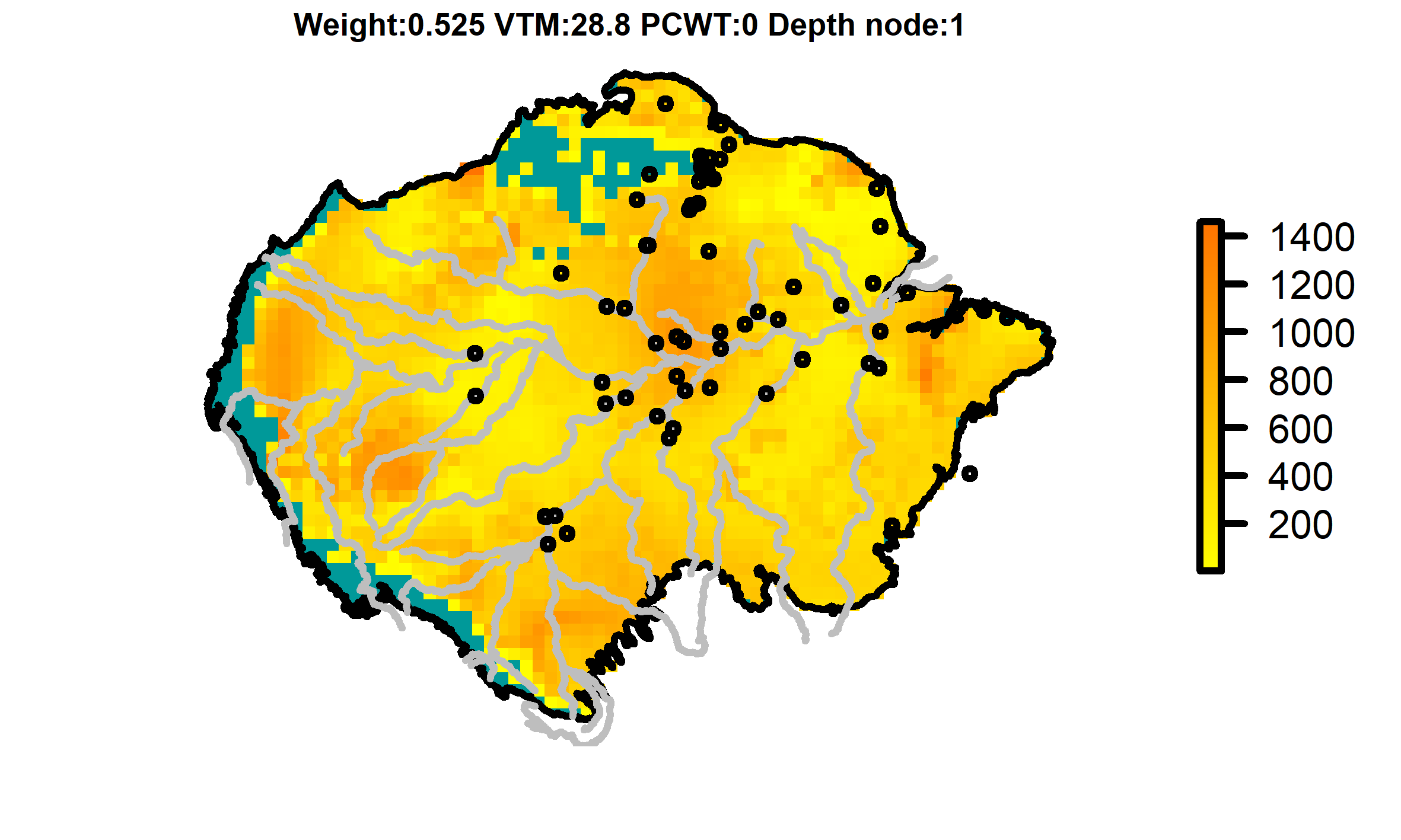

Supplement: S1 Data — (ZIP) [file pone.0286502.s002.zip › maps/map 17.png]

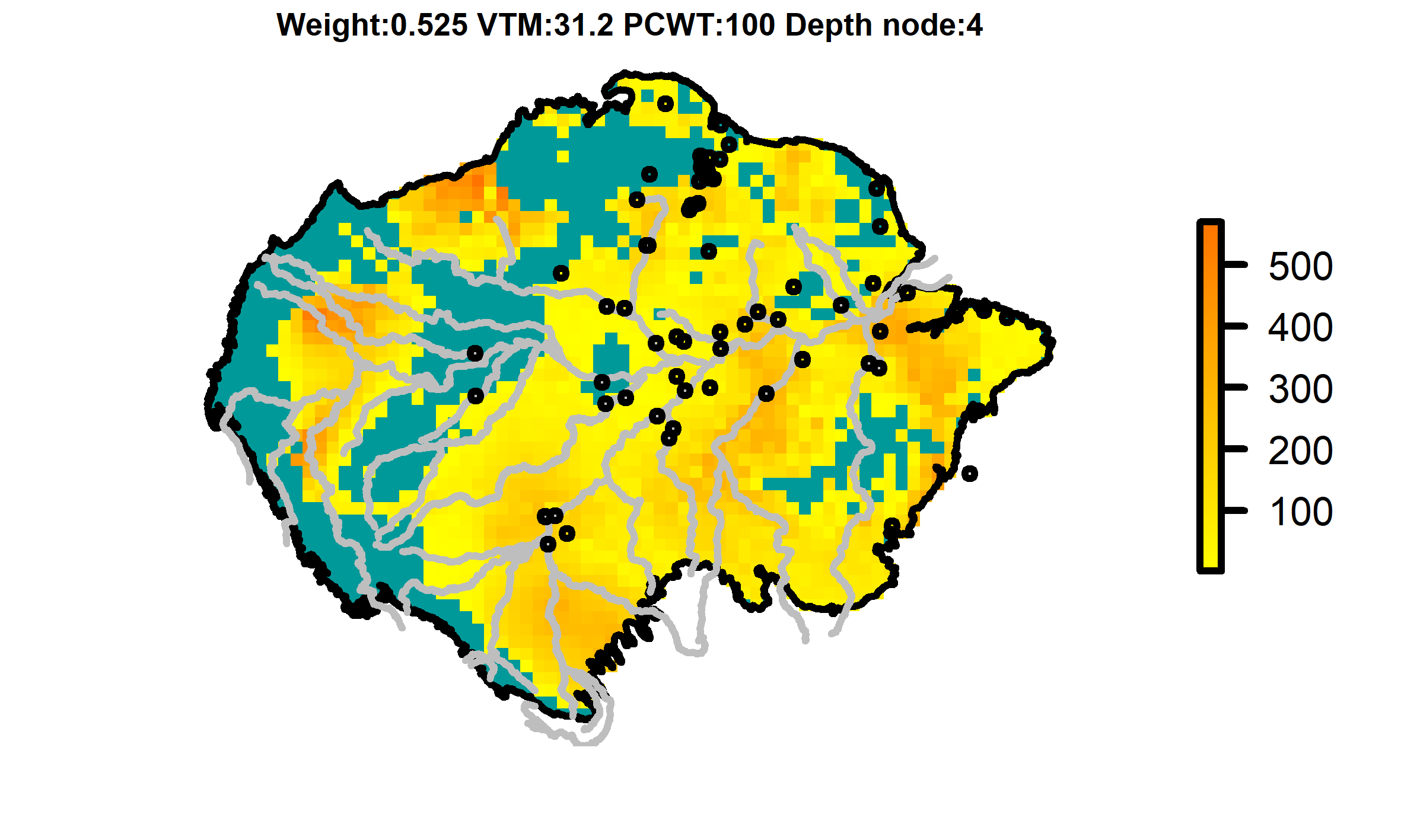

Supplement: S1 Data — (ZIP) [file pone.0286502.s002.zip › maps/map 15.png]

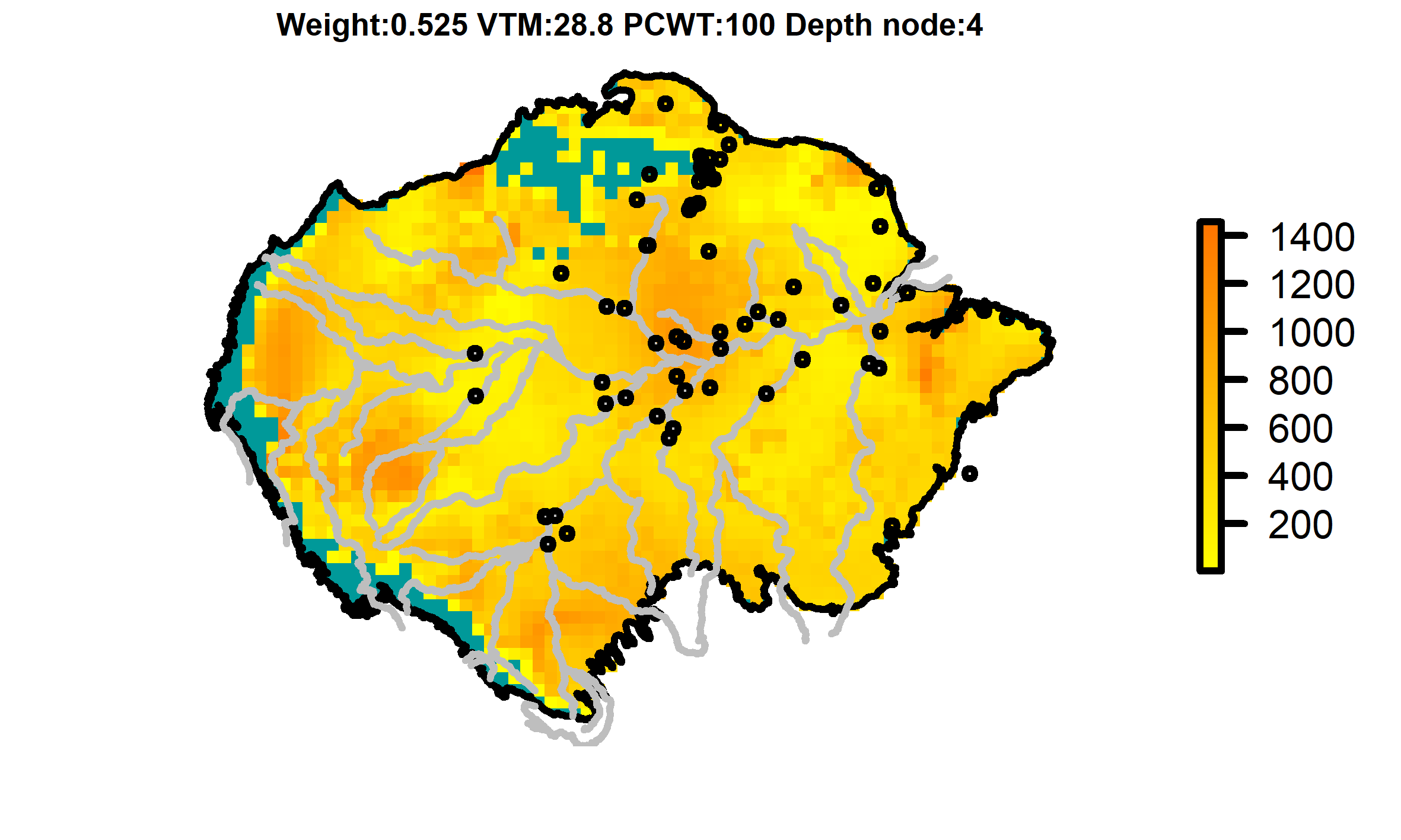

Supplement: S1 Data — (ZIP) [file pone.0286502.s002.zip › maps/map 29.png]

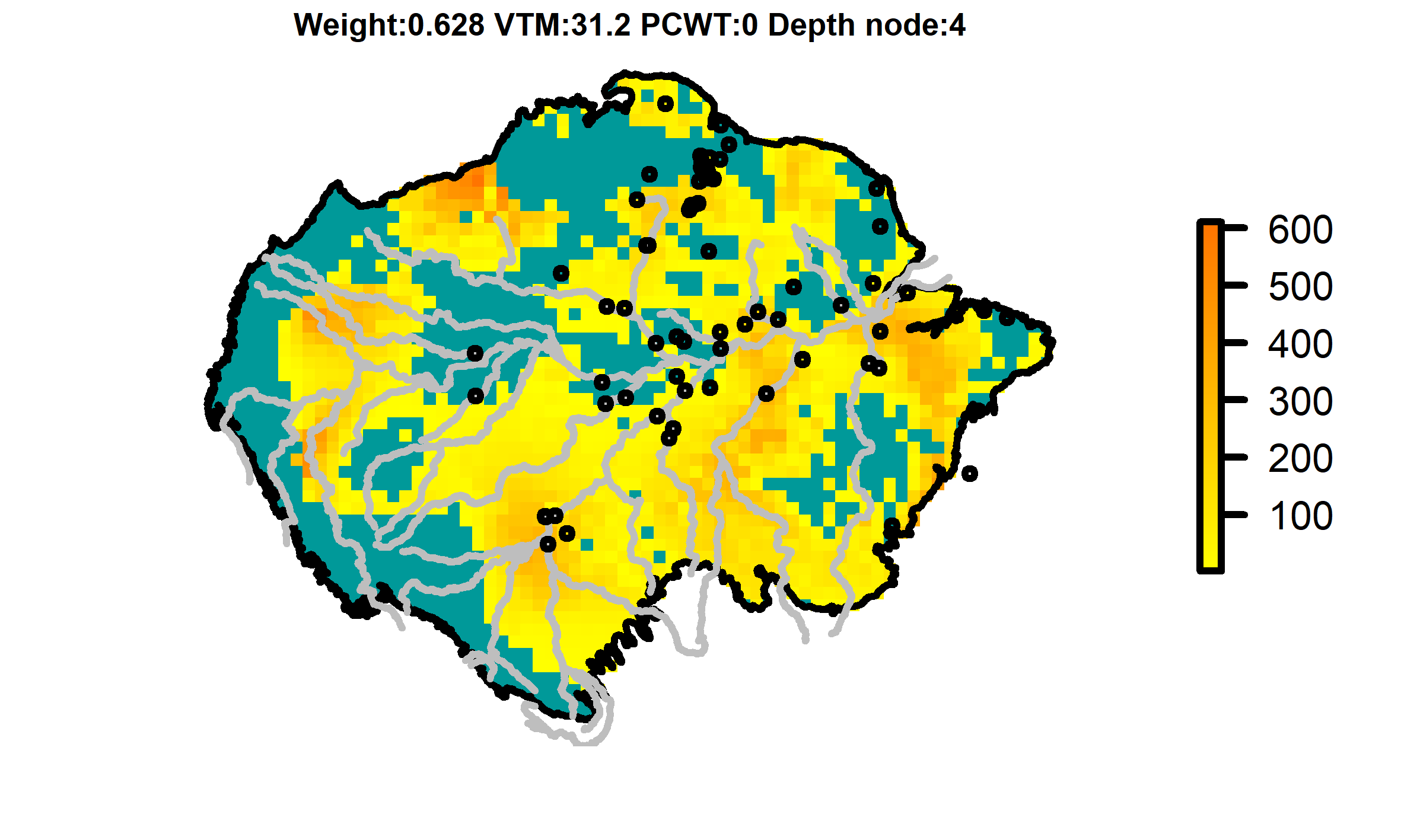

Supplement: S1 Data — (ZIP) [file pone.0286502.s002.zip › maps/map 28.png]

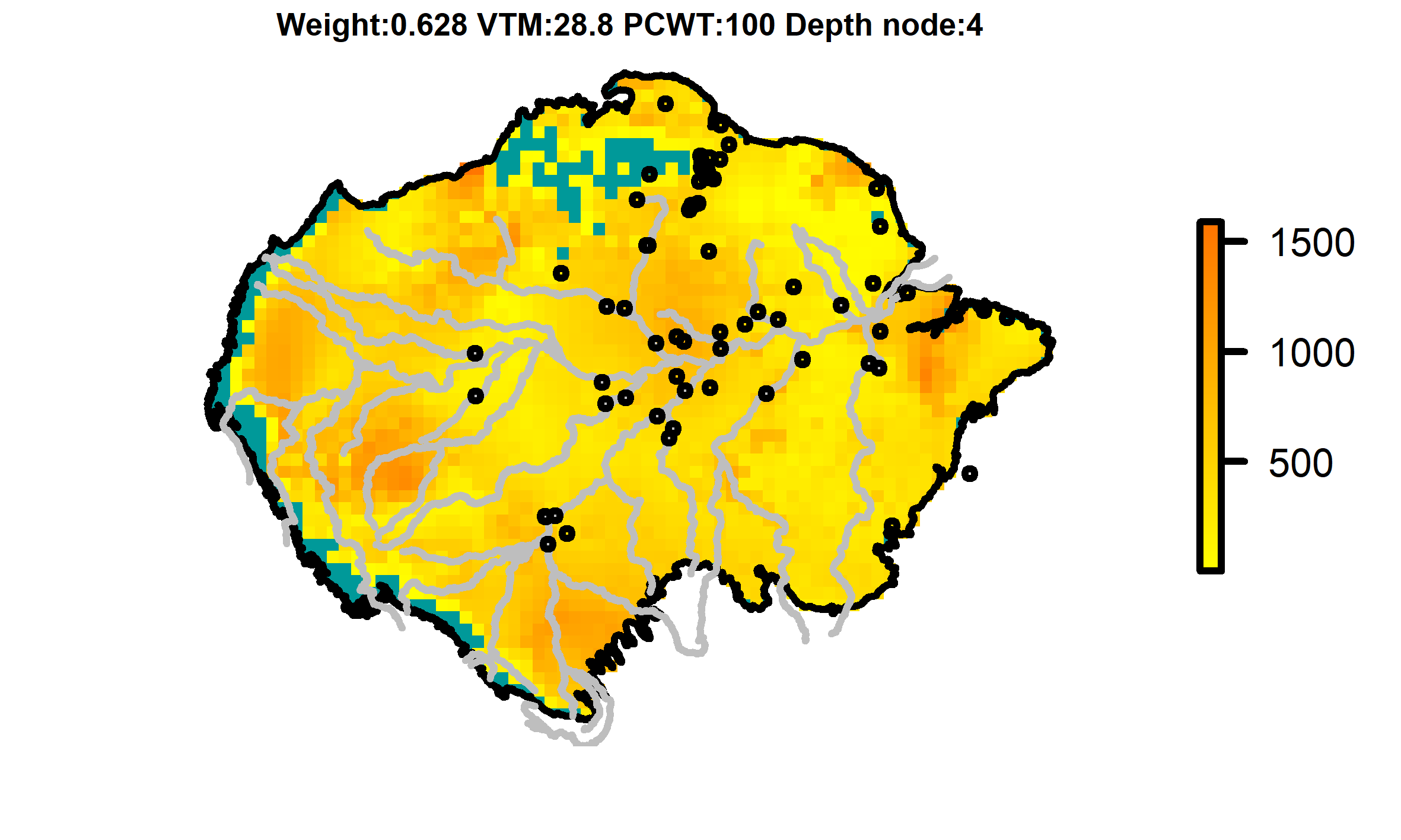

Supplement: S1 Data — (ZIP) [file pone.0286502.s002.zip › maps/map 14.png]

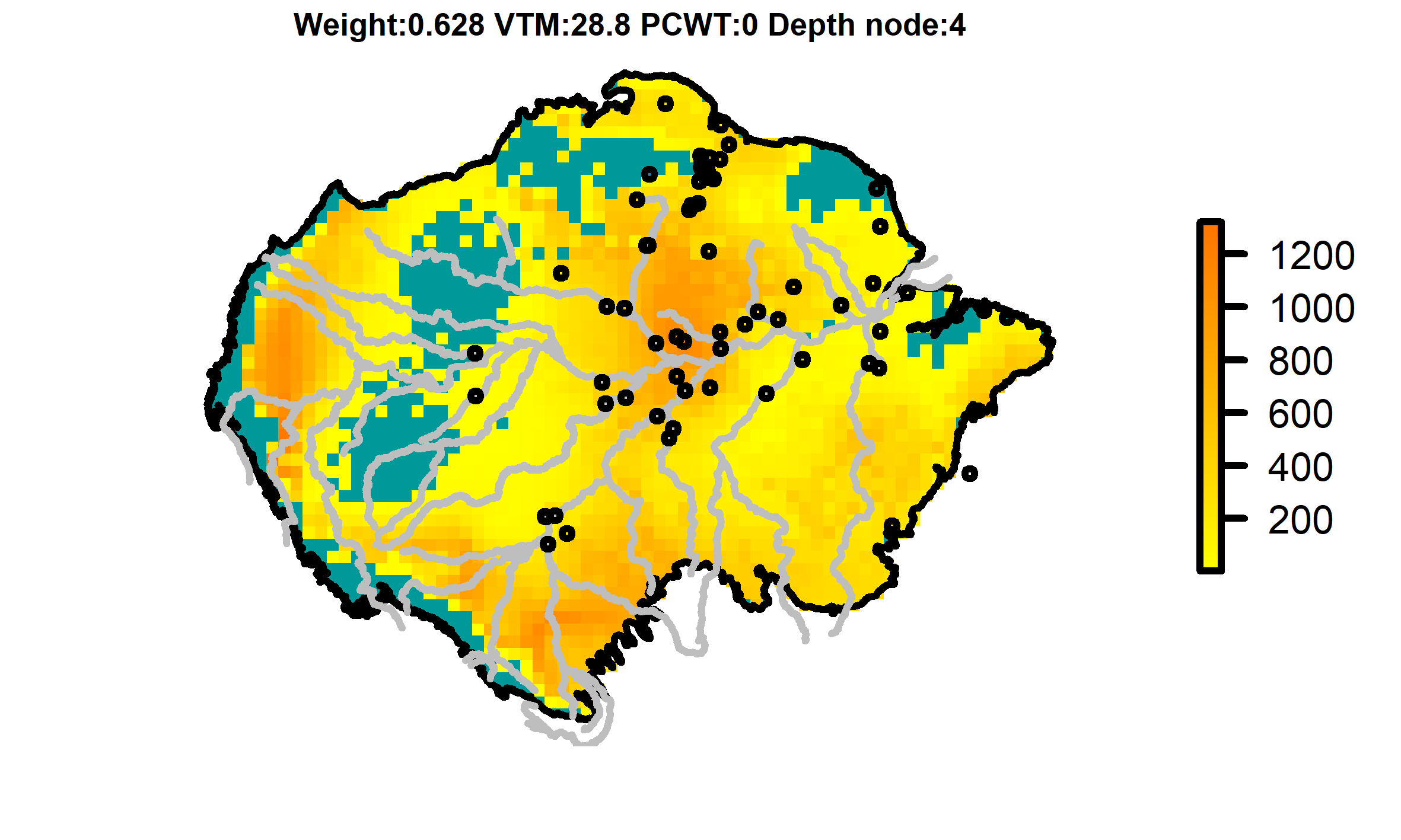

Supplement: S1 Data — (ZIP) [file pone.0286502.s002.zip › maps/map 58.png]

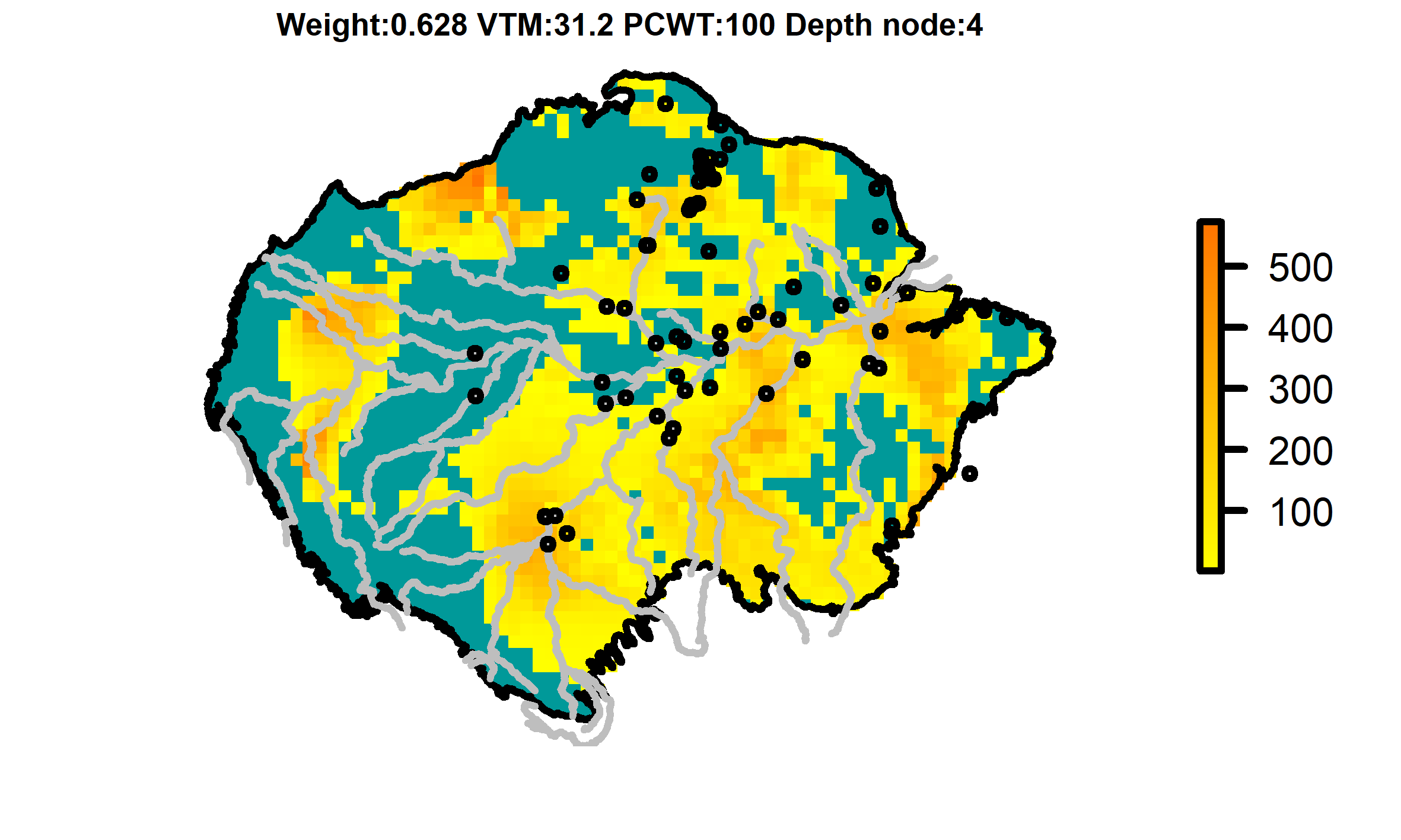

Supplement: S1 Data — (ZIP) [file pone.0286502.s002.zip › maps/map 64.png]

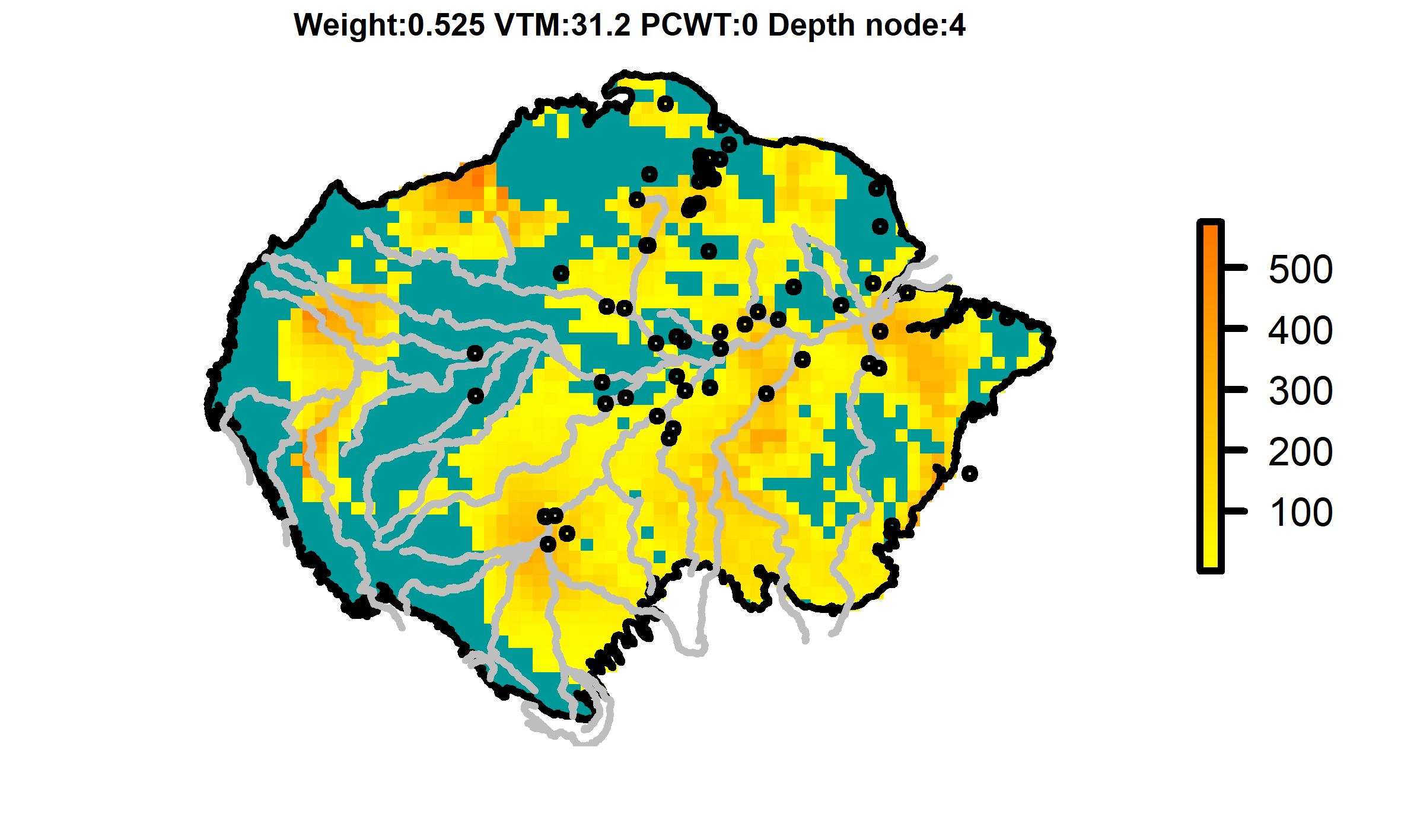

Supplement: S1 Data — (ZIP) [file pone.0286502.s002.zip › maps/map 59.png]

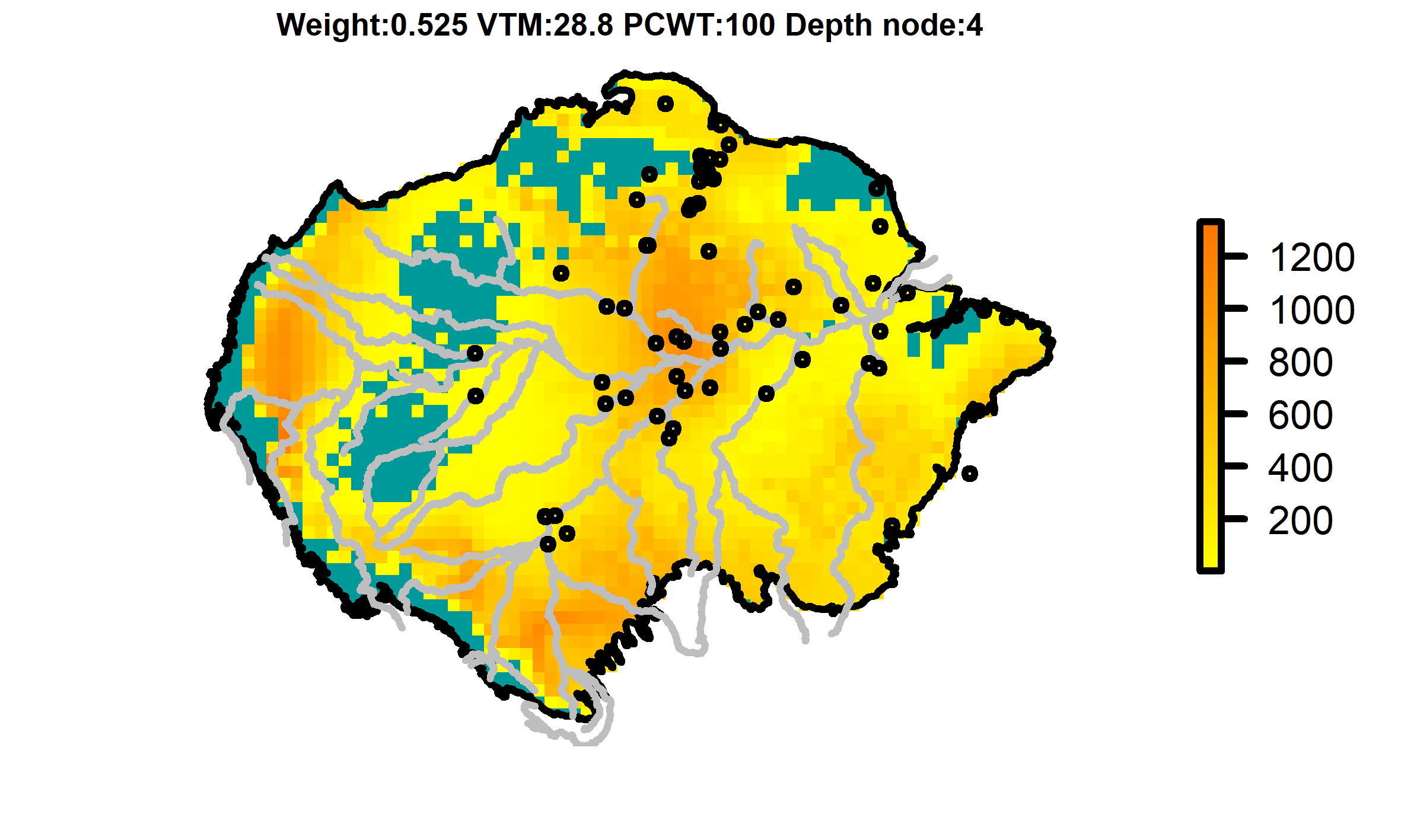

Supplement: S1 Data — (ZIP) [file pone.0286502.s002.zip › maps/map 61.png]

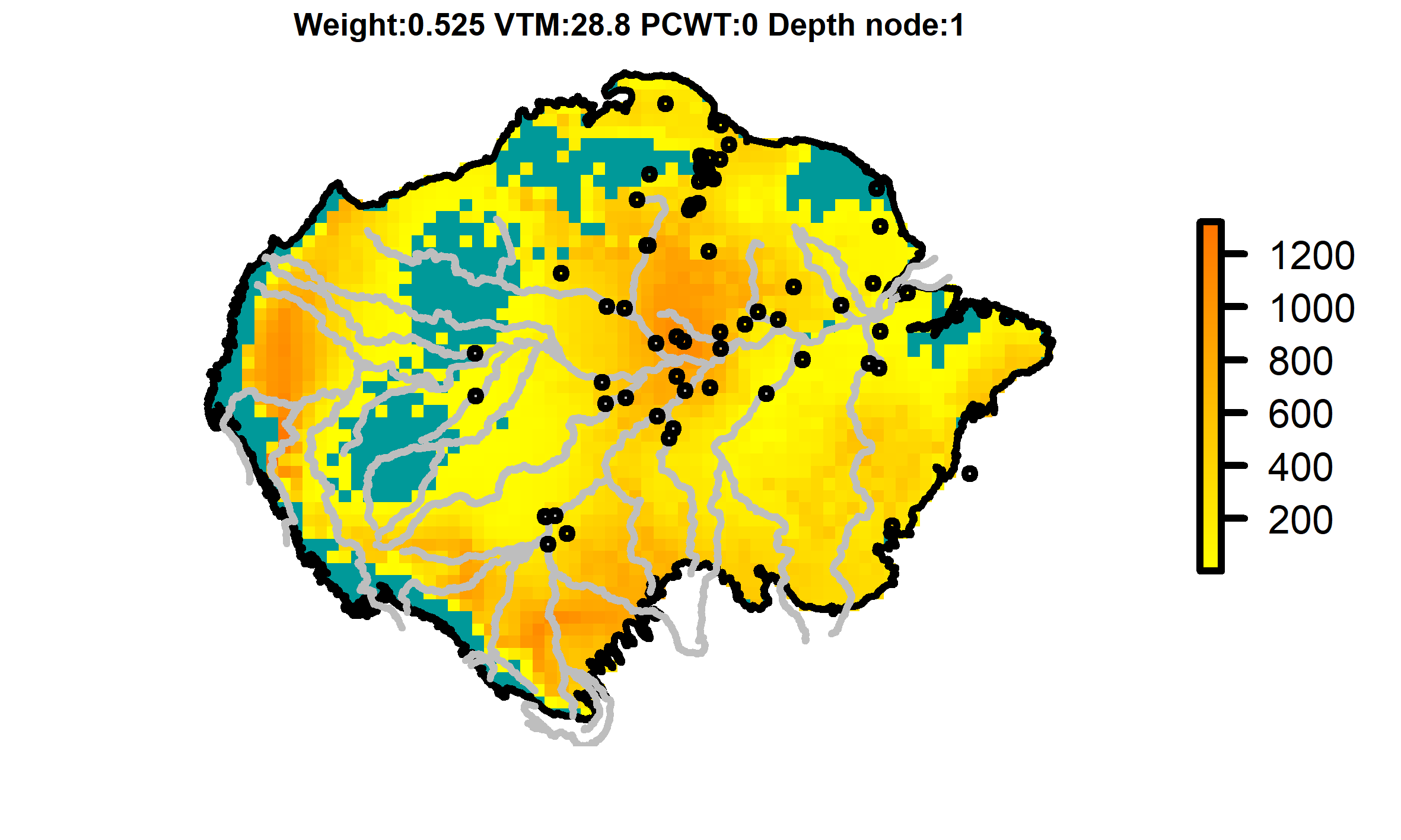

Supplement: S1 Data — (ZIP) [file pone.0286502.s002.zip › maps/map 49.png]

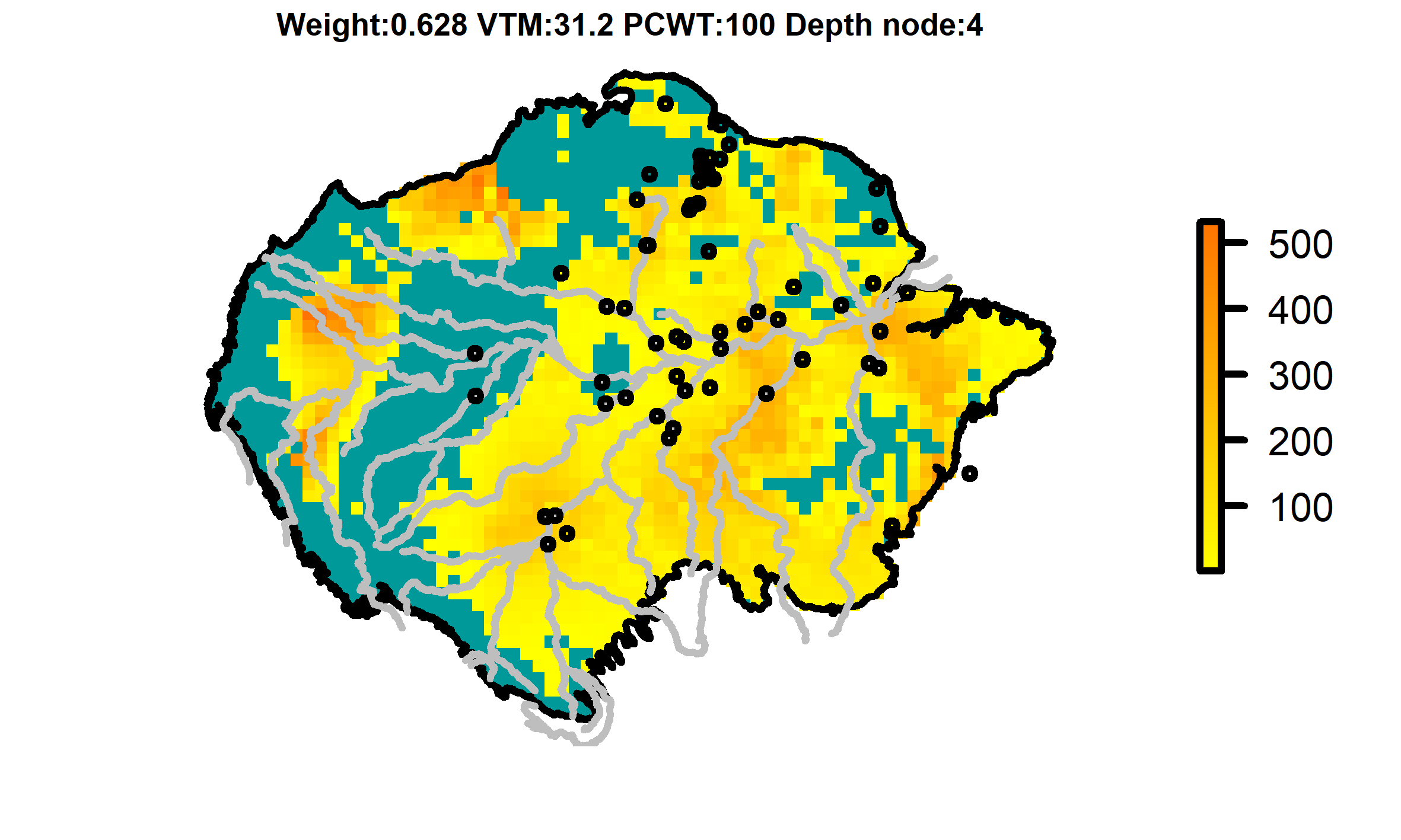

Supplement: S1 Data — (ZIP) [file pone.0286502.s002.zip › maps/map 48.png]

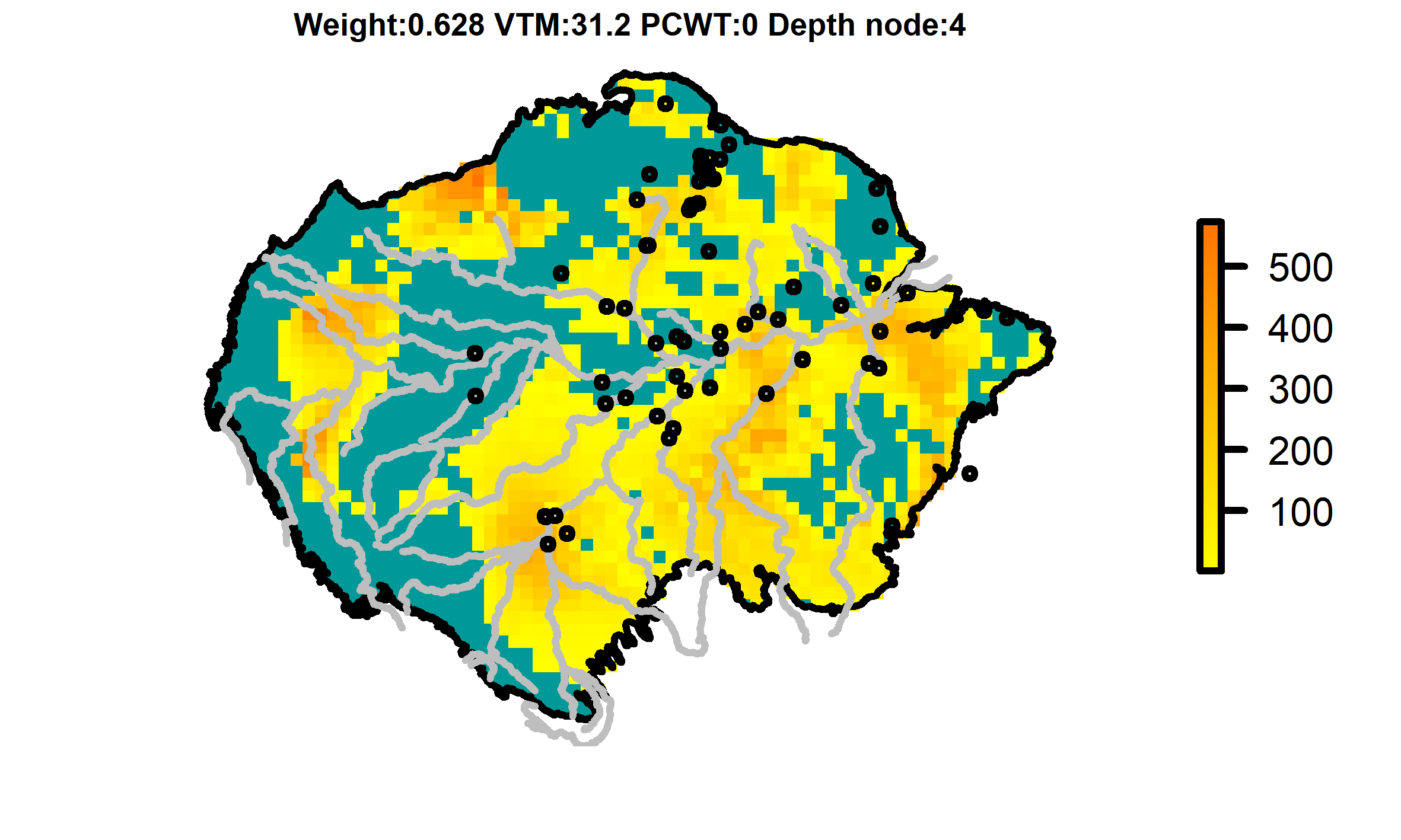

Supplement: S1 Data — (ZIP) [file pone.0286502.s002.zip › maps/map 60.png]

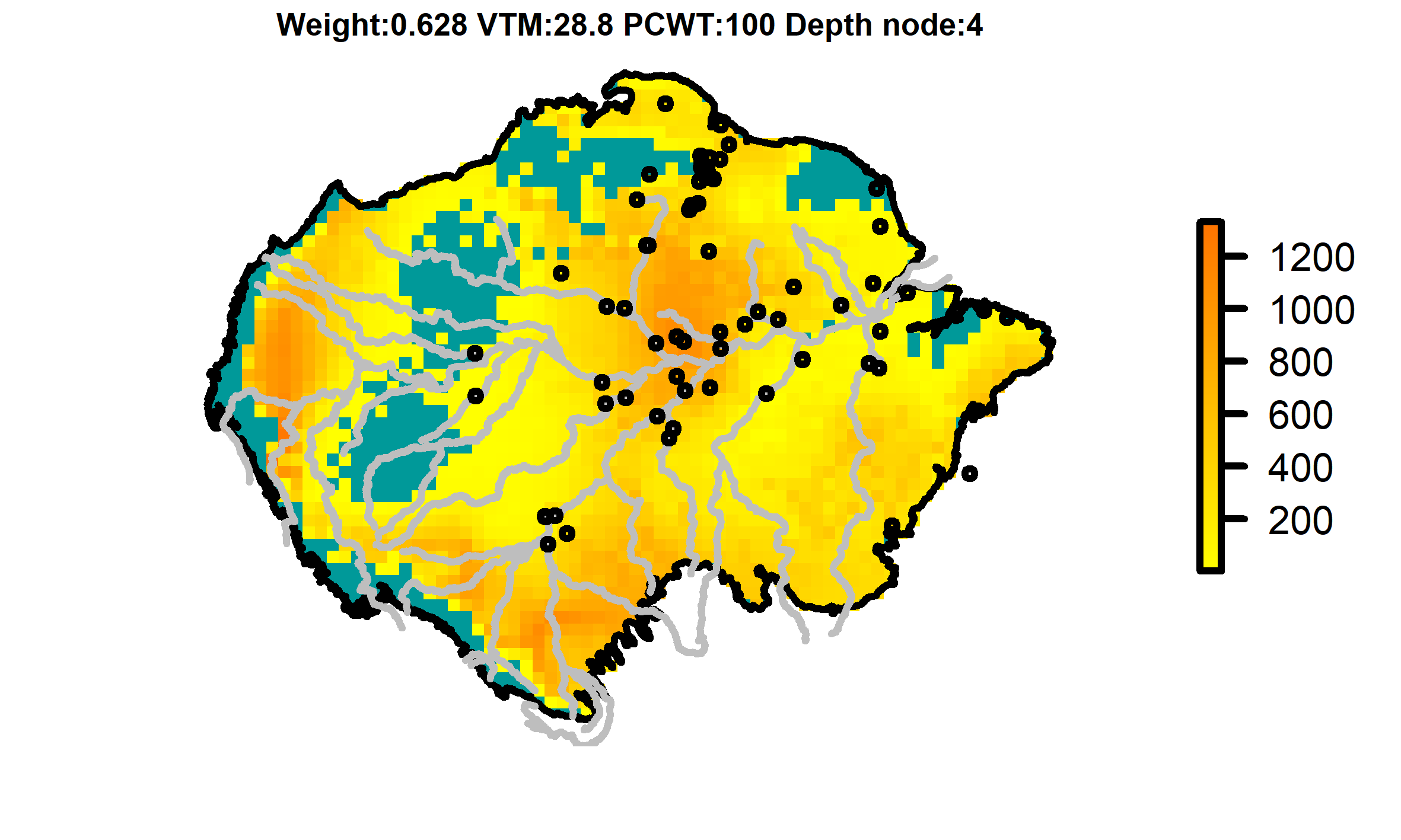

Supplement: S1 Data — (ZIP) [file pone.0286502.s002.zip › maps/map 62.png]

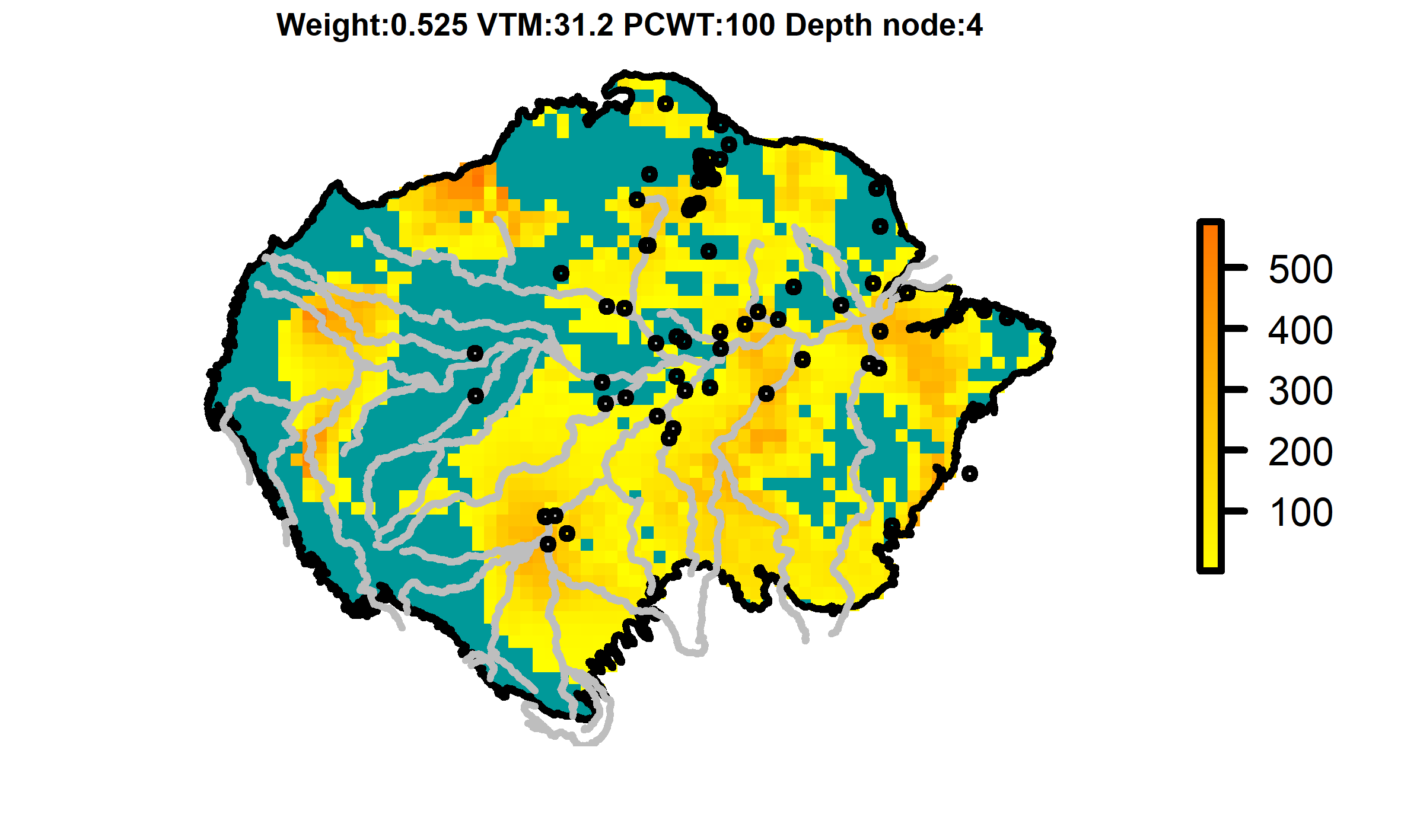

Supplement: S1 Data — (ZIP) [file pone.0286502.s002.zip › maps/map 63.png]
