# Supplementary material for: Dehydration alters behavioral thermoregulation and the geography of climatic vulnerability in two Amazonian lizards
Source: PLoS One. 2023 Nov 1;18(11):e0286502. doi: 10.1371/journal.pone.0286502 (PMC10619801; doi:10.1371/journal.pone.0286502)
Supplement: Attachment — Submitted filename: answers to editorial comments.docx [file pone.0286502.s003.docx]

PONE-D-21-29853

Dehydration alters behavioral thermoregulation and the geography of climatic vulnerability in Amazonian lizards.

PLOS ONE

Dear Dr. Camacho,

Thank you for submitting your manuscript to PLOS ONE. After careful consideration, we feel that it has merit but does not fully meet PLOS ONE’s publication criteria as it currently stands. Therefore, we invite you to submit a revised version of the manuscript that addresses the points raised during the review process.

Please do take the time to give thoughtful consideration to the comments provided by the reviewers here. If substantial revisions are not made with respect to these comments, the manuscript will not be accepted. As you can see, one reviewer feels that previous reviews were not taken seriously.

Please submit your revised manuscript by May 20 2022 11:59PM. If you will need more time than this to complete your revisions, please reply to this message or contact the journal office at [plosone@plos.org](mailto:plosone@plos.org). When you're ready to submit your revision, log on to <https://www.editorialmanager.com/pone/> and select the 'Submissions Needing Revision' folder to locate your manuscript file.

Please include the following items when submitting your revised manuscript:

- A rebuttal letter that responds to each point raised by the academic editor and reviewer(s). You should upload this letter as a separate file labeled 'Response to Reviewers'.
- A marked-up copy of your manuscript that highlights changes made to the original version. You should upload this as a separate file labeled 'Revised Manuscript with Track Changes'.
- An unmarked version of your revised paper without tracked changes. You should upload this as a separate file labeled 'Manuscript'.

If you would like to make changes to your financial disclosure, please include your updated statement in your cover letter. Guidelines for resubmitting your figure files are available below the reviewer comments at the end of this letter.

If applicable, we recommend that you deposit your laboratory protocols in [protocols.io](http://protocols.io/) to enhance the reproducibility of your results. Protocols.io assigns your protocol its own identifier (DOI) so that it can be cited independently in the future. For instructions see: <https://journals.plos.org/plosone/s/submission-guidelines#loc-laboratory-protocols>. Additionally, PLOS ONE offers an option for publishing peer-reviewed Lab Protocol articles, which describe protocols hosted on [protocols.io](http://protocols.io/). Read more information on sharing protocols at <https://plos.org/protocols?utm_medium=editorial-email&utm_source=authorletters&utm_campaign=protocols>.

We look forward to receiving your revised manuscript.

Kind regards,

Michael Sears, PhD
Academic Editor
PLOS ONE

Journal Requirements:

When submitting your revision, we need you to address these additional requirements.

1. Please ensure that your manuscript meets PLOS ONE's style requirements, including those for file naming. The PLOS ONE style templates can be found at 
<https://journals.plos.org/plosone/s/file?id=wjVg/PLOSOne_formatting_sample_main_body.pdf> and 
<https://journals.plos.org/plosone/s/file?id=ba62/PLOSOne_formatting_sample_title_authors_affiliations.pdf>

2. In your Methods section, please include a comment about the state of the animals following this research. Were they released, euthanized or housed for use in further research? If any animals were sacrificed by the authors, please include the method of euthanasia and describe any efforts that were undertaken to reduce animal suffering.

**Authors: we included:** After this study, the animals were transferred to the responsible for another project on lizard genetics and taxonomy. **At line 944**

3. We note that the grant information you provided in the ‘Funding Information’ and ‘Financial Disclosure’ sections do not match.

When you resubmit, please ensure that you provide the correct grant numbers for the awards you received for your study in the ‘Funding Information’ section.

4. Thank you for stating the following in the Acknowledgments Section of your manuscript: "We are grateful to Camila Moreira, Ivan Prates, José Cassimiro, José Mário Ghellere, Marco A. de Sena, Renato Recoder, and Sergio Marques-Souza for helping at the Rio Negro’s expedition in 2018. Kátia Pellegrino, Camila Moreira e a Gabriela Farias for providing ploidy level data. Sergio Marques-Souza identified the species. Marco Antônio Marques de Souza assembled the solar energy system used to allow experiments to be carried out on the boat. This work was supported by the Fundação de Amparo à Pesquisa do Estado de São Paulo (FAPESP), processes 2016/03146-4 (TOB) and 2011/50146-6 (MTR); Conselho Nacional de Desenvolvimento Científico e Tecnológico (CNPq), process 301778/2015-9 (MTR); Coordenação de Aperfeiçoamento de Pessoal de Nível Superior/Programa Nacional de Pós Doutorado (CAPES/PNPD) fellowship #code: 0001 and Marie Curie Grant (897901) (ACG)."

We note that you have provided funding information that is not currently declared in your Funding Statement. However, funding information should not appear in the Acknowledgments section or other areas of your manuscript. We will only publish funding information present in the Funding Statement section of the online submission form. 
Please remove any funding-related text from the manuscript and let us know how you would like to update your Funding Statement. Currently, your Funding Statement reads as follows:

"Agustín Camacho:CAPES/PNPD 001. MSCA:897901
Tuliana O. Brunes: FAPESP 2016/03146-4 
Miguel Trefaut Rodrigues: FAPESP: 2011/50146-6. CNPQ: 301778/2015-9.   
The funders had no role in study design, data collection and analysis, decision to publish, or preparation of the manuscript."

**Authors: It is ok as it is now.**

5. Please note that in order to use the direct billing option the corresponding author must be affiliated with the chosen institute. Please either amend your manuscript to change the affiliation or corresponding author, or email us at [plosone@plos.org](mailto:plosone@plos.org) with a request to remove this option.

6. We note that Figure 3 in your submission contain map images which may be copyrighted. All PLOS content is published under the Creative Commons Attribution License (CC BY 4.0), which means that the manuscript, images, and Supporting Information files will be freely available online, and any third party is permitted to access, download, copy, distribute, and use these materials in any way, even commercially, with proper attribution. For these reasons, we cannot publish previously copyrighted maps or satellite images created using proprietary data, such as Google software (Google Maps, Street View, and Earth). For more information, see our copyright guidelines: <http://journals.plos.org/plosone/s/licenses-and-copyright>.

**Authors: The images were created by the first author.**
 
We require you to either (1) present written permission from the copyright holder to publish these figures specifically under the CC BY 4.0 license, or (2) remove the figures from your submission:
 
a. You may seek permission from the original copyright holder of Figure 3 to publish the content specifically under the CC BY 4.0 license.  
 
We recommend that you contact the original copyright holder with the Content Permission Form (<http://journals.plos.org/plosone/s/file?id=7c09/content-permission-form.pdf>) and the following text:
“I request permission for the open-access journal PLOS ONE to publish XXX under the Creative Commons Attribution License (CCAL) CC BY 4.0 (<http://creativecommons.org/licenses/by/4.0/>). Please be aware that this license allows unrestricted use and distribution, even commercially, by third parties. Please reply and provide explicit written permission to publish XXX under a CC BY license and complete the attached form.”
 
Please upload the completed Content Permission Form or other proof of granted permissions as an "Other" file with your submission.
 
In the figure caption of the copyrighted figure, please include the following text: “Reprinted from [ref] under a CC BY license, with permission from [name of publisher], original copyright [original copyright year].”

**Authors: The images were created by the first author.**
 
b. If you are unable to obtain permission from the original copyright holder to publish these figures under the CC BY 4.0 license or if the copyright holder’s requirements are incompatible with the CC BY 4.0 license, please either i) remove the figure or ii) supply a replacement figure that complies with the CC BY 4.0 license. Please check copyright information on all replacement figures and update the figure caption with source information. If applicable, please specify in the figure caption text when a figure is similar but not identical to the original image and is therefore for illustrative purposes only.
The following resources for replacing copyrighted map figures may be helpful:
 
USGS National Map Viewer (public domain): <http://viewer.nationalmap.gov/viewer/>
The Gateway to Astronaut Photography of Earth (public domain): <http://eol.jsc.nasa.gov/sseop/clickmap/>
Maps at the CIA (public domain): <https://www.cia.gov/library/publications/the-world-factbook/index.html> and <https://www.cia.gov/library/publications/cia-maps-publications/index.html>
NASA Earth Observatory (public domain): <http://earthobservatory.nasa.gov/>
Landsat: <http://landsat.visibleearth.nasa.gov/>
USGS EROS (Earth Resources Observatory and Science (EROS) Center) (public domain): [http://eros.usgs.gov/#](http://eros.usgs.gov/)
Natural Earth (public domain): <http://www.naturalearthdata.com/>

7. We note that Figure 1 in your submission contain copyrighted images. All PLOS content is published under the Creative Commons Attribution License (CC BY 4.0), which means that the manuscript, images, and Supporting Information files will be freely available online, and any third party is permitted to access, download, copy, distribute, and use these materials in any way, even commercially, with proper attribution. For more information, see our copyright guidelines: <http://journals.plos.org/plosone/s/licenses-and-copyright>.
 
We require you to either (1) present written permission from the copyright holder to publish these figures specifically under the CC BY 4.0 license, or (2) remove the figures from your submission:
 
a. You may seek permission from the original copyright holder of Figure 1 to publish the content specifically under the CC BY 4.0 license. 
 
We recommend that you contact the original copyright holder with the Content Permission Form (<http://journals.plos.org/plosone/s/file?id=7c09/content-permission-form.pdf>) and the following text:
“I request permission for the open-access journal PLOS ONE to publish XXX under the Creative Commons Attribution License (CCAL) CC BY 4.0 (<http://creativecommons.org/licenses/by/4.0/>). Please be aware that this license allows unrestricted use and distribution, even commercially, by third parties. Please reply and provide explicit written permission to publish XXX under a CC BY license and complete the attached form.”
 
Please upload the completed Content Permission Form or other proof of granted permissions as an "Other" file with your submission.

**Authors: we own the copyright of the original images, and in any case we removed them as requested by one reviewer. Fig1 is now a graph created by the first author.**

In the figure caption of the copyrighted figure, please include the following text: “Reprinted from [ref] under a CC BY license, with permission from [name of publisher], original copyright [original copyright year].”
 
b. If you are unable to obtain permission from the original copyright holder to publish these figures under the CC BY 4.0 license or if the copyright holder’s requirements are incompatible with the CC BY 4.0 license, please either i) remove the figure or ii) supply a replacement figure that complies with the CC BY 4.0 license. Please check copyright information on all replacement figures and update the figure caption with source information. If applicable, please specify in the figure caption text when a figure is similar but not identical to the original image and is therefore for illustrative purposes only.

8. Please include captions for your Supporting Information files at the end of your manuscript, and update any in-text citations to match accordingly. Please see our Supporting Information guidelines for more information: <http://journals.plos.org/plosone/s/supporting-information>.

[Note: HTML markup is below. Please do not edit.]

Reviewers' comments:

Reviewer's Responses to Questions

**Comments to the Author**

1. Is the manuscript technically sound, and do the data support the conclusions?

The manuscript must describe a technically sound piece of scientific research with data that supports the conclusions. Experiments must have been conducted rigorously, with appropriate controls, replication, and sample sizes. The conclusions must be drawn appropriately based on the data presented.

Reviewer #1: Partly

Reviewer #2: Partly

2. Has the statistical analysis been performed appropriately and rigorously?

Reviewer #1: Yes

Reviewer #2: Yes

3. Have the authors made all data underlying the findings in their manuscript fully available?

The [PLOS Data policy](http://www.plosone.org/static/policies.action#sharing) requires authors to make all data underlying the findings described in their manuscript fully available without restriction, with rare exception (please refer to the Data Availability Statement in the manuscript PDF file). The data should be provided as part of the manuscript or its supporting information, or deposited to a public repository. For example, in addition to summary statistics, the data points behind means, medians and variance measures should be available. If there are restrictions on publicly sharing data—e.g. participant privacy or use of data from a third party—those must be specified.

Reviewer #1: No

Reviewer #2: Yes

4. Is the manuscript presented in an intelligible fashion and written in standard English?

PLOS ONE does not copyedit accepted manuscripts, so the language in submitted articles must be clear, correct, and unambiguous. Any typographical or grammatical errors should be corrected at revision, so please note any specific errors here.

Reviewer #1: No

Reviewer #2: No

 Reviewer #1: I already had the chance to review this manuscript when submitted to another journal, which unfortunately later rejected it. The aspect that strikes and saddens me the most is the number of comments I originally made that were wholly ignored. Indeed, most of my original comments had to be repeated in the present version. This means that the authors did not make any effort to improve their manuscript after the first rejection and merely resubmitted the manuscript somewhere else.

Hence, the authors disrespected a colleague who devoted various working hours to improve their manuscript. Now I discover that I wasted those hours since the authors ignored all of my suggestions. It is lousy and bad practice in science to ignore a reviewer's comments only because a journal rejected your manuscript. You could have submitted a much better version to PlosOne, instead of forcing me to waste even more time to repeat all my comments and force other reviewers to go through your original version. I sincerely hope that you will learn a lesson from this experience: there are not so many reviewers out there, after all, and your manuscript may end up in the hand of the same person again. Hence, revise your manuscript regardless of the final decision from the journal, even more so if it was rejected. Take advantage and acknowledge the amount of (free) work a reviewer devoted to trying to improve your work.
That said, and not willing to spend the same amount of time I invested on the first revision. The authors are not native speakers, and it shows. As for the first revision, I suggest many corrections (typically where the errors are painful to read), but I do not revise their English. Instead, I strongly advise an English revision by a native speaker, which has not been done. I'll also copy-paste all comments still valid.

**Authors: Although we disagree with reviewer1’s main criticisms, which seem to derive from a misunderstanding of this work’s objective or methods, we followed his/her suggestions whenever we could. We did not want to disrespect anyone, but given that we oppose the main criticisms and the pressure for the mounting work, we decided to submit it to PlosOne, including only some changes that seemed most important. This time, we made large changes to the text to improve its clarity and avoid the same situation.**

Original major comments
As it stands, the manuscript needs a complete revision, and even after one is carried out, I believe it to be of more modest reach than the authors may feel it to be.

For starting, the English need a profound revision. Many grammar errors are present, and several sentences are hard to understand. I rarely recommend such a revision; however, the English quality is not good enough for publication. I pointed out some of the passages most clearly needing a change, but I encourage a more thorough revision, possibly done by a native English speaker.

**Authors: the text was previously reviewed by a non-native English teacher and this time by two native british speakers.**

Second, the sample size was small and unbalanced: one of the two species used was represented by less than ten individuals, while for the other, 19 individuals were tested. I can understand the difficulties in collecting data while working on a boat in the Amazonas. However, the limitations on statistical and biological reaching of a small sample size remain, no matter how hard it was to get the data. Hence, the reader must be cautioned about the inherent problems of small sample sizes, like the increased uncertainty of all estimates. Authors must account for this aspect, both in the result and the discussion.

**Authors: To account for the different challenges of small sample sizes during analysis, we conducted a rigorous test using individuals from two species to test the hypothesis: the VTmax is altered by hydration level in lizards, which is stated as the main objective of this manuscript. We used three repeated measures per individual, whose hydration level was experimentally manipulated. Our sample size is indeed lower for L. ferreira (9inds*3 trials =27) than for L. percarinatum (19*3=57), but we reached 80 observations to test our hypothesis and we are not comparing the two species. Instead, they enter as random grouping factor in our analysis (see methods).**

**Our version sent to PlosOne also included the following explanation in the methods: “**This repeated-measures procedure has been validated to detect variations below 1ºC in the VTMAX of lizards with similar sample sizes[28]. Repeated-measures designs have increased statistical power that allow using relatively small samples[60] [28,60].” **For the initial version sent to Plos one, we also included a formal analysis of statistical power for our samples, which further indicated that it reaches sufficient power level. This was made explicit in the methods and results: ex. “**To avoid concerns with the statistical power of our analyses, we added a formal power analysis for each model in the model selection procedure. Power analysis identifies the probability of correctly rejecting the null hypothesis [62].”**. Even though, we now also included the following disclaimer: “**the sampling sizes could be considered somewhat low. However, given the changes in VTmax observed independently in almost every experimentally treated individual, in two different species, leaves us confident that the observed effects are real**”.**

**Besides, our samples are indeed balanced (i.e. the compared treatments have identical sample sizes). We made explicit from the beginning that we are not comparing species in our tests, but the same individuals under different treatments. Thus, we also reject the criticism about unbalanced sampling.**

Third, having a small sample size and a single population affect the mapping attempt. I applaud the effort to draw a map of overheating risk. Still, I have serious reserves that any significant conclusion could be drawn using just a few individuals from a single locality.

**Authors: this is factually wrong. We declared the number of sites collected and their locations were provided in the supplementary material. We never did a map for *L. ferreirai,* the endemic species for which we have less sites.**

“Thus, most *L. ferreirai* come from *Igapós* near to Boa Vista. *Igapós* are forests typical from Amazonian rivers, which experience seasonal floods. In turn, *L. percarinatum* were collected at Boa Vista, at *terra firme* forest, within the *Parque da Neblina* National Park, and at four sites in the west margins of the Rio Negro, across 500 km from the park to Novo Airão (see georeferenced localities in the supplementary file).”

**Besides, we always stated that our maps were for *L. percarinatum*, the species from which we have collected data from several sites**.

Specific comments (mostly repeated)

Abstract
L11: I would suggest using "hydro-thermoregulation" instead of "thermohydroregulate" throughout the manuscript. In my opinion, it would facilitate the reading. (Repeated)

**Authors: Thermohydroregulation is a relatively new term, but already used in the literature. Searching for: “thermohydroregulation” gave about 39 results, some of which we cite.**

**Following the reviewer’s opinion, we made a search in Google scholar for hydro-thermoregulation. It rendered one result: [CITATION] Mechanisms~/Age# ig and De, elopment. 36 l 1986) 303-306 303 Elsevier Scientific Publishers Ireland Ltd.**

**Thus, we would wish to keep the option most acknowledged in the scientific literature and in the literature that we cite.**

L14: Replace "VTMAX". "VTmax".

**Authors: We exchanged VTMAX by VTmax. It is also called: VTM, Tvol, behavioral responses, and other terms in the scientific literature.**

L20: "which also reacted to start temperature and heating rates" is not clear. Please rephrase. (Repeated)

**Authors: we rephrased to**:**”**and also changed with start temperature and heating rates”.

L21: remove the comma after "dehydration". (Repeated)

**Authors: There was no such comma in the submitted version**.

L23: remove the comma after "risk". (Repeated)
**Authors: removed.**

Introduction
L32: "thermophysiological" sounds weird. I suggest removing the word altogether since later on in the sentence, the authors explain the thermal dependence of performance. (Repeated)

**Authors: searching for the term “thermophysiological” rendered 7.790 results in google scholar. Thus, we would wish to keep it, to remain in conformity with previous literature.**

L33: replace "Ex." with "e.g." throughout the text. (Repeated)

**Authors: Although we changed them, there was one left in the submitted version to PlosOne, that we now corrected**.

L34: I would replace "Dehydrated animals (ex. herps), exhibit" with "Dehydrated ectotherms may exhibit". (Repeated)

**Authors: We modified it to “**Dehydrated amphibians and reptiles often exhibit..”

L35: "the high temperature that blocks locomotion": do you mean to say something like "exhibit lower critical thermal maxima, the limit which, if surpassed, would impede locomotion"? (Repeated)

**Authors: Yes, we intended that and changed the text to the following explanation for CTmax : “**(i.e. a high body temperature which, if surpassed, would impede the individual’s locomotion [4–6])”

L37: I would be more precise than "animals", especially if you already refer to ectotherms. (Repeated)

**Authors: We rephrased to: “**Many ectothermic animals,”

L40: the content of the brackets is odd: mammals is an order, chicken a family, and then two lizards species are called by their Latin names. Please rephrase it. (Repeated)

**Authors: We removed all the text about endotherms due to the criticisms of both reviewers.**

L42: "herp" is a colloquial term and should not be used in a scientific article. Please remove it from the text. (Repeated)

**Authors: we changed it to “amphibians and reptiles”.**

L47: "in" instead of "of". (Repeated)

**Authors: nor the two native speakers or the Grammarly app found any problem with of.**

L48: remove the comma after "both". (Repeated)

**Authors: removed.**

L49: place here the abbreviation VTmax. (Repeated)

**Authors: changed.**

L49: "a" instead of "one". (Repeated)

**Authors: changed.**

L56: "Might be overwhelmed" instead of "might have these limits overwhelmed". (Repeated)

**Authors: we left it as “**Also, individuals not perfectly sheltered might be overwhelmed”.

L56: add "-" after "inter". (Repeated)

**Authors: “interspecific” is correct according to the Collins dictionary.**

L61: add "in" before "the lizard". (Repeated)

**Authors: Added.**

L67: Add "By" before "combining". (Repeated)

**Authors: Added.**

L73: "Air temperature" instead of "The temperature of the air". (Repeated)

**Authors: Changed**

L72: I disagree with this statement. Most small body-sized ectotherms, like lizards, can easily escape into burrows, crevices and under rocks of various sizes to escape hot temperatures. Hence, air temperature in the shade is by no means the minimum temperature they have available. (Repeated)

**Authors: We precisely intended to account for thar in original manuscript**: ”small ectotherms might still bury within moist microhabitats and experience lower maximum temperatures[32], making it important to evaluate how changes in thermal tolerance (Ex. in the VTMAX), and in the availability of such microhabitats, might alter our estimations of climatic vulnerability."

L84: Provide references for the statement: "determines the availability of moisture and water for animals, potentially affecting dehydration rates, thermal tolerance, and the risk of exposure to extreme environmental temperatures".

**Authors: we added the following reference:** Davis, J. R., & DeNardo, D. F. (2010). Seasonal patterns of body condition, hydration state, and activity of Gila monsters (Heloderma suspectum) at a Sonoran Desert site. *Journal of Herpetology*, *44*(1), 83-93.

**Results**
L104: "starting" instead of "start".

**Authors: we changed that too.**

L104: rankit should be italicized throughout the text. (Repeated)

**Authors: changed.**

Discussion
L157: Add "-"after "intra". (Repeated)

**Authors: for the reason stated above, we did not adopt this suggestion.**

L158: "of" instead of "in". (Repeated)

**Authors: this sentence is no longer in the article.**

L159: This concept appears of the blue here for the first time. Briefly explain what do you mean by "anhomeostasis", instead of merely quoting a paper. This is the discussion section, after all. (Repeated).

**Authors: We exchanged anhomeostasis for its own definition and rephrased:** “… except in the rare cases when individuals let their inner environment fluctuate largely [38].”

L160: Remove the comma after "such as". (Repeated)

**Authors: removed**

L171: "locomotion" instead of "speed"

**Authors: changed**

L176: "research on" instead of "documentation of"

**Authors: changed**

L179: "since active-thermoregulators are often found at them" is not clear. Please rephrase. (Repeated)

**Authors: We removed this part**

L183-184: The quote "20" does say the exact opposite of the authors. From the abstract of Sannolo et al. 2020, it can be read the dehydration lowered preferred body temperatures in all lizards tested. Please rephrase. (Repeated)

**Authors: Sannolo et al are in reference 18, not 20. Their study show an effect on mean preferred temperatures, but the maximum temperatures accepted in the gradient, which are more comparable to a voluntary thermal maximum, remained at the same levels, something similar happens in le galliard’s study of *Zootoca vivipara*, now added to the manuscript, too. Thus, we rephrased to “**Experimental dehydration did not lower the VTmax of the desert iguana [12], nor the maximum body temperatures of several temperate lacertidae species, studied in thermal gradients[15,18,21].”

L184-185: the change in VTmax in the desert iguana is not due to dehydration since the water content did not change. Instead, after the injection, the Iguana suffered from electrolyte imbalance, which may influence the thermal parameters. Please rephrase. (Repeated)

**Authors: we rephrased to “**However, desert iguanas did alter their VTmax in response to injections of concentrated saline solutions, leading to osmotic imbalance”

L190: Discussing panting is out of topic, as it seems a clumsy attempt to increase auto-citation. Please delete this sentence. (Repeated)

**Authors: Panting is a behavior that combines thermal tolerance with water loss and is being used as a different behavioral measure of thermal tolerance. Thus, we believe it is appropriate here. Likewise, our review of the use of such trait across lizards is as relevant as early measurements of this parameter, which were also cited. We similarly acknowledged other reviews within topic (e.g., Tattersall`s, Pirtel’s, Curry-lindhal’s) throughout the manuscript.**

L192: "Keeping the VTM up might help lizards (and other organisms)" is colloquial. Please rephrase. (Repeated)

**Authors: we rephrased to** “Maintaining a high VTmax might help lizards (and other organisms) behave more flexibly”.

L193: "maintaining": do you mean retaining?

**Authors: we rephrased to** “This could allow them to maintain feeding activity and higher metabolic rate, with the resultant ingestion of water in the food and enhanced metabolic water production”

L194: "keeping physiological performance high" is colloquial. Please rephrase. (Repeated)

**Authors: this does not appear in our submitted manuscript to PlosOne.**

L202-209: This paragraph on the differences between endotherms and ectotherms should be removed altogether. The differences between these two groups are so profound in any aspect considered (and run so deep in the phylogeny) that there is no point in comparing them to advocate for obvious differences in body size or water balance. (Repeated)

**Authors: We deleted the reference to endotherms.**

L210: This sentence is colloquial (define "high" and subject species), and no references are provided. Please rephrase and provide evidence or remove it. (Repeated).

**Authors: we rephrased to: “**Further, maintaining high body temperatures might be especially important for species living at places with short seasons for growth and reproduction (E.g. deserts, temperate regions, [38,42,49]”

L210: "in" instead of "at". (Repeated)

**Authors: we rephrased to “**Further, maintaining high body temperatures might be relevant for species living in places with short seasons for growth and reproduction”

L212: "in" instead of "at". (Repeated)

**Authors: changed.**

L213: "inside a" instead of "at a"

**Authors: changed**

L210-230 and 239-259: These whole paragraphs deal with specific details of the results. Hence, it should be moved at the very beginning of the discussion. The discussion section should flow from the details to the general debate, not the other way around.

**Authors: We started the discussion with the main result, and then followed with secondary specific results. These lines 210-230 deal with implications of our experimental results for the understanding of effects of hydration on the VTmax. Thus, we would like to avoid starting by that part and keeping the structure as it is. The other reviewer did not see any problem with the structure of this manuscript’s discussion.**

Methods
L287: remove "as collected". (Repeated)

**Authors: removed**

L301: "Brought back" instead of "taken back". (Repeated)

**Authors: changed**

L302: Was this statement tested or just assumed? If tested, please provide numbers. (Repeated)

**Authors: As shown in that statement, it was observed that sometimes rehydrated lizards reached higher hydration levels, compared to recently captured ones. Thus, we did not test nor assumed a full hydration level. Using 100% for the most hydrated state of each individual allows testing for an effect of dehydration without knowing the fully hydrated state of each animal. Also, we added that: “**To avoid the assumption that freshly captured lizards, or rehydrated lizards, were at their fully hydrated state, we simply considered their maximum weight obtained across the three measurements, as the fully hydrated weight. ”**the numbers (weights) are provided in the supplementary material.**

L305: Using the maximum weight is an assumption as well, since (unfortunately) there is no way to know for sure which weight represents the 100% hydrated condition. (Repeated)

**Authors: we added: “**This simplification, combined with comparing each individual with itself along the three turns, allows to identify whether higher hydration levels lead to exiting the heating chambers at hotter temperatures without actually knowing the fully hydrated state of each one.”

L308: The term "turn" is confusing. I suggest using a more experimental wording, like "condition" or "trial". (Repeated)

**Authors: we changed turn by trial.**

L364: I do not see how body size is difficult to control experimentally in field studies. (Repeated)

**Authors: we did not argue that in our submitted manuscript to PlosOne. Instead we stated that it varies naturally and that we accounted for such natural variation in our analyses.**

344: Please italicize function names. (Repeated)

**Authors: italicized**

347: Please provide numbers for "slight deviations". (Repeated)

**Authors: provided**

381: This first sentence of the paragraph is superfluous: state directly what you aim to achieve, not what you don't want to. (Repeated)

**Authors: Despite this first sentence disclaimer, reviewer 2 was still concerned that our maps could be more than just illustrative. This makes us believe that this disclaimer is particularly relevant. We tried to find a suitable solution to Rev1 and Rev2 requests by taking the disclaimer to the end of the paragraph.**

Reviewer #2: This manuscript reports on the effect of mild dehydration on the temperatures voluntarily tolerated by two species of Amazonian lizards. Based on those experimental results, the manuscript also reports output of two models of how those thermoregulatory responses to dehydration might affect risk of exposure to thermally challenging conditions across the geographic range.
As the authors state, the interactions between hydration and thermoregulation are an area that needs additional study. I think that this study provides some valuable data, and an interesting perspective, but I have some concerns about some assumptions, and comments about presentation.

**Authors: we thank the patience and time of the reviewer. We followed almost all of the suggestions and edited the text to ease his/her concerns.**

The authors make a point in a couple of places (lines 53,396-7) that "exposure to it {VTMAX} can kill in a matter of hours", but that was for a species with VTMAX very high (42.5C) and close to CTmax (~43C),

**Authors: actually, apart from that one, other two lizard species, whose VTmax was 2 and 3 degrees below their respective CTmax also experienced deaths of individuals before 4h of exposure (see ref [28] in the manuscript). We explained this in the introduction [L70,80].**

which is very different from the species in this study (VTMAX ~24-33C for both species; CTM>38C), which have a buffer of 5-8 degrees before reaching lethal/dangerous temperatures.

This assumption that temps above VTmax are always dangerously high drives the results in the modeling analyses, and the conclusions that these species may be vulnerable under dryer and/or warmer conditions. Because of that large buffer, I'd like to see more justification that VTMax represents a dangerous threshold, or at least more discussion about its use as an illustration, rather than a realistic estimate of thermal vulnerability.

**Authors: Thanks, we did add more justification on that buffer distance and considerations for using the VTmax as a threshold [L 278-287]. Besides, we stated multiple times [104, 432, 447] across the text that our study and the maps are only illustrative.**

I was concerned about some assumptions in the modeling. In particular, the assumptions above, and the assumption that air temperature is the coolest temperature available, despite the models allowing for burrow use (soil temps can be cooler than air temps), and discussion in the manuscript about use evaporative cooling (panting, water loss from the surface, etc.). Because of this, it seems like the maps in Fig. 3 overstate the thermal risk of the species.

**Authors: we acknowledged the limitation of considering air temperature the minimum available [L 450-452] and actually applied a way to avoid the assumption that concerns reviewer2, which is using the Nichemapper, our second approach. The discussion goes about accounting for the animals ability to find a shelter and lower its maximum attainable temperatures [L292 and on]**

I found it hard to follow the justifications in the Intro and Discussion where temperate (or desert) species were used to justify ideas about low, wet tropical species. I think it is important to separate those more clearly in the text, because warm (sometimes arid) temperate locations create very different selective pressures than lowland wet tropics.

**Authors: We need to compare our data from rainforest lizards with estimates of the VTmax performed before, those are from other species, mostly lizards from deserts and temperate regions. In the last version, we tried to leave the messages as clear as possible.**

In some cases, the manuscript uses references to mammals and birds to support ideas related to CTmax and VTmax, which are typically used to describe ectotherms.

**Authors: We excerpted all the references to mammals and birds.**

The experimental data were good, but could be presented more effectively. Some results were relegated to supplemental figures (e.g. S1, Table S2). Lines 96-101, I wanted to see the percentage hydration levels to know how hydrated the animals were. It's more intuitively useful to see that they're 93% of maximum mass than knowing that they lost 0.075g when dehydrated. this made it very hard to know how dehydrated this setup made the animals.

**Authors: Yes, because of space limitations, but we changed the picture of the animal by Fig S1. Readers can actually see VTMax directly related to hydration percentage in Figure 2. Yet, as we explain in the methods, there is no easy way to know the actual hydration state of each individual. That would require measurements of the blood of a baseline of fully hydrated lizards. What we do and is easy to do is to rank them according to their lost or gained body water. We explained that in the methods L[353-363]**

Hydration level also helps to understand the magnitude of the effect of dehydration on VTMax. It's given (line 110) as 0.12C per percent hydration, but nowhere was that extrapolated to the total change (e.g. from 93%-100% represents about 1C change in VTMax). This is especially important when the authors point to a 20% difference in the effect between species (line 218), which seems to be only a fraction of a degree.

**Authors: The total change in VTMax was actually much larger (2.5-3C on average) across the total change in hydration level studied (~77-100%). The largest changes reached 6 degrees. It is hard to tell what is more important for the animal, absolute or the relative changes. We thus presented both aspects (L126-145 and Fig 2).**

Specific comments:
- define terms like: turn, start temperature, etc.

**Authors: Turn (now trial, attending the other reviewer) was originally defined in L406 and maintained in the new version. Starting temperature is now defined in L .60**

- Not sure what I was supposed to get from Table 1. Seems to be just a list of variables that effect the model output?

**Authors: This table does show the list of such parameters, but also the effects of changing these parameters on indices of thermal vulnerability. We explained that in the table’s legend and in the methods section L[486-489].**

- The intro seemed to jumble CTmax and VTmax. Would help to clearly separate these concepts in the Intro

**Authors: We did a few modifications. In sum, these measures are presented in two different paragraphs in the introduction section. We included amendments to try and make it clearer. L[34, 51]**

- Line 170-171; Pirtle et al. discusses behavioral alternatives to affect water loss, without thermoregulatory loss or energetic demand (Pirtle, Elia I., Christopher R. Tracy, and Michael Ray Kearney. "Hydroregulation: A neglected behavioral response of lizards to climate change?." Behavior of lizards. CRC Press, 2019. 343-374.)

**Authors: We added that reference and added this reference, too.**

**Le Galliard, J. F., Rozen-Rechels, D., Lecomte, A., Demay, C., Dupoué, A., & Meylan, S. (2021). Short-term changes in air humidity and water availability weakly constrain thermoregulation in a dry-skinned ectotherm. *Plos one*, *16*(2), e0247514.**

slines 202-207: This paper is primarily relevant to ectotherms, so this paragraph is unnecessary and can be deleted.

**Authors: we deleted that part.**

lines 231-237: I didn't see any discussion that justified these 6 characteristics. Please add justification for them.

**Authors: They are in there: Ex. trait 1: L221; trait 2: L695. Traits 3,4,5 and 6 arise from finding our species to have a VTmax highly reactive to dehydration, while the other studied lizards did not (Ex. L 225-230).**

lines 422-429: I think it's important to show that the CTM of this species is 6-10C above VTMax, and that the change in VTmax shown in the experiments is about 1C.

**Authors: We added that in L[762]. We do not know where is this 1C change coming from. We showed in Fig.2 that the average change is around 2.5 *for L percarinatum* and 3 for *L. Ferreirai* (look at model lines in figure 2).**

Check reference formatting.
Fig. 1. As much as I like seeing the study animal, this figure could be in the supplement.

**Authors: we changed it by our original Fig S1, which reviewer2 wanted to see in the manuscript.**

Fig. 2. It took a long time to see that there was a grey line that was the overall response. Very hard to read this figure, and to understand what it is supposed to show. Consider presenting only the summary line (maybe with confidence intervals?) in the main body, and show the individual responses in the supplement.

**Authors: In previous reviews of this manuscript, we have had different requests, including some who wanted the residuals and others that wanted the raw data, or the model line. To attend every perspective, we are showing both the raw data and the model expectations in the ms, and the residuals in the supporting material. We also showed the VTmax with respect to percentage hydration, which is precisely what reviewer 2 requested a few comments before this one. We tried to improve the figure and the legend, making the model line easier to see, and also plotted the confidence intervals based on the standard deviation, although they are so small that are hardly visible.**

Manuscript overall needs to be copy-edited by a native english speaker

**Authors: the text was copy-edited by two native speakers.**

6. PLOS authors have the option to publish the peer review history of their article ([what does this mean?](https://journals.plos.org/plosone/s/editorial-and-peer-review-process#loc-peer-review-history)). If published, this will include your full peer review and any attached files.


If you choose “no”, your identity will remain anonymous but your review may still be made public.


**Do you want your identity to be public for this peer review?** For information about this choice, including consent withdrawal, please see our [Privacy Policy](https://www.plos.org/privacy-policy).

Reviewer #1: No

Reviewer #2: No
