# Supplementary material for: Dehydration alters behavioral thermoregulation and the geography of climatic vulnerability in two Amazonian lizards
Source: PLoS One. 2023 Nov 1;18(11):e0286502. doi: 10.1371/journal.pone.0286502 (PMC10619801; doi:10.1371/journal.pone.0286502)
Supplement: Attachment — Submitted filename: second response to reviewers.docx [file pone.0286502.s004.docx]

Reviewer #2: General comments: This manuscript is much improved in some ways, and still needs some work in others. I found that most of my concerns about the content had been addressed, and therefore the science presented in the manuscript is much easier to evaluate and seems to meet reasonable publication expectations. There are still a number of issues with language, some of which make it hard to understand what the authors are trying to say. These seem fixable, if the authors work more closely with someone experienced with scientific writing (especially in biology) in English (preferably a native speaker).

-Lit Cited needs copy editing for formatting (e.g. species names not italicized, strange capitalization in titles)

**A: references are now corrected.**

Line 20: “and also changed VTmax(?) Systematically”

**A: added.**

Line 32: “despite it being”

**A: changed**

Line 37: metabolic costs do what?

**A: Rise, clarified in text**

Line 39: “use behaviors that integrate temperature…”

**A: changed**

Line 46: “associated with”

**A: changed**
Line 47: hydration levels (instead of water levels)

**A: changed**
Line 53: “temperature forces” and “seek cooler temperatures”

**A: changed the first but opted to keep retreat, as in the original citation and to avoid repetition.**

Line 56: no italics on Phrynosomatidae

**A: done**

Line 64: The Dupre paper was on desert iguanas, an active thermoregulator. Suggest stating the species name here, not referring to “desert lizards”, and pointing out that the species is not a thermoconformer. This sentence seems to fit better after the sentence on Hemiergis.

**A: Corrected. The study cited uses Sceloporus jarrovi and is not from Dupré. That sentence seems to be the conclusion of the paragraph, so we kept it in the end of the paragraph.**

Line 70: Like I said in my previous review, the species referenced here has a CTM of 43.0-43.9°C and at VTmax of 42.5°C… 0.5°C below CTM! If you’re going to use this extreme case as your example to show that exceeding VTmax is dangerous, you need to be transparent and honest about how close those critical temperatures are to each other for this example species. Many other species, including many listed in the table in the Curry-Lindahl paper, have this difference in the range of 3-5 °C. Additionally, table 4 in that paper lists many examples of species that live for 24-48 hours (or more) when held at temperatures above VTmax. You don’t need to overstate your case that being above VTmax may be stressful to convince readers, so just be honest about this, rather than picking only the most extreme data. Or if you insist on using the most extreme, you need to make it obvious that you are referring to the most extreme case you could find.

**A: The accusation of dishonesty here is unjustified. From the many examples pointed out by R2, we found only two unnoticed species that resisted over 24h in table 4 and are now added to the introduction text (L 86). Besides, according to the existing literature, the only extreme data here are these two species that the reviewer found. These two seem to be the exception, not the rule.**

**Besides, we had also already added that type of disclaimer at the discussion section.**

**“**The VTmax offers higher sensitivity to detect thermal stress than the thermal limits: some desert lizards may show one to three degrees between the VTmax and the CTmax [27,31], but *L. percarinatum* may reach to 5 [40]. However, interspecific variation in the magnitude of this “thermal fear zone”, and the lack of published data on it, might be seen as a problem to compare thermal risk among species.”

**And now added more discussion on this concern at L427.**

**“**Thus, we tentatively suggest that whichever site at which the minimum available temperatures (I.e., at species’ shelters) overcome the VTmax for more than half day should induce enough stress to make a population vulnerable. This should still be true even in the case of species particularly well adapted to stressful heat (see introduction). Processes arising from failed thermoregulation at suboptimal refuges, and the concentration of individuals at thermal refuges, dehydration, and negative intra and interspecific interactions deriving from such concentrations could likely harm the stressed population under the proposed situation.”

**Besides, CTmax is a far more geographically labile trait than VTmax, as data show in refs 28, 53, and in Clusella-Trullas, S., & Chown, S. L. (2014). Lizard thermal trait variation at multiple scales: a review. Journal of Comparative Physiology B, 184(1), 5-21.**

**We do not know where Reviewer 2 obtained the CTmax data he/she is writing about but it seems more reasonable for a species from open habitats. The following reference cites most values under 40 degrees (even some under 30), although it also shows a couple of values over 50, which are likely typographic errors. Still, the average CTmax for Loxopholis percarinatum, including the likely typos, goes to 38 degrees. In the personal experience of the first author, who measured the VTmax of this genus in other situations, some *Loxopholis* (former *Leposoma*) died at temperatures below 38, during assays.**

**Diele-Viegas, L. M., Vitt, L. J., Sinervo, B., Colli, G. R., Werneck, F. P., Miles, D. B., ... & Ávila-Pires, T. C. (2018). Thermal physiology of amazonian lizards (reptilia: Squamata). PLoS One, 13(3), e0192834.**

Line 94: use “dampens” not damps

**A: changed**

Line 101: Fig. 1 is a graph. Is there a figure missing?

**A: Thanks. The figure (and now the call to it) had been removed, as requested by another reviewer.**

Line 107: use “lizard weights”
**A: changed**

Note: I reviewed the Methods section before Results and Discussion because the latter are impossible to understand without first reading the Methods

**A: that seems appropriate.**

Line 310: replace period with comma

**A: done**

Line 311: “composed of leaf…”

**A:Changed, “By” is also correct.**

Line 318: “nine adult L. ferreirai”

**A: s removed**

Line 319: Which species?

**A:The latter, now clarified**

Line 328: “nor were observable differences attributable”

**A: done**

Line 330: “our results being”

**A: done**

Line 332: “data become”

**A: done**

Line 336: use “another” not more. Also add comma after dehydrated

**A: done**

Line 343, 344, 346, and many subsequent lines: I think recipients is supposed to be “chambers” or “boxes”? Recipients doesn’t make any sense.

**A: done**

Line 349: “allowed lizards to rehydrate to hydration levels”

**A: done**

I stopped copy-editing the English at this point (except where it affects meaning), but the rest of the Methods section has similar issues with wording and grammar that make it difficult to understand, and that do not meet expectations for clarity of writing in a manuscript acceptable for publication in PLOS ONE. I recommend again that the authors work more closely with someone experienced with scientific writing (especially in biology) in English, and preferably a native speaker.

**A: We rechecked grammar and again copyedited the text with the help of an American English native.**


Line 108: use “mass” instead of “weight” throughout

**A: done**

Line 109 and 110: use “difference from fresh mass”

**A: done**

Figure 2 legend (version under the figure): typo on “VTmaxax”, typo on “barrs”. Note – this figure is MUCH improved!

**A: corrected**

Line 127: Use “percentage” not percentual

**A: Percentual is the correct adjective of unit.**

Line 131: use “per degree/minute increase in heating rate”

Line 125 and 134: Both species had exactly the same range for VTmax? Both are given as 24.1 to 33.3C. This may be real, but is surprising, so I want to double check, especially because Fig. 2 suggests that VTmax may be higher in L. ferreirai than in L. percarinatum.

**A:Thank you for pointing that, it is now corrected. The rest of the results are checked and ok.**

Figure 3 legend (version under the figure): I’m not sure what is meant by “the subtraction of the median VTmax”. Is this just the difference between yearly max and VTmax (yearly max – VTmax)?

**A: it now reads: “**A and B represent the difference between the median VTmax of hydrated (A) or dehydrated (B) lizards and the mean of the yearly maximum air temperatures, at any pixel”

Reviewer #3: Manuscript PONE-D-21-29853R1 reports on original research regarding the responses of two species of thermoconformers lizards to dehydration and environmental changes at the local scale and the interaction between these variables. The study presents a map of thermal risks for one of the species in the Amazon Basin, an output of an integrative approach between thermohydroregulation and models of the geography of thermal risks. Although innovative, it constitutes preliminary research on the topic for both species. My main concern is how far the authors go to generalize their findings. Nonetheless, the methods used are appropriate, and the results partially support the conclusions. Therefore, I recommend this for publication after the authors attend to the following minor commentaries.

General comments:

I recognize the efforts to improve sampling and that the work is not comparing both species. However, the low geographic extent of the data and the number of sites and species analyzed precludes any conclusions from being generalized to all Amazonian lizards or inferring about places of greater thermal risk throughout the Amazon Basin. Therefore, I strongly suggest a readjustment in the tone of writing so the manuscript is more precise regarding its objectives and the extent of the results presented.

**A: We thank the reviewer but we did not add new sites/individuals. Besides, in no section of the article we proposed to extrapolate the results to “all Amazonian lizards” as the reviewer states. The manuscript already included three paragraphs (in the objectives, in the methods, and at the conclusion) addressing the concern stated by reviewer 3.**

**In the objectives**

**“**Later, we illustrated how changes in the VTmax, in the available soil depth for burying, in tree cover, and in precipitation, might alter the geography of perceived thermal risk for the more widely distributed *L. percarinatum,* across the Amazonian Basin.”

**In the methods**

“Herein we seek to simply illustrate how dehydration may alter geographic models of thermal risk. We generated these models based on the VTmax of hydrated and dehydrated lizards of L. percarinatum only, for which we had samples from multiple populations. We do not wish to provide a prediction of climatic vulnerability because we do not think reliable ones can yet be provided.”

**At the conclusion**

“In conclusion, data on patterns of species’ thermohydroregulation, aestivation, and field evaluations of presence in regions were models give opposing predictions, are badly needed before we can predict impacts of changes in the hydrothermal environment (e.g., through climate change, forest fires, or deforestation) on animal populations”

Several processes described in the 'Methods' section are written confusingly and repetitively. I suggest reorganizing the subtitles and describing the steps in the chronological order in which they occurred to facilitate understanding. Below are some specific comments on these topics and others related to writing.

Specific comments:

Lines 1-2: To extrapolate the results to "Amazonian lizards", theoretically, it would be necessary to have at least a sample of species representative of the whole spectrum of general characteristics that define Amazonian lizards. As this is a research with two thermoconformers species, the extrapolation becomes inappropriate. I suggest identifying in the title that the research is about two species or populations of thermoconformers lizards.

**A: We changed the title to “in two Amazonian lizards”.**

**As we show, thermoconformer is a traditional but not quite valid term to characterize species, as species may behave as thermoconformers only while they are not thermally challenged.**

Lines 4-6: Institution names in the native language (original names in Portuguese).

**A: We will try to correct these in the electronic online form, also.**

Lines 322-325: Please insert the number of collection sites for each species in the main text. Although the supplementary material has the coordinates of the sites, informing the number of sites will help the reader to visualize the extent of the data collected. For example, for L. percarinatum it was 5, correct? What about for L. ferreirai?

**A: 4 and 2, added.**

Line 333: The "Method" section begins with the subheading "Experimental procedures", which explains data collection in general. Next, the subtopics are presented: "Manipulation of lizards' hydration level" (line 340) and "Measurement of the voluntary thermal maximum" (line 363). It does not make much sense to separate the collection of dehydration information from VTMax, as both undergo the same treatments (fresh, dehydrated and rehydrated). As described, it is necessary to repeat the protocol information, such as the collection times. I suggest removing the cited subtopics and describing the processes all within "Experimental procedures" in the order in which both information is collected for each individual.

**A: Ok, we combined the text.**

Lines 341-351: This passage is confusing. Example: on line 341, the authors state that the lizards were kept in plastic, opaque and hermetic boxes containing rice (for desiccation), and later in line 345, it says that the first measurement was obtained in the morning on the first day. Start the paragraph by describing the measurement on the first day, then proceed to the desiccation phase in the box and consecutive rehydration.

**A: done**

Lines 343-344: Why were temperature and humidity collected every half hour? The reason for this needs to be clarified as written.

**A: we do not have any important reason for using 30 min instead of 60 or 15. We just automatically monitored the conditions within the boxes and arbitrarily selected 30 min.**

Lines 369-370: Reallocate the number of variations in results.

**A: done**

Line 381: Replace “São Paulo University” with “Universidade de São Paulo”. The institution name is a proper noun and does not need to be translated into English.

**A: done**

Line 398 -400: Relocate Shapiro-Wilks test results to the "Results" section.

**A: Although these are “results”, they are not the results of the tests proposed in the objectives, but of a secondary test on premises performed along the process of the application of the analysis. To make the suggested insertion clear, we would need to also include methodological options in the results section and pollute that section with information that does not correspond to the objectives, nor affect the conclusions. Thus, we opted to keep the text like it was, none of the previous reviewers showed concerns with this point.**

Lines 385-406: The "Analyses" section starts with a general description of the analyses and follows with subheadings explaining each statistical test in more detail. Line 386 of the "Analyses" section begins with a general description of each test. Later, on lines 403 to 404, it says, "For that, we fitted a linear mixed model in which body weight was the continuous response variable". This type of repetition of information leaves the text confusing and overwhelming. I suggest that the "Analyses" section start with a detailed description of each test (starting with the subtitles), and incorporate the information from lines 386 to 401. At the beginning of each subtitle, you can first explain the question to be answered, the variables used and the statistical test. Write in chronological order, aiming for simplicity, objectivity and avoiding confusion.

**A: done**

426 to 428: Is this transformation the same as in lines 398 to 401? If so, it is repetitive or confusing to mention it again. I suggest reorganizing the subheadings (as previously mentioned) and putting this information only once. As for the transformation, the mixed linear models are also presented more than once in the text.

**A: the previous correction should solve this problem, too.**

Figure 3: In lines 431 to 435, it is specified that the maps in figure 3 are only illustrative (due to data limitations) of how much dehydration can change the geographic risk models and highlight the importance of dehydration in the process. However, the caption presents the first piece of information: "Maps predicting present thermal risk for *Loxopholis percarinatum* across the amazon basin", emphasizing not the process but the actual places of thermal risk. Therefore, I suggest rewriting the first sentence of the caption as something like: "Map of thermal risk variation, based on the response of lizards to dehydration".

A. **rewritten as suggested**

Line 223: The cited reference (52) refers to a study about food items, microhabitat use, period of activity, reproductive cycle and fecundity of L. percarinatum. It does not address any issue related to thermoregulation.

**A: at line 223 we have: 52. Crawford EC Jr. Brain and body temperatures in a panting lizard. Science. 1972;177: 431–632 433. So it seems to be correct.**
