# Supplementary material for: Dehydration alters behavioral thermoregulation and the geography of climatic vulnerability in two Amazonian lizards
Source: PLoS One. 2023 Nov 1;18(11):e0286502. doi: 10.1371/journal.pone.0286502 (PMC10619801; doi:10.1371/journal.pone.0286502)
Supplement: Attachment — Submitted filename: third review plosone final.docx [file pone.0286502.s005.docx]

Dear Dr. Camacho,

After this second review round, one of the previous reviewers decided for the acceptance of your manuscript. Still, a new reviewer raised significant issues that prevent the publication of your manuscript at this time. Still, if you are able to correct and/or justify the issues raised by this second reviewer, both reviewers believe manuscript has a potential to be published in PLoS One after improvements are considered. Please resubmit your manuscript by May 13 2023 11:59PM or in an earlier date in case you are able to conduct the changes faster than I have previously imagined. Please do not hesitate to contact me in case you need further time or have any other doubt that needs to be taken care of.

Sincerely,
Daniel Silva.

Dear Daniel,

thanks for the chance to resubmit. Since reviewer number three has already expressed satisfaction, we move on to discuss the concerns voiced by reviewer number four.

\

Águs Camacho on behalf all authors

**Reviewer #4: This paper describes an experiment with two species leaf-dwelling, Amazonian lizard that were used in a laboratory-based study to examine how hydration-state influences voluntary maximum temperatures (VTmax). The authors used linear models to compare between hydrated, dehydrated, and rehydrated lizards to see how hydric state influence VTmax. The authors also included important factors such as heating rate and starting temperature to create their models. Additionally, they used the NicheMap package to make predictions about population risks depending on rainfall and temperature patterns in the Amazon. The manuscript may prove important to thermal biologists looking to include other important physiological variables (ie hydration) when predicting species responses to fluctuating thermal environments. The flow of the manuscript and reporting/presentation of the data need some improvement and I have made some specific suggested changes below as well as some general comments/concerns about major portions of the manuscript. Overall, I found this to be a compelling ms. However, my major concerns are with the logical flow of the introduction, your definition of hydrated versus dehydrated, and using the discussion so that readers understand the relevance of your results as a means to validate previous work or propose new frameworks. I commend the authors on already going through several rounds of review and I hope that they agree my suggested changes are feasible and improve the manuscript.**

We appreciate Dr. Brusch's review. To support earlier research or suggest new frameworks, we revised the introduction's thought flow, highlighted the distinction between hydrated and dehydrated animals, and tried to strengthen the discussion part. To learn more about how we achieved it, please read our answers to his remarks.

**General comments:**

**Introduction:
-Earlier in lines L33-34 it seemed as though this paper was focusing on physiological responses to thermal and hydric environmental conditions. Most of your intro reads as such too. Then around ~L92 the paper switches to focus on using environmental moisture as a factor to further explore thermal traits using a modeling approach. Then the final sentence switches back to focusing on hydration. Environmental moisture and available water to maintain hydric state (dietary water, drinking water, and in some cases metabolic water) are very different things and it is confusing to suddenly go back and forth. If you decide to keep this paragraph in the intro about ‘environmental water’ it should be physiologically-based.**

The introduction cannot be solely physiological because we are linking changes in physiological traits (VTmax and dehydration) to changes in ecological traits (vulnerability) in this study.

As a matter of fact, we start the introduction by writing about protocols to evaluate thermal vulnerability, for which thermohydroregulation data are needed. We must write about environmental water because it is a major source of hydration for lizards and other animals. Thus, we need this paragraph to introduce the modeling approach and the included variables in this study, which is why it does not look physiologically based, but rather “eco”. The paragraph in question is the last one right before the objectives, which obligately represents both the physiological and ecological variables of the study. That paragraph is precisely there to avoid excessive switching between topics along the introduction. Nonetheless, although we cannot prescind from the paragraph, we tried to give it a more physiological perspective. Hopefully, the reviewer finds the changes satisfactory.

**-Furthermore, your experiment uses categorical variables of hydric state (which are problematic, see general comment below in the results section) as factors to explain thermal conditions for lizards. No part of your experiment details hydro-regulation in these animals and I suggest removing most of the references to hydroregulation and instead frame your experiment as one where you consider the role hydration plays in animals abilities to acclimate, or not, to varying thermal constraints. This is a paper about factors that explain VTmax, as the title suggests, but starting in the abstract (L12) you introduce thermo-hydroregulation which is very different and involves organisms maintain homeostasis in regards to hydration and Tb (both separately and combined).**

Our categorical predictor represents three different hydration treatments, not hydric state: fresh (measured as collected), dehydrated, indicating that the animal underwent a dehydration treatment for one night, and rehydrated, indicating that the animal underwent a 24-hour rehydration period. We used a continuous variable, hydration level, as the percentage of the maximum weight measured for the animal across the three treatments. We do not see problems with this approach. Nonetheless, we made an effort to be more clear about this.

According to the definition of thermohydroregulation, by Rozen-Rechels, this term represents the interaction of hydration level and temperature in behavior. Since we are measuring the voluntary thermal maximum of each individual three times at different hydration levels, we consider we are observing thermohydroregulation. We are not observing acclimation as Dr. Brusch suggests since we are not keeping individuals under acclimation treatments, in which the state of the animal typically remains the same (i.e., same temperature) and different at each treatment level. Our treatments (fresh, dehydrated, and rehydrated) are dehydrating/rehydrating animals. Then, hydration level is used as an explanatory variable in the model to predict to predict induced changes in behavioral thermoregulation (Thus thermohydroregulation)

We agree that this paper shows factors that make the VTMax vary and how that variation translates into geographic predictions of thermal vulnerability.

Rozen-Rechels 2019 defines thermohydroregulation as integrating water levels and temperature in behavior. Since we are observing changes in the maximum thermal levels voluntarily tolerated, according to water levels, we (and reviewer 3, since he/she approved this ms for publication) consider that the observed behavior can correctly be termed thermohydroregulation.

**Results
-Please be consistent with significant figures throughout the ms**

Sorry, we are not sure what "significant figures" mean. We double-checked the text's usage in the names of the figures for the main figures (Fig) and the supplementary figures (Fig S).

**-It is unclear but appears as though hydrated is 95.0-100% HL and dehydrated is 0-94.9% HL. There is also never any definitive of HL. I assume that is hydrated level. And is it based on mass entirely? There is a definition of hydration level in L364-366 but it is not made clear.**

We changed HL to hydration level in all instances. We also clarified the explanation of HL to: “we simply considered hydration level as the percentage of each individual’s maximum mass obtained across the three measurements” (L 364-370). Thus, hydration level, used in the model predicting the VTMax, is a continuous variable, not categorical nor a group.

**Furthermore, This arbitrary grouping is problematic as there is no justification for why an animal is ‘hydrated’ at 95.0% but suddenly dehydrated when it reaches 94.9%. In this sense, that 0.1% difference has a major impact around 95% but means nothing when comparing 96.2% to 96.3%. Consider having a larger difference here of at least 1-2%. And is there any justification for why only a 5% decrease in body mass means that animals are dehydrated? Clinical levels of dehydration are typically only after 15-20% body mass loss. I’m glad to see the authors did not push the animals that much, but you need more in the text for why these animals are considered ‘dehydrated’.**

We did not compare animals at 96.2% to 96.3%. During the experiments, the grouping (fresh, dehydrated and hydrated) represents which of the three treatments defined in lines 509-511 had each individual been subjected to. Because these treatments altered the hydration state of each individual differently, we could later use hydration level as a continuous variable predicting the VTmax.

We only splitted animals into two hydration groups for mapping thermal vulnerability. The hydrated category takes the median weight and VTmax of animals with hydration level above 95%. The dehydrated category uses mean weight and VTmax of animals hydrated below 95% (i.e., from there down to the minimum hydration level achieved across our animals). Thus, again, we are not comparing animals at 96.2% to 96.3%, nor at 95% with 94%. As written:

“For this approach, we created two maps, one using the median mass and VTmax obtained from lizards over 95% of their fully hydrated mass, and another for the weight and VTmax of individuals dehydrated below the 95% of their fully hydrated mass. We call these two arbitrary groups “hydrated” and “dehydrated” for illustrative purposes only.”

**-It is unclear why Table 1 is included in the ms. It is only referenced once in the results along with separate suppl. Tables. Since it doesn’t appear to be important to the overall story (ie it isn’t mentioned in the discussion) consider moving it as a supplementary table.**

A prior referee requested the inclusion of this table. Because there is a margin and the third referee agrees. We'd rather leave it at that. In addition, direct references to the results' figures and tables in the discussion section are frequently discouraged.

**Results/Discussion
-Similarly, Figures and 1 and 2 are only mentioned once each in your results. The discussion is an important chance for you to put your results in the context of previous work and allow you the change to justify any of your claims with evidence. Yet, the only figure or table you mention is figure 3 twice (L255 and L295). If you keep table 1, and figs 1-2 in the manuscript they need to be included in the discussion so that readers understand the relevance of your results to validate previous work or propose new frameworks (as you do in L273-277). As written, I see no reason to have anything except figure 3 in the final manuscript.**

Figures 1-2 show the efficiency of the treatments on hydration levels as well as the influence of the main factors tested experimentally. As a result, including them in the results section appears to be necessary to display to the readers the evidence that is available. Regarding referring figures in the discussion part, we followed tutorials that recommended the contrary

<https://www.sfedit.net/how-to-write-a-discussion-section-for-a-scientific-paper/#:~:text=The%20discussion%20section%20is%20not,presented%20in%20the%20results%20section>.

We nonetheless recognize there is a diversity of opinions on the matter <<https://academia.stackexchange.com/questions/42032/referencing-figures-or-tables-in-discussion-section>> being the one we took potentially the dominant one: reference to figures and tables should be made in the results section only as in Plos One’s manuscripts <https://journals.plos.org/plosone/article?id=10.1371/journal.pone.0271076 & https://journals.plos.org/plosone/article?id=10.1371/journal.pone.0261173>.

Given the fact that ref 3 has already approved the ms, we would wish to avoid incorporating changes that may result in new objections.
**Discussion
- L199-202 and throughout the discussion- It is unclear why this is being presented as one-sided. As written, you suggest that animals become dehydrated which lowers their VTmax. It could just as easily be explained as animals choose as higher VTmax but it comes at the cost of dehydration. Both potential scenarios need to be considered, especially considering your metric for dehydration (~%5 loss in body mass) might not signify an ecologically relevant level of water loss. For example, a normosomic animal might behaviorally select a higher temperature to increase metabolism but at the cost of increased water loss. These animals then may retreat to cooler areas to avoid dangerous levels of water loss or even to rehydrate so they can return to warmer locations. The discussion is written very thermal-centric, which is fine, but you should at least consider this entire story from an equally hydric lens.**

Since Dr. Brusch is referring here to different items, we respond them one by one:

**As written, you suggest that animals become dehydrated which lowers their VTmax. It could just as easily be explained as animals choose as higher VTmax but it comes at the cost of dehydration.**

Yes, we suggest that. The results strongly indicate for both species that VTmax increases as hydration level does (See figure 2 and Table 1). In turn, we did not evaluate the dehydration costs at different temperatures, so we cannot say that animals choose as higher VTmax but it comes at the cost of dehydration.

Specific Comments:

**L34- Please provide reference(s) for physiological performance being influenced by hydration. I assume the physiological performance being influenced by temperature is specific to ectotherms but that is not clear in this sentence. It is also unclear if you mean both temperature and hydration (as the example you give suggests) or separately and combined. Please clarify.**

We included “Ectothermic animals” in the sentence and clarified, providing the requested references:

“Also, while dehydration may alter ectotherms’ growth rates and behavior [10], thermal and hydric stress combined may decrease reproduction and survival [11].”

**L35- I should be capitalized and i.e. should be in italics as it is latin**

Done
 **L37-39: reword this sentence. Metabolic rates can definitely increase but EWL isn’t necessarily as closely related in squamates, or even across ectotherms. This might be specific to tropical forest species, if so this needs to be stated clearly.**

We changed our sentence, adding a “can” to ease the tone: “As body temperature rises, evaporative body water losses can increase exponentially [8], like metabolic costs [9].”

**L39: remove this generalization with ecotherms because it simply isn’t true. There are numerous studies demonstrating species resilience when faced with higher temperatures and dehydration.**

Since the references in the original version demonstrate that heat stress and dehydration may have a significant impact on growth rates, reproduction, and survival, we see no problem with this sentence. We are not generalizing that all ectotherms will have their growth stunted by thermal stress and dehydration; we are merely stating that it is possible. Thus, we stated: “Also, when combined, heat stress and dehydration may strongly impair ectotherms’ growth rates, reproduction, and survival [10,11]. “

We did not refer to “resilience” in that track of the text, which has a different meaning in ecology.

**L41- remove ‘such’ from this sentence**

removed

**L43- this portion is confusing, please reword and clarify what you mean by animals “needing” higher temperatures. Do you mean they don’t start to pant or urinate until temperatures are higher than normal because they’re dehydrated?**

We followed Dr. Brusch’s advice:

“When ectothermic animals, as lizards, exhibit thermoregulatory behaviors, they likely integrate information on their hydration level (*i.e.* they actually thermohydroregulate) [12]. For instance, dehydrated lizards required exposure to higher temperatures to induce the onset of water-demanding thermoregulatory behaviors, such as panting or urinating [13–18]. Notwithstanding, these two behaviors may represent emergency responses (reviewed in Tattersall et al., [19]).”

**L45- It is confusing to suddenly have an example with just thermoregulation in between sentences with thermohydroregulation. Please re-order.**

Please, see above.

**L50- consider re-writing so that it reads “…water stress could help us understand how climatic restrictions may influence their activity and distribution.”**

We considered it, but since “climatic restriction” is an influence of climate on activity and distribution, the combination of “climatic restriction may influence” seems redundant.
**L52- the previous sentence stresses the importance of considering both hydric and thermal constraints. But then this sentence emphasizes just a thermal trait measurement. Re-word this sentence to avoid conf”usion.**

We changed the sentence to:

“The voluntary thermal maximum (VTmax) is a thermoregulatory trait that has been shown to integrate both thermal and water levels in frogs [21].”

**L62- it is not clear, as written, the “them” that these typical thermoconformers are responding to. Oxygen conentrations? Typical thermal assays? Please clarify**

Thanks, we replaced them by “heating rates and start temperatures”.

**L64- change “would” to ‘could’**

Changed.

**L66- The saline injections needs to be clarified or removed. If you keep it, you need to define what you mean earlier in the introduction by dehydration. Often times it is simply referring to hyperosmolality unless you want to define it as hypernatremia, as is suggested by this reference/sentence.**

We clarified: “Previous studies in desert lizards [14] have found no effects of dehydration and starvation on the VTmax, although this parameter may vary after the injection of saline solutions, a different way to alter plasma osmolality levels.”

**L68- consider removing “magnitude and” to make this sentence crystal clear.**

Thanks, done.

**L69-71- this just means that some species VTmax is at or very close to their CTmax** **L72-76- you need to at least address species specific VTmax versus CTmax. It’s exclusion here is confusing. Especially considering the next paragraph starts with thermal safety margins and a mention of CTmax. I understand not taking up too much space in this paper for brevity but your justification (L69-71) for species living in potentially dangerously warm environments seems like cherry picking.**

This is not the case for *Chalcides ocellatus*, which, despite being quite close to its CTmax holds up 24h; this species is now included as an example. CTmax is unknown for most species with survival time at VTmax, which are very few. So, that would be guessing upon the multiple factors that may increase the time for survival for some species compared to others, and that deserves a full study in itself. Also, if there were any other data on survival time at the VTmax for other species that changed the story, we would gladly add it here. We think the message here is clear, the Vtmax may induce function loss and death in a matter of hours.

**L86- Please define or explain what you mean by ‘dry-skinned ectotherms’ I assume snakes and lizards**

We clarified: “squamates, terrestrial arthropods”

**L87- change to “might bury themselves within”**

Thanks, done.

**L129-130 AND L138-139: I would prefer if you included descriptive stats here. However, they can also be found in Table S2, which is okay. If you don’t include descriptive statistics here, please at least include something like “(all p<0.05)” since you use ‘significantly affected by’**

We included the suggested “(all p<0.05)”

**L132 and throughout- include units for the mean body mass, I assume 0.694 g. (also on L134 an a few other places in the results).**

Thanks for pointing that, done.

**L151- what are negative thermal margins? I can’t find in the methods what this means, please explain somewhere in the manuscript.**

We synonymized “Thermal margins” with warming tolerance margins, which were explained in the methods.

**L168-170- as written this is confusing, consider re-writing so that it reads “The extent and duration of thermally stressful events, and the percentage of lizard populations affected by them, increased by several orders of magnitude as VTmax decreased, and were associated with dehydration more than any other parameter.”**

Thanks, replaced.

**L174- change to “had a larger affect”**

Changed.

**L176- change to ‘changes in mass associated with dehydration”**

Changed.

**L176- I assume this is in reference to hydration in animal who are 10cm deep and not dehydration in general? Please make sure this is clear here.**

No, burying 10cm deep and being dehydrated or not are independent conditions included in the models that generate the maps.

We rephrased to: “Neither the ability to bury down to 10 cm nor changes in mass associated with dehydration did not observably affect our thermal risk metrics.”

**L196- what is meant by internal environment?**

We wrote: They attributed these changes to internal rhythms or rhythms of the external environment.

We meant to separate internally set rhythms and changes in the environment that surround the animal. We think that what the authors wrote is transcribed to the best of our possibilities.

**L207- what is the “receptively” about? If the citations, then it needs to be within the brackets.**

We moved “respectively” to within the brackets.

**L209-219- unless you have tropical/temperature lizard-specific references here, remove metabolic water as that is typically negligible in reptiles except in some cool examples of desert herbivores/insectivores.**

Lowering temperature and hence metabolic rate necessarily lowers metabolic water production. Therefore, even though it has yet to be determined whether or not metabolic water production is essential for survival across lizard species, the argument is still sound.

To address Dr. Brusch’s concerns, we clarified: “However, the absolute performance of traits, like locomotion, hearing, digestive and immune efficiency, and the rates of metabolic water production (ex. in desert-adapted reptiles), can decline as well (reviewed in [9,12,49]).”

**L216-240- It is unclear what this paragraph adds to the discussion. This paragraph details studies in other species (large, long-lived desert lizards) that are very different from the two tropical species used in your experiment (small, short-lived leaf litter lizards). Consider removing it entirely for brevity.**

This is a significant portion of the discussion with which the other reviewer has completely concurred with all that has been said. We believe that it gives our findings a more general application and points the way for further research. As a result, since the text is within the size requested for plos one articles, after considering it, we decided to retain it.
 **L226- Please see work by Hazard (e.g., 2001, PBZ) to update these sentences. Desert iguanas have salt glands which is not relevant to the two species your experiment used.**

We updated the text as requested: “Still, desert iguanas did alter their VTmax in response to injections of concentrated saline solutions, leading to osmotic imbalance. Yet, desert iguanas have especially efficient ways to keep the ionic balance under heavy loses of body water, like salt glands, renal secretions, and water stored in their thick tails [14, 82].”

**L245-246- osmotic pressure is measured in the plasma portion of blood. Similar to my comment on L66, it is unclear here the difference between hyperosmolality and ‘dehydrating processes’**

We changed it to “temperate lizard species indicate that they maintained their maximal body temperatures either after experimental saline injections, or during dehydrating processes [15,16,23,43,47,55].”

**L256- what is hydration rate? That is not mentioned anywhere in the manuscript and it is unclear what you are referencing. How fast they can absorb water in the GI tract? Water loss rates? Please clarify**.

We replaced “rate” by “level”.

**L273- You did not measure plasma osmolality so this statement is not justified**

We eased the tone and supplied a case study as follows:

“Based on the discussed lines of evidence, we propose that the VTmax of lizards, and possibly other ectothermic species, might be relatively more reactive to dehydration when the measured species: 1) presents less effective mechanisms for controlling plasma osmolality (e.g., salt glands),”

**L274- the only known lizard with a water reservoir is the Gila Monster who can use their urinary bladder, similar to desert tortoises. Having a greater total amount of body water (because of being large bodied) does not equal a meaningful amount of great total body water as this value is proportional and typically 65-75% across terrestrial vertebrate taxa.**

We clarified and added the reference for the gila monster: “This could allow them to maintain activity, with the resultant water ingestion, if available [54]. We hypothesize that such a strategy might be especially effective for organisms with special water reserves, such as the bladder (ex. the Gila monster [83]), and/or for species with low specific water loss rates (*e.g.*, large desert lizards or tortoises [43,47,55]).”

**L285- if you use quotations, please add a citation directly after so readers know who you are quoting here.**

We removed the quotation marks because we are not citing anyone and provided a more in-depth explanation of the phrase: “However, interspecific variation in the magnitude of this thermal fear zone (*i.e.,* between the VTmax and the CTmax), “

**L322- change to “…who live over and within…”**

This suggestion was deemed an error by the grammar checker, so we changed it to.: “*Loxopholis ferreirai,* is a semiaquatic and scansorial lizard species that lives over and within logs in flooded “Igapó” forests across the course of the Rio Negro and tributaries [60].”

**L354- is this absolute humidity or relative humidity?**
Thanks, we added “relative”.

**L374- it is unclear what you’re trying to say here, all individuals, except two, stayed in the can? Please re-word for clarity.**
Reworded:

“Before heating, lizards were left for a couple of minutes to assess whether they were willing to abandon the heating can or if they took it as a refuge. This procedure led to excerpt two individuals which were visibly unwilling to remain in it.”
**change to “…tape wrapped around the side…”**

Thanks, changed.

**L407- change “starting temperature, heating rate, and body size.”**
Done.

**L408- change “Besides” to ‘additionally’**

Done.

**L410- change “a reviewer’s” to ‘potential’**

Done.
